# Development of a Novel Rabies Simulation Model for Application in a Non-endemic Environment

**DOI:** 10.1371/journal.pntd.0003876

**Published:** 2015-06-26

**Authors:** Salome Dürr, Michael P. Ward

**Affiliations:** Faculty of Veterinary Science, The University of Sydney, Camden, New South Wales, Australia; Swiss Tropical and Public Health Institute, SWITZERLAND

## Abstract

Domestic dog rabies is an endemic disease in large parts of the developing world and also epidemic in previously free regions. For example, it continues to spread in eastern Indonesia and currently threatens adjacent rabies-free regions with high densities of free-roaming dogs, including remote northern Australia. Mathematical and simulation disease models are useful tools to provide insights on the most effective control strategies and to inform policy decisions. Existing rabies models typically focus on long-term control programs in endemic countries. However, simulation models describing the dog rabies incursion scenario in regions where rabies is still exotic are lacking. We here describe such a stochastic, spatially explicit rabies simulation model that is based on individual dog information collected in two remote regions in northern Australia. Illustrative simulations produced plausible results with epidemic characteristics expected for rabies outbreaks in disease free regions (mean R_0_ 1.7, epidemic peak 97 days post-incursion, vaccination as the most effective response strategy). Systematic sensitivity analysis identified that model outcomes were most sensitive to seven of the 30 model parameters tested. This model is suitable for exploring rabies spread and control before an incursion in populations of largely free-roaming dogs that live close together with their owners. It can be used for ad-hoc contingency or response planning prior to and shortly after incursion of dog rabies in previously free regions. One challenge that remains is model parameterisation, particularly how dogs’ roaming and contacts and biting behaviours change following a rabies incursion in a previously rabies free population.

## Author Summary

Rabies in domestic dog populations still causes >50,000 human deaths worldwide each year. While its eradication by vaccination of the reservoir population (dogs and wildlife) was successful in many parts of the world, it is still present in the developing world and continues to spread to new regions. Theoretical rabies models supporting control plans do exist for rabies endemic regions; however these models usually provide information for long-term programs. Here, we describe a novel rabies simulation model for application in rabies-free regions experiencing an incursion. The model simulates a rabies outbreak in the free-ranging dog population in remote indigenous communities in northern Australia. Vaccination, dog density reduction and dog confinement are implemented as control strategies. Model outputs suggest that the outbreak lasts for an average of 7 months and typically spreads through all communities of the region. Dog vaccination was found to be the most effective response strategy. The model produces plausible results and can be used to provide information for ad-hoc response planning before and shortly after rabies incursion.

## Introduction

Rabies is among the most lethal infectious diseases, present on all populated continents except Australia [1]. The domestic dog remains the most important vector worldwide, causing >95% of all human cases [2–4]. Despite availability of an effective vaccine for more than a century and repeated demonstration that vaccinating the domestic dog population is the most effective way to eliminate the disease [5–8], rabies remains endemic in large areas in Africa and Asia. Moreover, the disease has (re)emerged in areas previously free (such as Bhutan [9,10], Indonesia [11,12], and the Central African Republic [13]).

Rabies continues to spread through the Indonesian archipelago via human mediated domestic dog movements [11,12,14], most recently through the previously rabies-free province of Maluku in eastern Indonesia [11,15]. The risk of incursion into rabies-free areas − Timor, Irian Jaya, Papua New Guinea (PNG) and northern Australia − is therefore high. Possible incursion scenarios into Australia include yachts or fishing boats hosting latently rabies infected dogs traveling from Indonesian islands to remote areas in northern Australia [16]. Also, close cultural ties between PNG and Torres Strait Island communities exist, increasing the risk of movements of dogs incubation rabies from PNG to Australia, if an incursion in PNG occurs [16]. In these regions large, free-roaming domestic dog populations [17,18] increase the risk of rabies establishment, which would subsequently impact human and wildlife populations. Because there are no historical precedents, the spread and final impact of such rabies incursions is difficult to estimate. However, such knowledge is critical to informing preparedness and response plans prior to an incursion, and to design the most effective strategies.

Descriptions and applications of several rabies models in wildlife [19–24], domestic dogs [5,7,25–28] or a combination of these [8,29] have been published. All have been based on empirical field data in rabies endemic regions and typically aim to inform policy on reducing rabies prevalence and thus impacts. However, for a region in which rabies is exotic, predictions of the effectiveness of different interventions following the initial detection of rabies are more relevant. An issue is how rabies behaves when introduced to a previously free population, particular the effect of rabies on contact rates and biting rates. Evidence on these behaviour changes from previous rabies incursion may serve as an approximation but is typically vague and therefore equivocal. To our knowledge, epidemic models simulating rabies invasion in regions never exposed to rabies do not exist, a barrier to rabies preparedness planning.

Here, we describe the development of a novel simulation model of rabies epidemics in domestic, free-roaming dog populations in remote Indigenous communities in northern Australia–as an example of the potential scenario in many regions of the world where rabies is absent but where the risk of a rabies incursion is present and its spread is likely due to large populations of free-roaming domestic dogs. Results of a systematic sensitivity analysis are also presented and model application options are discussed.

## Materials and Methods

### Ethics statement

Data collection required to estimate model parameters has been approved by the Human Ethical Committee of the University of Sydney, grant no. 2013/757 and the Animal Ethical Committee of the University of Sydney, grant no. N00/7-2013/2/6015.

### Study site

The rabies simulation model development was based on data from two distinct regions in northern Australia, the Northern Peninsula Area (NPA) of Cape York, Queensland and Elcho Island, the Top End of the Northern Territory. Characteristics of the two study sites are presented elsewhere [18]. Briefly, in the NPA five Aboriginal and Torres Strait Islander communities are located in close proximity (2−4 km) to each other. On Elcho Island one larger Aboriginal community is present. The dogs are typically owned but unrestrained and build a large population in all communities (human:dog ratio 2.7−8.8 per community, Table 1) [18]. The dog population in the NPA − which informs the simulation model − is based on the most recent dog census conducted by the NPA Regional Council in 2009. As such information was not available from Elcho Island, the number of dogs are calculated based on the average human:dog ratio of the NPA communities and official human census data from Elcho Island in 2011 (http://www.censusdata.abs.gov.au/census_services/getproduct/census/2011/quickstat/SSC30094) and similar household sizes as in the NPA are assumed.

**Table 1:** Characteristics of the communities included in the rabies simulation model.

	Bamaga	Injinoo	New Mapoon	Seisia	Umagico	NPA^a^	Galiwinku
Area (km^2^)	0.963	0.284	0.42	0.309	0.226	2.2	1.267
Human population^b^	1046	468	274	203	281	2272	2124
Total number of households	286	131	176	149	74	816	264
Number of households with dogs	71	20	31	21	32	175	163
Density of households with dogs (households/km^2^)	74	70	74	68	142	80	129
Number of dogs	132	53	91	51	102	429	410
Density of dogs (dogs/km^2^)	137	187	217	165	451	195	324
Average number of dogs per dog-owning household	1.9	2.7	2.9	2.4	3.2	2.5	2.5

^a^NPA (Northern Peninsula Area) consists of the communities Bamaga, Injinoo, New Mapoon, Seisia and Umagico

^b^based on http://www.censusdata.abs.gov.au

### The simulation model

The model developed is stochastic, spatially explicit, based on individual dog data and assumes a daily simulation time unit. It starts with the introduction of a latently (non-clinical) infected dog and ends when no infected dog remains. The exact location of each dog’s home is known and a closed dog population within the region is assumed, but dog movements between regional communities are simulated. In the model, 429 and 410 dogs in 175 and 163 households are included in the NPA and on Elcho Island, respectively (137−451 and 68−142 households per km^2^, respectively, Table 1). The two regions are simulated separately. The average number of dogs per dog holding household ranges from 1.9–3.2 per community.

#### Input and output values

Information on all dogs included in the model is stored in a single data frame containing their disease states (susceptible, exposed and latently infected, subclinically infectious, rabid and dead) and their status on rabies detection, culling (including reason) and rabies vaccination. Model parameters (S1 Table) are defined as either beta-pert distributions (minimum, mode, maximum; http://www.vosesoftware.com/ModelRiskHelp/index.htm#Distributions/Continuous_distributions/PERT_distribution.htm), uniform distributions (minimum, maximum) or fixed values; except for the distance kernel (see next section).

For each model repetition (i.e. each simulated outbreak), stored output information includes a list of the user defined parameter values, index dog(s) information, a table of the daily number of exposed, rabid, dead, vaccinated and culled dogs and dogs moved between communities and information on contacts within and between households. The history of all dogs infected with rabies, culled, vaccinated or moved, a list of the outbreak’s summary measures (duration, number of rabid, culled (per culling type), vaccinated and moved dogs), daily maps of the location of contacted, exposed, rabid, vaccinated and culled dogs, an epidemic curve and a graph of the number of secondary cases for each dog developing rabies over the duration of the outbreak are also stored.

#### Disease states and rabies transmission

**Fig 1:**
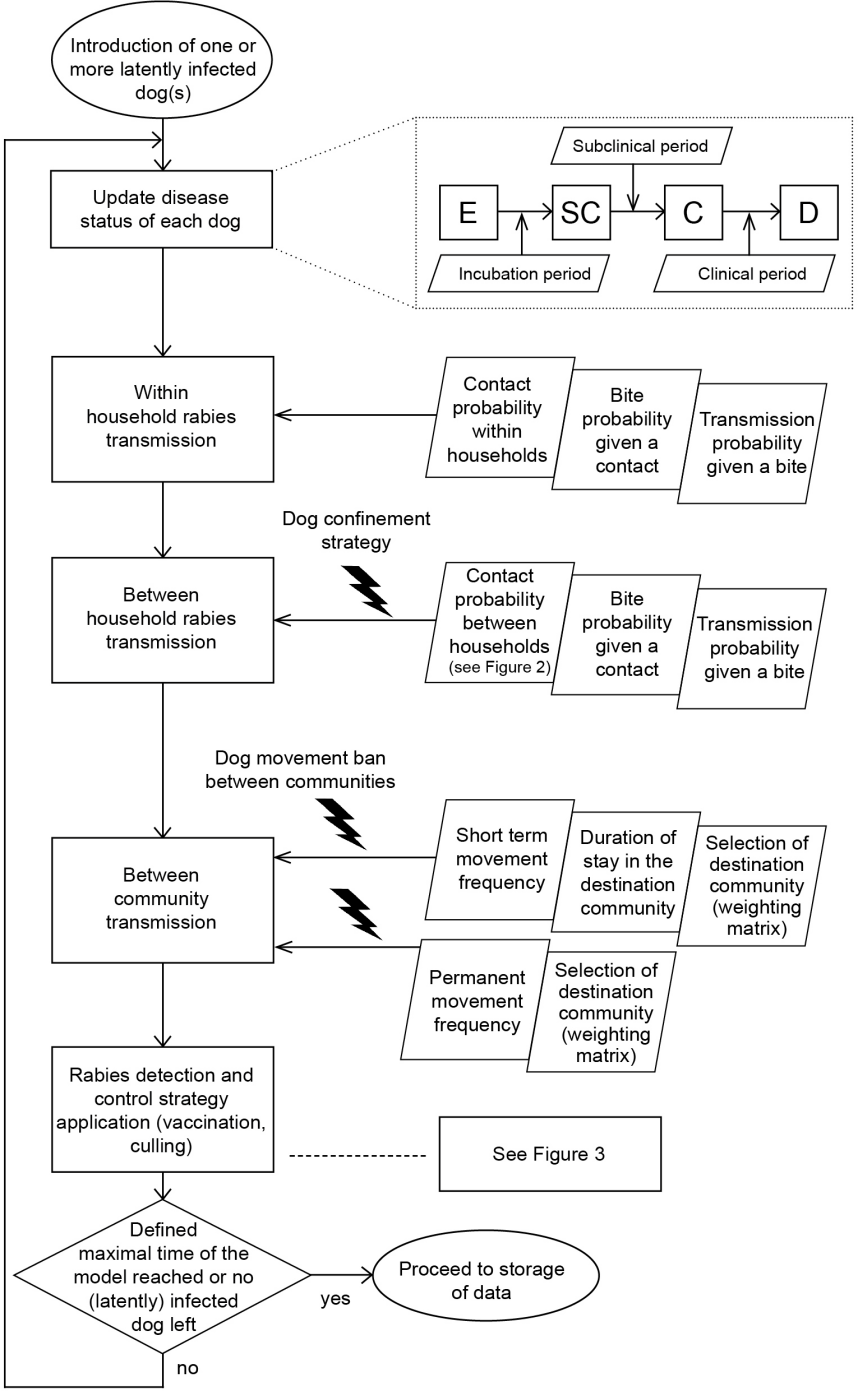
Daily model simulation actions in chronological order. On the right side of the figure the parameters used for each action are presented. For the update of the disease states each rabies exposed (E) dog develops subclinical rabies (SC), clinical rabies (C) and finally dies because of rabies or culling (D). At the end of each day, the model checks whether the criteria to stop the simulation are met or whether the loop will be repeated the next day. Dog confinement strategy and between-community movement ban are timely applied during the between-household and between-community contacts, respectively.

At the start of the simulation a user defined number of index dogs is chosen randomly, or alternatively a specific dog can be defined as the index dog (to test different control strategies assuming identical starting conditions). Every simulated day, the model chronologically loops through simulation actions (Fig 1) and ends when either the user defined maximum time has been reached (irrespective of whether the outbreak has ended) or when no (latently) infected dog remains.

First, the disease status of all dogs is updated. Each latently infected dog proceeds through the disease states subclinically infectious, rabid (i.e. infectious and clinical) and finally dead (unless culling or vaccination are applied). The time periods from one state to the next are defined (as stochastic parameters) via incubation, subclinical and clinical periods, respectively.

**Fig 2:**
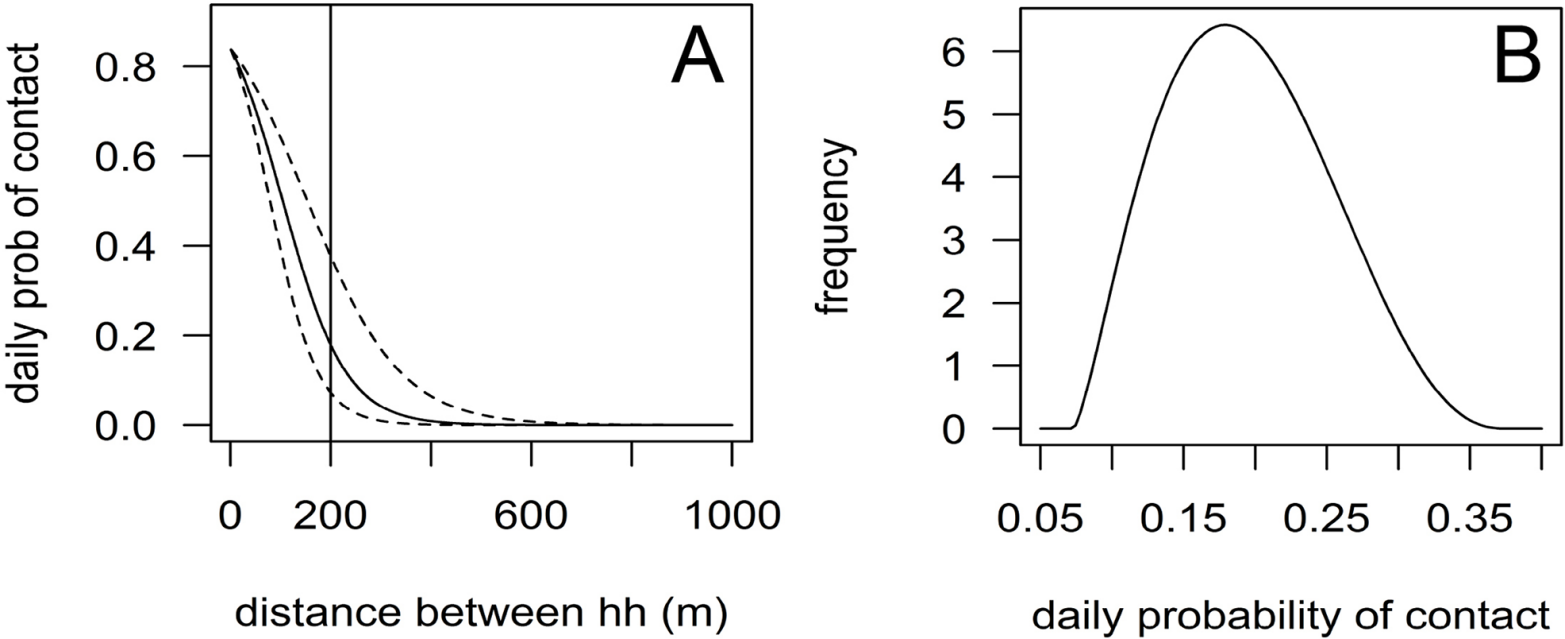
Distance kernel for contact between dogs from different households. (A) The daily probability with mean (straight line) and confidence intervals (dotted lines) of two dogs having a contact depends on the distance between their homes. (B) The probability of a daily contact for dogs living 200m away from a rabid dog (vertical line in (A)) is sampled from a beta-pert distribution with the mean of the kernel function as mode and the lower and upper confidence limits as minimum and maximum. prob = probability, hh = household.

Second, the three types of rabies transmission are simulated. This begins with transmission to susceptible dogs living in the same household as rabid dogs, which depends on the probability of contact with the rabid dog, the probability of being bitten given the contact and the probability of rabies transmission given the bite. While the default values of the first two probabilities are >80%, the rabies transmission rate is about 50% (S1 Table). Only a successfully exposed dog becomes latently infected; dogs with failed exposure remain susceptible and have the same risk of being infected the following day. Next, rabies transmission to dogs living in a different household is simulated, which depends on the distance between the rabid dog and the susceptible dog (Fig 2). The daily probability *p*_*c*_ that a susceptible dog living *i* meters from the rabid dog will be contacted is beta-pert distributed with a mode of

$${\text{p}}_{c}\;\left(\text{mode}\right)=1/\left(1+e^{-\left(\alpha +\;\beta i\right)}\right)$$ (1)

and a minimum and maximum of

$${\text{p}}_{c}\;\left(\text{min},\;\text{max}\right)\;=1/\left(1+e^{-\left(\alpha +\;\left(\beta \pm 1.96\beta_{se}\right)i\right)}\right).$$ (2)

The distance kernel is defined by the variables α (intercept), β (regression coefficient) and β_se_ (standard error of β). α denotes the probability of a daily contact between two dogs living zero meters apart from each other. However, as the contacting (and rabies transmission) of dogs living in the same household is simulated separately as described above, the value of α is only used to determine the highest possible daily contact probability. The regression coefficient β influences the shape of the kernel, while a larger negative value implies a steeper curve with more rapid decrease of contact probabilities with increasing home distances. β_se_ impacts the variance of contact probabilities for a given distance with increasing variability for increasing β_se_ value. Similar to rabies transmission within the same household, the success of disease transmission to a dog contacted outside the household depends on the probability of a bite given a contact (by default, a lower value than within-household contacts, namely 60−80%) and the probability of rabies transmission given a bite. Finally, rabies transmission between communities is simulated reflecting human-mediated movements of dogs, when (latently infected) dogs are transferred from one community to another. Two types of between-community dog movements are incorporated: permanent–for example the sale or gift of a dog or permanent movements of owners together with their dogs − and short term movements, for example during hunting trips or peoples’ visits between communities together with their dogs. The length of stay is user defined but intended to be short with a default value 1 to 2 days. In both types of between-community movements, only latently (non-clinical) infected dogs are considered because only these dogs are relevant for rabies transmission. We assumed that clinical dogs will not be moved to other communities since they show non-normal behaviour. Daily probabilities of rabid dog movement is community-specific and the destination community is chosen randomly with user defined weights (default value: the chance that the destination is a neighbouring community is twice as high as for any another community). Short term movements only have an effect on the outbreak in the situation when the moved dog becomes (or remains) infectious during its stay in the destination community. Only then will the new community become infected. The rabid dog that has been moved is considered to stay in a randomly selected household in the destination community until its death.

#### Rabies detection and control strategies

**Fig 3:**
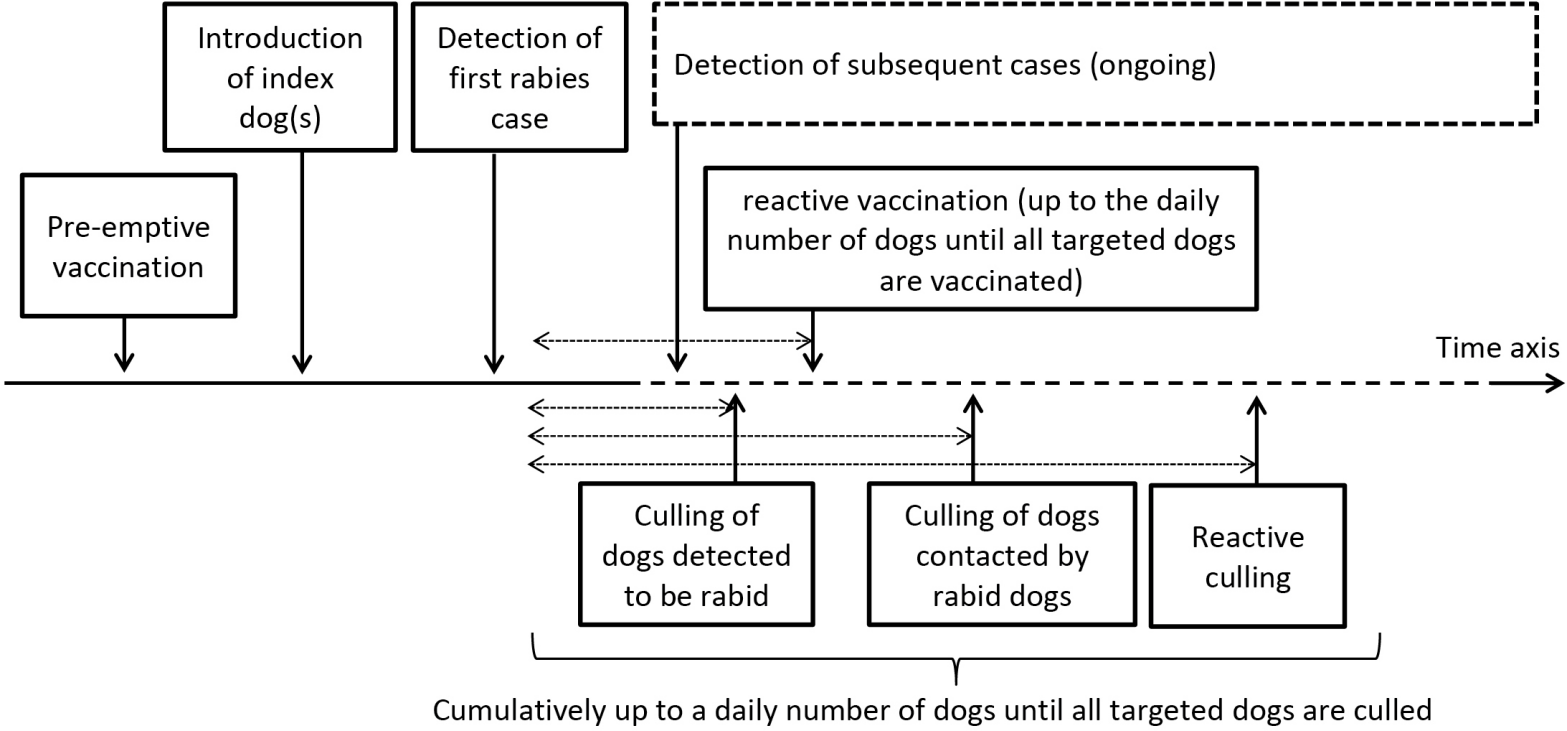
Rabies detection and vaccination and culling strategies. The starting order of the strategies in case applying more than one intervention may change (with the exception of pre-emptive vaccination), according to the user defined time delay between the detection of the first rabies case in the region and the start of the respective control strategy (dotted arrows).

Within the model, detection of rabid dogs and application of vaccination and culling are simulated after the spread of rabies, whereas dog confinement measures are implemented directly at the stage of between-household and between-community rabies transmission (Figs 1 and 3). The detection of the first rabid dog in the region is considered to take substantially longer than the cases following, because disease awareness will increase after the confirmation of the first rabies case. Detection of subsequent cases after the confirmation of the first case is assumed to be based on clinical signs with or without subsequent laboratory confirmation. Two vaccination, three culling and two dog confinement strategies − which can be selected either individually or in any combination − are implemented in the model.

Vaccination: Reactive vaccination (RV) starts after the detection of the first rabies case with a user defined delay that includes the decision making process, purchase and delivery of vaccine and organization of the vaccination campaign. As soon as the campaign has commenced, dogs up to a maximum value (limited by personnel resources) are vaccinated with priority given to those dogs in households closest to those in which rabid dogs have been detected. In case the daily limit has been reached, vaccination of the remaining dogs is postponed to the next day. The targeted extent of the campaign (infected community or region) and vaccination coverage level (either % of dogs or % of households vaccinated) can be defined by the user. Only dogs not yet detected as rabid are considered for vaccination. After a user defined time delay the vaccinated dogs are protected against rabies unless they become rabid in the meanwhile. As the vaccine efficacy is not considered to be 100%, protected dogs still have a remaining (although small) risk of being infected. This risk substantially increases (thus vaccine efficacy decreases) in the situation in which the dog was vaccinated a certain time after being exposed to rabies. Alternatively, pre-emptive vaccination (PV) can be chosen as the control strategy. Here, a given % of dogs or households in a targeted community or region are considered to be protected before the index dog with latent rabies infection is introduced. Again, vaccine efficacy is considered to be <100%, consistent with the RV strategy.

Population reduction: Culling of dogs the day they are detected rabid (DC) can be selected as a control strategy, starting after a user defined delay following the first rabies case. Dogs (up to a defined maximum daily number, limited by personnel resources) are culled immediately following detection. This reduces the infectious period in these culled dogs. An alternative approach (starting after a user defined delay) is to randomly select a given % of dogs contacted by detected rabid dogs (CC) to be culled on the day of exposure, as long as the daily culling limit has not been reached. Otherwise, these identified dogs are culled on the next day. Alternatively, or in addition to the culling of contacted dogs, a reactive culling (RC) control strategy can be implemented after a user defined delay. Similar to the reactive vaccination campaign, a defined % of dogs in the infected community or region are culled, with priority given to those dogs living closest to detected rabid dogs. Again, the cumulative number of daily culled dogs is limited so that for the reactive culling strategy, only the dogs remaining after the application of the other activated culling strategies can be culled on a given day. Culling of all other dogs will be postponed to the next day.

Control via movement ban and dog confinement (MB): Two types of dog confinement can be chosen as a control strategy: a ban on dog movements between communities, and confinement of dogs in the yard of the household (e.g. via fence or chain). The dog movement ban between communities starts after a defined delay following the detection of the first rabies case with a given level of compliance, resulting in a random reduction of between-community movements. This is applied to both of the two simulated movements, short term and permanent. The latter option reflects the policy of “keep your dog in your yard” and is implemented by truncation of the distance kernel to the community individual median distance between each household and its closest neighbour. Again, compliance is considered to be <100% so that a randomly selected % of dogs remain free-roaming.

### Parameter value estimation

Parameter value definition is a critical component in modelling studies, driving the outcome of any simulation or mathematical model. While some parameter values can be taken from the literature (e.g. disease or vaccine related parameters), other parameter depend on the settings in the specific environment in which the model was developed or applied. Seven out of 37 (19%) parameter values of the model presented here are sourced from the literature (those of rabies virus and vaccine related parameters) and 23 (62%) are based on assumptions (S1 Table). The latter can further be classified as experimental parameters (parameters defining control strategy implementation as e.g. delay in starting control strategies or vaccination coverage, 16/37 [43%]) and parameters for which value information are currently lacking (e.g. bite probability given a contact, owner compliance to cease dog movements, 7/37 [19%]).

The remaining seven (19%) parameter values were estimated based on our field collected data, including contact data within and between communities and mean distance between households used for dog confinement strategy (S1 Table). Data used to calculate the distance kernel function applied for contact rates between dogs of different households was derived from a large scale GPS study on 69 domestic dogs in all of the six communities (S5 and S6 Tables) [18]. The number of contacts between each pair of dogs within the same community was extracted using the definition of contact being within 20 meters during the same minute. As the model runs on a daily basis, the contact information was converted into a binary variable with two dogs having at least one (1) or no (0) contacts within 24 hours. This binary outcome was analysed by a logistic regression model with the known distance between the two dogs’ homes as the explanatory variable. The outcome variables estimated by the logistic regression (intercept α, coefficient β and standard error of the coefficient β_se_) were further used to build the distance kernel (Eqs 1 and 2). Daily contact probability of two dogs living in the same household was estimated based on the same dataset plus similar data collected during the post-wet season (monsoon) in the same communities. One of 31 (3%) pairs of dog living in the same household was not observed to have at least one contact per 24 hours. This within-household contact probability was implemented as a uniform distributed parameter with 97% as the mean.

Four parameters defining the dog movements between communities − both short term and permanent − were estimated from questionnaire data collected in the NPA (S2 Table, approved by the Human Ethical Committee of the University of Sydney, # 2013/757) together with observations of short term movements by GPS and of permanent movements of dog owners from one NPA community to another during a year. Twenty-nine dog owners were interviewed in September 2013 and one in September 2014, including questions on frequency of dog movements to other NPA communities due to pig hunting or other trips (e.g. visits or work). A daily movement probability of 0.058 per dog was calculated from these reported data, while from the study we observed that only 8 of 81 (10%) dogs were moved during an observation period of 6.2 days, resulting in daily movement probability of 0.016 per dog. Combining these reported and observed data and giving twice the weight to observed data, 0.03 was defined as the beta-pert distribution mode for daily short term movement probability per dog; 2- and 0.5-fold values were used for the minimum and maximum limits of the distribution, respectively. The duration of the short term movements were derived from the questionnaire in which all hunters reported trips of one to two days and observations from the GPS study in which all 8 dogs stayed less than one day in the community visited. The frequency of permanent movements was estimated from questionnaire data and observations of permanent movements during September 2013 and September 2014. Dog owners reported that 18% (6/33) of the NPA dogs originated from a different community within the NPA, which resulted in an estimated probability of permanent movements of 1.64*10^−4^ per dog per day assuming an average dog life of three years. In addition, owners of 6% (3/49) of the dogs were observed to have permanently moved between NPA communities during the year, resulting in a probability of daily movements of 1.64*10^−4^ per dog. The sum (3.3*10^−4^) was selected as the mode of the beta-pert distributed daily probability of permanent movements per dog, with 0.5 and 2-fold values for minimum and maximum. Finally, the destination community for both permanent and short term movements was observed to be more frequently a neighbouring community than any other; consequently a neighbouring community as the destination was assumed to be twice as likely as for any other community in the NPA.

The median distance between each household and its closest neighbour–used in the model to truncate the distance kernel for the dog confinement control strategy–were estimated for each community separately using the coordinates of all households per community. Household coordinates were derived from Google Earth (http://earth.google.com/) where placemarks were set on all private dwellings, identified with the help of community maps. The distances between each household and its closest neighbour were calculated and the mean per community implemented in the model as a fixed value parameter.

### Sensitivity analysis

In the first step of the sensitivity analysis (SA), all model parameters used for six different control strategies were tested using the strategy’s default values: a) vaccination with 70% coverage either pre-emptively (PV) or reactive (RV); b) culling of dogs contacted by a rabid dog (CC) or reactive culling (RC) with culling levels of 80 and 50%, respectively; c) dog confinement plus movement bans between communities (MB) with 80% and 90% compliance, respectively; and d) a non-intervention strategy (NI). For all of these strategies, including NI, culling of dogs detected rabid (DC) was applied. The number of index dogs was set to 1 and randomly selected in the region. For each of the 12 combinations of the two regions (NPA and Elcho Island) and six control strategies, 1000 model repetitions were simulated.

For stochastic parameters, the mode or mean (for beta-pert distributed and uniform distributed parameters, respectively) was allowed to range between ±25% around the default value while the difference between the minimum and maximum values was kept fixed (no variation of the distributions’ shape; S3 Table, S1 Fig). Deterministic parameters were allowed to vary ±25% around the default value. Variation of 25% has been chosen to allow enough variation for parameters with wide distributions (e.g. the infectious period) and avoid too large distinction between the lower and upper limit of narrow distributed parameters (e.g. the rabies transmission probability given a bite). The distance kernel was tested using three different shapes representing a minimal kernel and increased probabilities for short and long distance contacts, respectively (S2 Fig). The influence of the index community within the NPA region was investigated by defining one of the five communities hosting the index dog. To explore the sensitivity of the model on the weighting matrix to choose the destination community for between-community movements, an alternative matrix was tested beside the default with equal chance for all communities to be selected as the destination.

The values of all parameters tested were randomly selected from the described ranges so that for each simulation, an individual set of parameter value combination was chosen. For each of the 12 region-strategy combinations, linear multivariable regression analysis was modelled with the outbreak duration and–where applicable–outbreak size as the response variable and the parameter values as explanatory variables. For the stochastic parameters the mode (beta-pert distribution) and mean (uniform distribution) values were modelled as explanatory variable values. Correlations between the parameter values were explored using Kendall’s tau correlation, Chi-Square and Wilcoxon Rank Sum test for two continuous, two categorical and a continuous and categorical parameter, respectively. Because the assumption of a normally distributed response variable was not always met (S3 Fig), logistic regression following the same principle was modelled defining an outbreak with a duration or size above the median as 1 and as 0 otherwise. Based on both the linear and logistic regression analyses, parameters were defined to be highly (statistically significant p-values < 0.05 in ≥ ¾ of all tested regression models), low (statistically significant p-values < 0.05 in < ¼ of all tested regression models) or moderate (otherwise) sensitive to the outbreak duration and size. Additionally, scatter plots of the outbreak duration and size over the range of each parameter value were visually analysed and correlations were calculated between outbreak duration and size and parameter values with continuous scale. A correlation of >|0.1| was considered as a threshold to distinguish between sensitive and non-sensitive parameters.

Parameters found to be highly sensitive in either of the regression analyses during the first step of the SA were further explored in a second step to identify the influence of their mode or mean (default, large, small) and shape (default, narrow, wide) on the model’s outcome. For the beta-pert and uniform distributed parameters nine combinations per parameter with the three values of mode or mean (default and ±10%), and three values of difference to the minimum and maximum (default and ±10%) of the distribution were defined (S4A–S4C Fig). For the vaccine efficacy parameter, the variation of 10% had to be reduced to 4%, which was the highest variability still ensuring a maximal value < 1, a requirement for probabilities (S4D Fig). For each parameter, 1000 repetitions were simulated for the same 12 region-strategy combinations described in step 1 of the SA (in case of the vaccine efficacy only the scenarios for RV and PV), where one of the nine options was randomly chosen while all other parameters in the model were kept at their default value. The distance kernel, defined by the three variables α, β and β_se_, was explored by varying the three variables around a default value ±50%, resulting in 27 combinations (S5 Fig). Six thousand repetitions were simulated for all 12 region-strategy combinations with a randomly selected distance kernel out of the 27 options while all other model parameters were kept at their default values. The outcome was analysed visually comparing boxplots of the outbreak duration and number of rabid dogs.

### Coefficient of variation

A critical question for stochastic models is always, how many repetitions are required to sufficiently reflect the variability of the model? The coefficient of variation (CV = standard deviation/mean) of model outputs’ mean has been proposed as a measurement to determine the critical number of repetitions required [30,31]. The CV of the estimated mean of outcome of interest (e.g. outbreak size or duration) over *n* model simulations is expected to approach 0 for infinite sample sizes *n* and a threshold of the CV of 15% has proposed to predict outputs with acceptable precision [30]. We used the same approach, but reduced the CV threshold value to 3%.

### Illustrative simulation outcomes

To demonstrate the model’s functionality for the different control strategies, 1000 outbreaks were simulated for the default strategies: 1. reactive vaccination with 70% coverage at the household level (RV); 2. reactive culling with 50% of the dogs culled in affected communities (RC); and 3. dog confinement between and within communities with 90 and 80% compliance, respectively (MB). Culling of dogs detected rabid (DC) was applied for all of these strategies. The model was simulated in both regions, NPA and Elcho Island, separately. As outcome measures, the epidemic duration and size, i.e. the number of rabid dog and the number of dead dogs (including rabid and culled dogs) were calculated and visually compared between the different scenarios.

## Results

### Outbreak size and duration of example simulations

**Fig 4:**
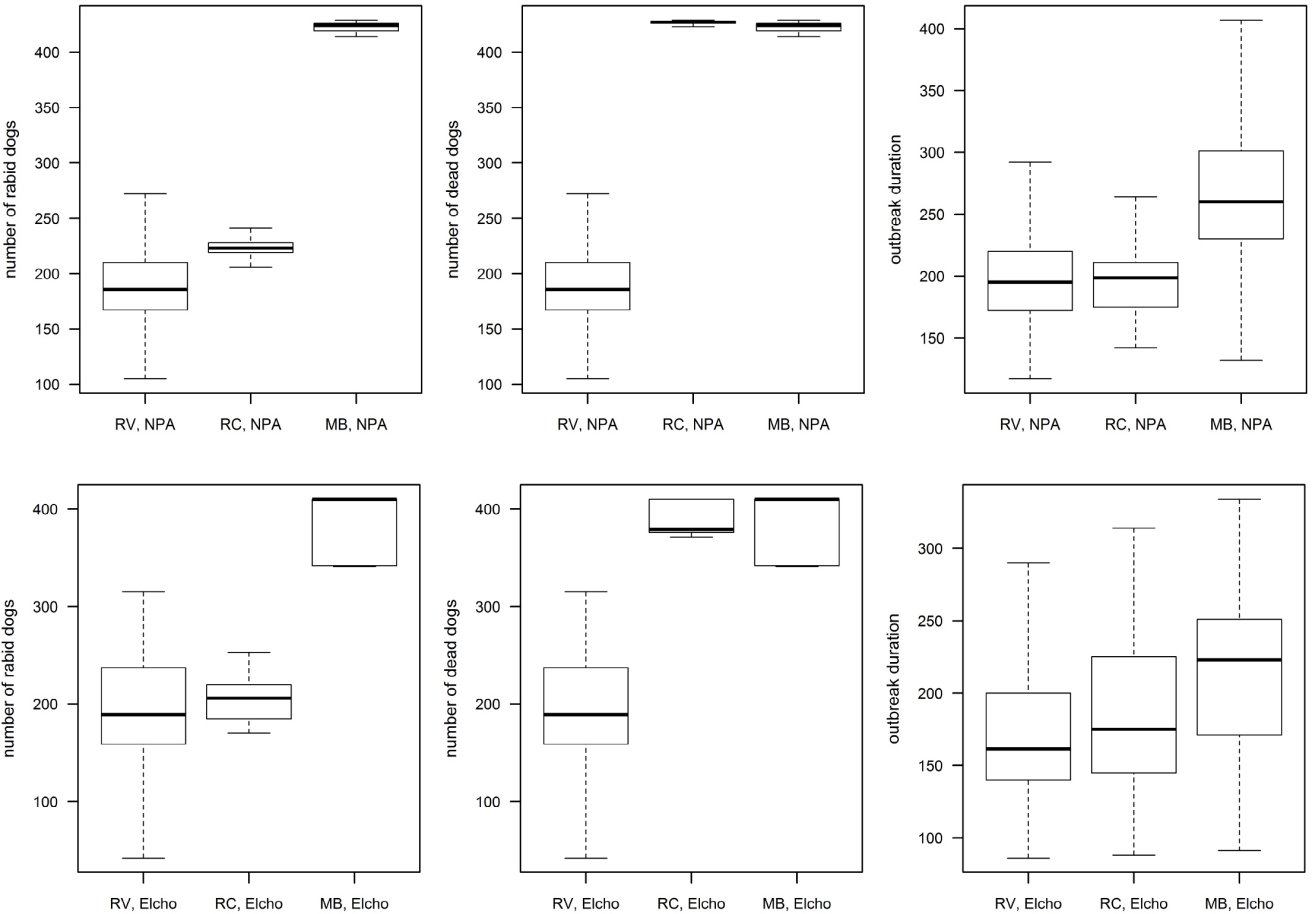
Outbreak results for three example control strategies based in 1000 repetitions per scenario. Three control strategies (RV = reactive vaccination, RC = reactive culling, MB = movement bans) in two regions (Elcho = Elcho Island, NPA = Northern Peninsula Area) were simulated. Number of rabid dogs, dead dogs (i.e. due to rabies or culling) and outbreak duration per scenario are represented for the NPA (upper line) and for Elcho Island (lower line). The box represents the interquartile range (IQR), the horizontal line in the box the median and the whiskers extend to the most extreme data point which is no more than 1.5 times IQR from the box. Outliers are not shown.

Two measures of outbreak size are the cumulative number of rabid dogs and cumulative number of dead dogs (due to rabies plus culled). The outbreak duration is defined as the number of days from the introduction of the latently infected index dog until the death of the last infectious dog. The simulation model resulted in plausible results comparing outputs for the three different default control strategies (Fig 4). Rabies spreads through the communities in a wave pattern and, depending on the control strategy, can kill the entire dog population (S6 Fig illustrates an example epidemic curve). The reactive culling (RC) strategy reduces the number of rabid dogs; however the number of dead dogs is only slightly less than the total dog population. For the reactive vaccination (RV) strategy the number of rabid dogs (equal to the number of dead dogs) showed higher variability among the 1000 model simulations compared to RC, but in both regions, the median of RV was lower than for RC. Obviously, vaccination saves the dogs from death in contrast to culling strategies. The movement ban (MB) strategy showed a slight decrease of the outbreak size in the region of Elcho Island whereas no effect was observed in the NPA. However, it was found to be the strategy with the longest durations of outbreaks, demonstrating that movement bans (if not 100% compliant) only slow the speed of spread rather than reducing its size. The reduction in the number of movements between communities for MB was obvious, decreasing from a median of 52 (RV) and 46 (RC) to 19 (S7 Fig). Overall, outbreak duration ranged from 1−20 months (median 6.7 months) and was more homogenous between interventions than the outbreak size (Fig 4). Outbreaks lasting for one month did not spread beyond the index dog. For the RV strategy, the vaccination coverage was set at 70% of the households, producing dog level vaccination coverage of 59−75% and 56−76% for the NPA and Elcho Island region, respectively.

The control strategy with the largest number of simulations required (n = 490) to capture the variability in the model’s output with the defined CV threshold of 3% was found to be the MB strategy in the NPA (S8 Fig).

### Basic and effective reproductive ratios R_0_ and R_t_

**Fig 5:**
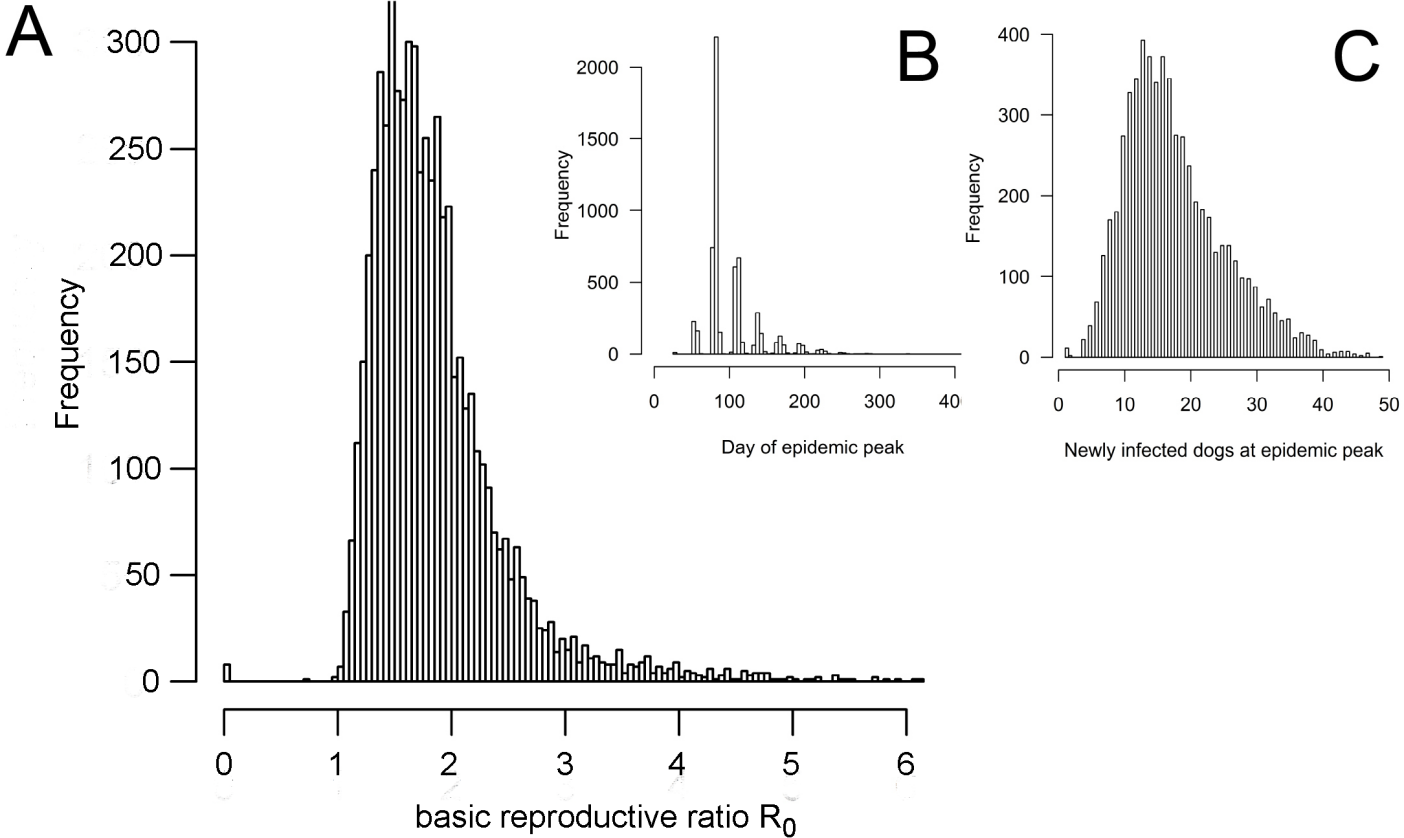
Summary of model outputs for 6000 repetitions including results from all three control strategies in both regions (1000 repetitions for each scenario). (A) Frequency distribution of the basic reproductive ratio R_0_, (B) frequency distribution of the day of the epidemic peak and (C) number of newly infected dogs at the day of the epidemic peak.

The number of secondary cases was reported for every rabid dog over the duration of the outbreak. From these records, the basic reproductive ratio R_0_ was calculated and defined as the mean number of secondary cases for dogs becoming infectious within the first phase–i.e. up to its peak–of an epidemic. The peak of the epidemic is defined as the day with the highest number of newly infectious animals over the entire outbreak. R_0_ ranged from 0−6.1 (median 1.8) for RV, 0−6.1 (median 1.7) for RC and 0−5.7 (median 1.7) for MB (S9 Fig) with an overall median of 1.7 (Fig 5A). The epidemic peak was reached on average after 93 days (Fig 5B) with a mean of 17 newly infected dogs (Fig 5C). The number of secondary cases derived from each index dog was highly variable and ranged from 0 to 79 (median of 25) for NPA and 4 to 106 (40) for Elcho Island. Over the duration of the outbreak, the effective reproductive ratio R_t_ and the number of dogs in the susceptible population decreased in a wave pattern (S10 Fig). The value of 1 for mean R_t_ is reached during the second or third wave. This reflects that R_t_ depends on the dogs remaining in the population and finally the outbreak dies out because there are no susceptible dogs left that are close enough to the infectious dogs.

### Sensitivity analysis

The simulation model outputs were highly sensitive to seven parameters: incubation period (G1 in S4 Table), transmission probability given a bite (G8), distance kernel (G5), bite probability given a contact between dogs of different households (G7), vaccine efficacy (V5), index community (G12) and delay in starting the control strategy of movement restrictions between communities (B2). The same sensitive parameters, in addition to the detection delay of the first clinical case, were also identified via correlation tests, with the exception of B2. These outcomes were also confirmed by scatterplots, which express particularly dependencies between the outbreak duration and the incubation period, distance kernel and index community (S11 Fig). Significant correlations between parameters included in the regression analyses were only observed between categorical and continuous parameters where 2.1−12.1% (mean 5.4%) of all parameter combinations resulted in Wilcoxon Rank Sum test p-values <0.05.

The influence of this subset of parameters was further explored in step 2 of the SA, with the exception of the index community and the delay in commencing movement restrictions because these two parameters directly relate to incursion and intervention scenarios. For all parameters, except the distance kernel, it was found that both the mode and mean (for beta-pert and uniform distributions, respectively) and the shapes influence outbreak duration and number of rabid dogs (S12 Fig). The mean and mode were found to have a greater impact, particularly for the incubation period and rabies transmission probability. For the distance kernel, the regression coefficient β was most influential on both the number of rabid dogs and the outbreak duration, followed by β_se_ (standard error of β), particularly for the Elcho Island region (S13 Fig). Outputs were less sensitive to the intercept α.

## Discussion

The model described herein provides insights into short-term rabies epidemics occurring within a small spatial extent in previously rabies-free regions. This is of crucial value for contingency planning in areas where rabies is exotic and the model fills a gap in the published literature on rabies models. The example of Bali, Indonesia demonstrates the impact of a rabies incursion on an under-prepared region [12]. Late detection of the disease, lack of surveillance strategies and an unsuccessful initial response (focused on dog culling) resulted in island-wide disease spread and high impacts on both dog and human populations [7,12,32]. Another example in Indonesia–comparable to communities in northern Australia with a high density of free-roaming dogs and limited veterinary health services − is the island of Flores in East Nusa Tengarra province [14,33,34]. There, one or more latently infected dogs were introduced, developed clinical rabies and transmitted the disease to local dogs. The disease consequently spread throughout island with a considerable impact on dogs and humans. This is another example of a rabies invasion in a new area with very severe impact.

Another novel aspect of the current model is the inclusion of individual susceptible and rabid dogs modelled within a continuous spatial dimension, an approach previously used to simulate highly infectious diseases of livestock (e.g. foot-and-mouth-disease [35,36]) but not rabies. To date, published dog rabies models have been based on mathematical differential equations [5,24,25,27] or spatially explicit models simulating the spread of rabies within grids [7]. Our approach has several advantages, including stochasticity to capture epidemic variability, incorporation of detailed population structure to better represent real target regions of interest, and detailed spatio-temporal model outputs.

By simulating three default control measures–vaccination, culling and dog confinement–our model produced plausible results, suggesting it adequately captures how an epidemic of an infectious disease with a relatively long incubation period would develop in a previously uninfected population. The disease spread temporally in wave-like patterns, peaking on average about three months after an incursion. The average R_0_ that was estimated from the model outputs was 1.7, consistent with previous estimates of rabies spread in endemic countries [16,37] and estimated from the Bali outbreak [7].

For the calculation of R_0_ we considered rabid dogs up to the peak of the epidemic, while peak has been defined as the day during the epidemic with the highest number of newly rabies infectious animals. It has been demonstrated that both, clustering within the susceptible population and high number of repetitive contacts among individuals in the population–thus a non-random mixing situation–affects the dynamics of disease spread via depletion of susceptible population within a cluster and therefore also affects R_0_ [38]. A re-evaluation of R_0_ of a H1N1 infection revealed that it was overestimated during the early stage of the outbreak when only cases within a cluster has been considered rather than the population-wide epidemic [39]. In our model, random mixing of infectious and susceptible animals has to be rejected as rabies transmission depends on the distance between infectious and susceptible animals. However, neither fixed repetitive contacts (as in network based models) nor significant clusters within communities are included in the model. The only functionally relevant cluster structure is present in the region of the NPA with the five communities building distinct clusters. We respected the cluster structure in the R_0_ calculation by not only considering cases to the peak within the first cluster, i.e. the community of the index dog, but all cases occurring before to the epidemic peak within the total population at risk in the respective region.

As illustrative examples we simulated the most often discussed and applied control strategies for dog rabies, namely vaccination and culling of dogs as a reactive action, as well as dog movement restrictions. According to the model, the only beneficial measure (based on outbreak size) is vaccination. This is consistent with a range of studies in regions where a rabies incursion has been observed: culling as a single control measure was unsuccessful [7,12,14,33], whereas vaccination was demonstrated to be a successful strategy to control recent rabies invasions [7,10,32,40]. However, success of vaccination campaigns obviously depends on the vaccination coverage, as the example of a unsuccessful rabies control via low level vaccination coverage demonstrated in Flores [34]. Also, culling can lead to an eradication of timely detected outbreaks, as for example in region in Bhutan [9], however might be impractical because of non-acceptance, depending on culture and religion of the community. In Australian Indigenous communities, culling of dogs is unlikely to be culturally acceptable. According to our model, movement bans as a single strategy does not seem to be sufficient to reduce rabies spread from one community to another nor within a community–at least for the simulated dog owner compliance (80–90%) that was simulated here. Movement bans would culturally also be difficult to implement as travel with dogs between communities and regions is common. These results are similar for both regions, the NPA and Elcho Island. Within the NPA, rabies epidemics were able to sustain after incursion in each the five communities, as it does for the one community on Elcho Island, identifying the here considered regions and communities equally susceptible. Targeting surveillance should therefore be based on information revealed by risk assessment pathways exploring high risk regions for a rabies incursion. Further detailed simulations exploring combinations of response measures and their threshold values for effectiveness–including the effect of surveillance intensity [40]–is warranted as future research.

The non-intervention strategy was implemented in the model and applied during the sensitivity analysis as a “baseline” to quantify the benefit of other control strategies, although it will most certainly never be observed in the field. Keeping in mind the assumption of a closed dog population with no influx of new dogs (neither birth nor immigration), the high densities of dogs that roam freely in the modelled community and the assumption of an infectious period of up to 12 days combined with a fully naïve population, it is expected that the epidemic will eventually kill the modelled dog population, in the case of no intervention applied.

Model outputs were sensitive to the assumed distance kernel for rabies transmission between dogs from different households. This highlights that the distance kernels should be empirically developed for the particular regions in which the model is to be applied. In our case, the kernel was estimated from roaming dog data collected within the actual study regions [18]. Model outputs (size and duration of the epidemics) were also sensitive to the assumed rabies incubation period and probability of rabies transmission given a bite. These parameters were derived from previously published field observations in Africa (S1 Table), and are likely applicable to many situations, as is the vaccine efficacy parameter. For the probability of bite given a contact between dogs, no specific published data could be found. We assumed this parameter to be >50% due to the aggressiveness that rabies can cause [41], but we also included a large range of uncertainty (60–80%).

Critical parameter values (as for contact rates and the population structure) in this model are informed by data collected in the field. This guarantees the fit of the model to the intended environment of application, although limitations still do occur. Data informing the distance kernel were based on the roaming behaviour of healthy dogs [18]. A rabid dog might change its normal roaming behaviour, as for example reported for rabid racoons in New Jersey (USA) which moved over significantly larger distances than healthy racoons [42]. Apparently the effect of rabies on the roaming behaviour of domestic dogs has not been reported, but considering the observed changes in racoon behaviour, our model might underestimate the spread of rabies. When comparing contact distances observed in our study (healthy dogs, mean distance = 103 meters) that inform this model with the published spatial infection kernel of contacts between rabid dogs and resulting infection (mean = 880 meters, [37]), rabid dogs might roam up to 8.5 times further than healthy dogs. This again indicates that in our model, although long distance movements have been included otherwise, transmission events might be underestimated. Studies on the nature of roaming, contact rates and biting rates of dogs in rabies-endemic studies–comparing these between infected and apparently uninfected communities in the same environment–are needed to address this gap in our knowledge and to more realistically parameterise models of rabies incursion.

In the model, we have focused on the domestic dog population and ignored possible spread to wildlife (in this region, wild dogs and dingoes) because we were interested in the initial epidemic behaviour of a rabies incursion − and hence its impact on domestic dog health and by implication, human health. We assumed a closed dog population without introductions, births and natural deaths, again because our focus was on exploring initial disease response actions rather than the design of long-term rabies control programs.

Although a critical issue for simulation model development, we have not yet validated the model using real outbreak data. Since Australia is historically rabies-free, a direct validation of the model in the region where it is intended for use is impossible, until an actual rabies incursion occurs. However, outbreak data from regions with similar dog population characteristics and recent rabies epidemics might be used, for example the island of Bali [12] or parts of Bhutan [9,10]. Validation of the model using data from a rabies outbreak in domestic dogs in Bhutan in 2008 is currently being planned.

Global control of rabies is a declared goal of the World Health Organization and the Global Alliance for Rabies Control [43]. Within endemic, mostly developing countries, knowledge of effective control strategies is advanced and the challenge is to transfer this knowledge into successful actions [44]. Effectiveness of control strategies to prevent rabies from establishing in previously free regions–as is currently occurring in several areas with large free-roaming dog populations and limited public health services–has not received the same attention. The model described here is a tool to generate such information for remote northern Australia; and it is flexible enough to be adapted to other regions.

## Supporting Information

S1 Fig Variation in the input values of five parameters as an example for all continuous, stochastic parameters included in the first step of the sensitivity analysis. Only the mode and mean for beta-pert and uniform distributed parameters, respectively, changed, whereas the shape of the parameter remained constant. The default value and lower and upper limits (±25% of the default value) are presented. (A) Beta-pert distributed parameters: incubation period (left), infection period (middle) and rabies transmission probability given a bite (right). (B) Uniform-distributed parameters: detection delay of rabid dogs for the first (left) and consecutive cases (right).
(PDF)

S2 Fig Variation in the input values of the distance kernel in the first step of the sensitivity analysis. The kernel with increased probabilities of short distance contacts is the default kernel (middle, a contact being defined as being within 20 meters within 1 minute), the minimal kernel (left) was calculated using a contact definition of being within 10 meters within 1 minute and for the kernel with increased probabilities of long distance contacts (right) using a contact definition of being within 28 meters within 1.5 minutes. hh = household, prob = probability
(PDF)

S3 Fig Histograms of the number of dead dogs (top row), rabid dogs (middle) and outbreak duration (bottom) for the 1000 repetitions per control strategy. Particularly for the number of dead and rabid dogs the assumption of normally distributed outcomes is rejected. (A) Results for NPA; (B) results for Elcho Island.
(PDF)

S4 Fig Variation of the input values of the four continuous parameters explored in the second step of the sensitivity analysis. Both the mode or mean and the shape of the parameters were varied ±10% around their default values (for the vaccine efficacy ±4%). The variation in the shape is defined as varying the difference between the mode or mean to the upper and lower limit of the beta-pert or uniform distribution, respectively. The continuous line represents the default value of the shape, the dashed line the narrow shape and the dotted line the wide shape. (A) incubation period, (B) rabies transmission probability given a bite, (C) probability of being bitten given a contact between two dogs from different households (hh) and (D) vaccine efficacy.
(PDF)

S5 Fig Variation in the input values of the distance kernel explored in the second step of the sensitivity analysis (SA). All three variables (α, β, β_se_; see main text for further details) defining the distance kernel were varied ±50% around a medium value, resulting in a total of 27 distinct distance kernels. The medium value of α was defined as the mean of the large (default kernel) and low (minimal kernel) intercept values of the kernels used in step 1 of the SA and the medium value of β was set at the mean coefficient value of all kernels used in step 1. The three values of β_se_ were defined as a relationship to β, with β_se_/β varying ±50% around the medium, which is calculated from the three kernels used in step 1 of the SA. The coefficient (β) is decreasing from the top (-50% of default value) to the bottom (+50% of default value). The standard error β_se_ of β is decreasing from the left (+50% of default value) to the right (-50% of default value). The variation in the intercept α is presented in (A) large intercept (+50% of medium values), (B) medium intercept (medium value) and (C) small intercept (-50% of medium values). To facilitate the comparison between the different kernels the probabilities for a daily contact between two dogs living 300 meters apart from each other are highlighted by the grey lines.
(PDF)

S6 Fig Epidemic curve of one illustrative epidemic in the NPA. Number of newly exposed (latently infected, blue line), new rabid (red line) and new dead (green line) dogs per day during the outbreak under the MB strategy. The wave pattern can be observed.
(PNG)

S7 Fig Total number of dog movements between the communities in the NPA during 1000 repetitions per control strategy (RV, RC and MB). The boxes in the boxplots represent the interquartile range (IQR), the horizontal line in the box the median and the whiskers extend to the most extreme data point which is no more than 1.5 times IQR from the box. Outliers are not presented.
(PDF)

S8 Fig Coefficient of variation (CV) of the estimated mean of the number of rabid dogs (red line) and dead dogs (green line) and outbreak duration (black line) over 1 to 1000 repetitions per control strategy and region. The horizontal line depicts the CV threshold of 3% and the vertical lines indicate after how many repetitions this threshold has been reached for the three measures rabid and dead dogs and outbreak duration.
(TIFF)

S9 Fig Frequency distributions of the basic reproductive ratio R_0_ per control strategy in the two regions. Demonstrating the independency of R_0_ on these control strategies which is expected for the early phase of the outbreaks.
(PDF)

S10 Fig Number of secondary cases for all rabid dogs in the model during the epidemic (black dots) and mean value of secondary cases for each day (red line). The time value (x-axis) for each dog was set at the day of the start of the infectious period. The green line represents the mean number of susceptible dogs in the population during the epidemic for 1000 repetitions per scenario. The blue horizontal line is drawn at 1, the critical value of R_0_. Over the duration of the outbreak, the effective reproductive ratio R_t_ is decreasing, which is caused by control measures applied but also by the drop of the number of dogs in the susceptible population.
(PDF)

S11 Fig Scatterplots depicting the dependency of the outbreak duration on the parameter values during the first step of the sensitivity analysis. Boxplots are used to present the dependency from categorical or continuous parameters with 5 or less distinct values. The boxes in the boxplots represent the interquartile range (IQR), the horizontal line in the box the median and the whiskers extend to the most extreme data point which is no more than 1.5 times IQR from the box. The three values of the distance kernel (dist_kernel) refer to the minimal distance kernel (min), increased short distance (shortD) and long distance probability (longD) of S2 Fig.
(PDF)

S12 Fig Dependency of the model outcome (number of rabid dogs and outbreak duration) on the variation of the mode/mean and shape of the parameters selected for sensitivity analysis step 2. The x-axis represents the value of the mode (A/B, E/F) or mean (C/D, G) of the parameter and the terms “default”, “narrow” and “wide” describe the shape; see main text for further details. The boxes in the boxplots represent the interquartile range (IQR), the horizontal line in the box the median and the whiskers extend to the most extreme data point which is no more than 1.5 times IQR from the box. (A) Dependency of the number of rabid dogs on the incubation period; (B) dependency of the outbreak duration on the incubation period; (C) dependency of the number of rabid dogs on the probability of being bitten given a contact between dogs of different households; (D) dependency of the outbreak duration on the probability of being bitten given a contact between dogs of different households; (E) dependency of the number of rabid dogs on the probability of rabies transmission given a bite; (F) dependency of the outbreak duration on the probability of rabies transmission given a bite; and (G) dependency of the number of rabid dogs (upper half) and outbreak duration (lower half) on the vaccine efficacy.
(PDF)

S13 Fig Dependency of the model outcome (number of rabid dogs and outbreak duration) on the variation of the three parameters defining the distance kernel during the sensitivity analysis step 2. The three parameters are the intercept (*IC*), coefficient (*coef*) and standard error of the coefficient (*se*) and their distinct values on the x-axis were: *IC* 0.439 (A), 0.878 (B) and 1.318 (C); *coef*: -0.0174 (A), -0.0116 (B), -0.0058 (C); and *se* as “default” (def), “narrow” (nar) or “wide” (wide). See main text for further details. The boxes in the boxplots represent the interquartile range (IQR), the horizontal line in the box the median and the whiskers extend to the most extreme data point which is no more than 1.5 times IQR from the box. (A) Dependency of the number of rabid dogs on the three parameters defining the distance kernel for NPA; (B) dependency of the number of rabid dogs on the three parameters defining the distance kernel for Elcho Island; (C) dependency of the outbreak duration on the three parameters defining the distance kernel for the NPA; and (D) dependency of the outbreak duration on the three parameters defining the distance kernel for Elcho Island.
(PDF)

S1 Table Model parameters. Name, description, default values and source of all parameters used in the rabies simulation model; stochastic values either follow a beta-pert distribution (Pert(Min,Mode,Max)) or a uniform distribution (Unif(Min,Max)).
(DOCX)

S2 Table Data collected via questionnaire in the Northern Peninsula Area. (XLSX)

S3 Table Parameters explored during the first step of the sensitivity analysis (SA) with their default value, and the lower and upper limits of variation during the SA. Parameters were tested in all possible scenarios, which were mostly all of the 12 (six control strategies in two regions) but not always (see column Tested scenarios).
(DOCX)

S4 Table Regression analyses results during the first step of the sensitivity analysis. (XLSX)

S5 Table Data collected during the GPS collar study in the Northern Peninsula Area, Australia, September 2013. (XLSX)

S6 Table Data collected during the GPS collar study on Elcho Island, Australia, November 2013. (XLSX)
