# Supplementary material for: Development of a Novel Rabies Simulation Model for Application in a Non-endemic Environment
Source: PLoS Negl Trop Dis. 2015 Jun 26;9(6):e0003876. doi: 10.1371/journal.pntd.0003876 (PMC4482682; doi:10.1371/journal.pntd.0003876)
Supplement: S1 Fig — Only the mode and mean for beta-pert and uniform distributed parameters, respectively, changed, whereas the shape of the parameter remained constant. The default value and lower and upper limits (±25% of the default value) are presented. (A) Beta-pert distributed parameters: incubation period (left), infection period (middle) and rabies transmission probability given a bite (right). (B) Uniform-distributed parameters: detection delay of rabid dogs for the first (left) and consecutive cases (right). (PDF) [file pntd.0003876.s001.pdf]

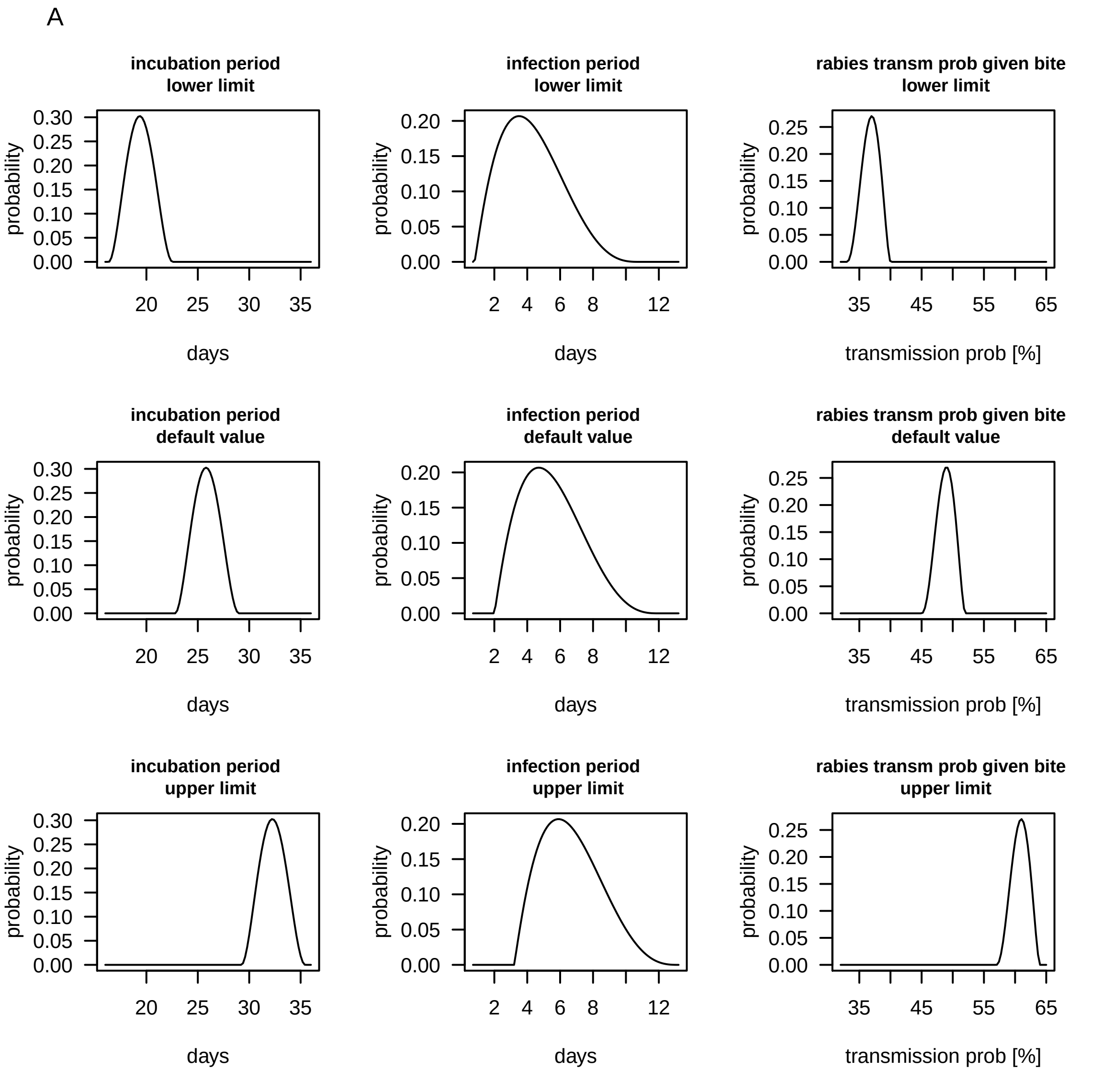
A
incubation period lower limit
infection period lower limit
rabies transm prob given bite lower limit
incubation period default value
infection period default value
rabies transm prob given bite default value
incubation period upper limit
infection period upper limit
rabies transm prob given bite upper limit
probability
days
transmission prob [%]
0.00 0.05 0.10 0.15 0.20 0.25 0.30
20 25 30 35
2 4 6 8 12
35 45 55 65

B

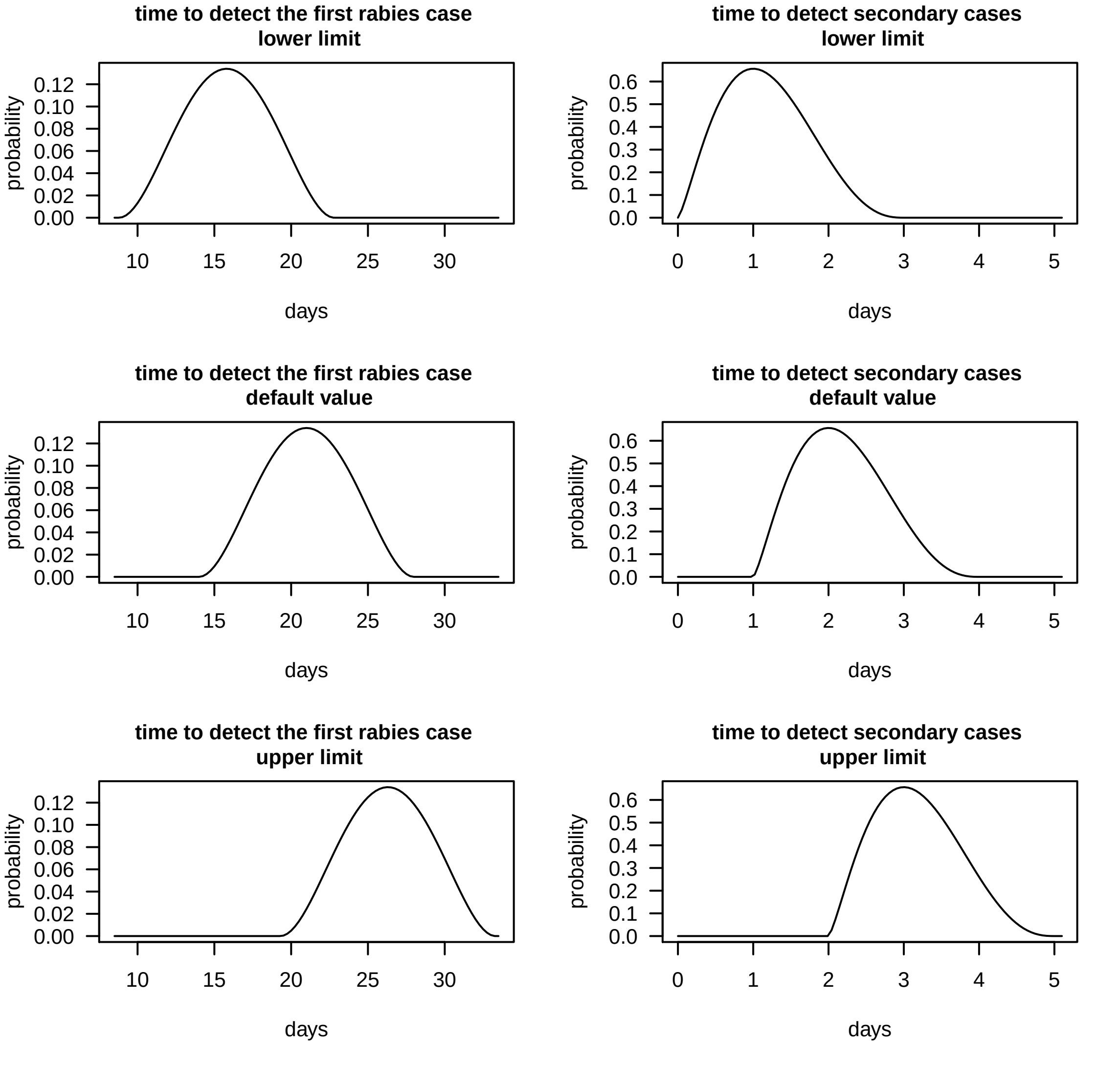
time to detect the first rabies case
lower limit
time to detect secondary cases
lower limit
time to detect the first rabies case
default value
time to detect secondary cases
default value
time to detect the first rabies case
upper limit
time to detect secondary cases
upper limit
probability
days
0.00
0.02
0.04
0.06
0.08
0.10
0.12
10
15
20
25
30
0.0
0.1
0.2
0.3
0.4
0.5
0.6
0
1
2
3
4
5
