# Supplementary figures and images for: Development of a Novel Rabies Simulation Model for Application in a Non-endemic Environment
Source: PLoS Negl Trop Dis. 2015 Jun 26;9(6):e0003876. doi: 10.1371/journal.pntd.0003876 (PMC4482682; doi:10.1371/journal.pntd.0003876)

**minimal distance kernel**

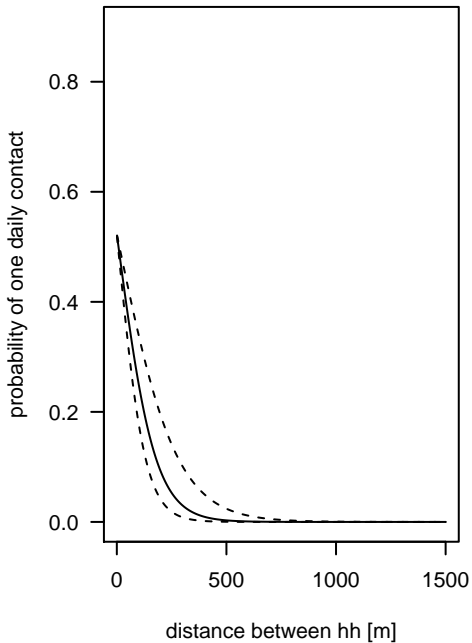

**increased short distance prob**

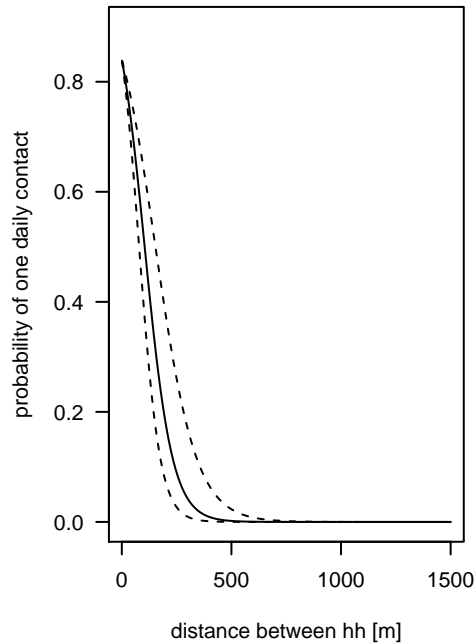

**increased long distance prob**

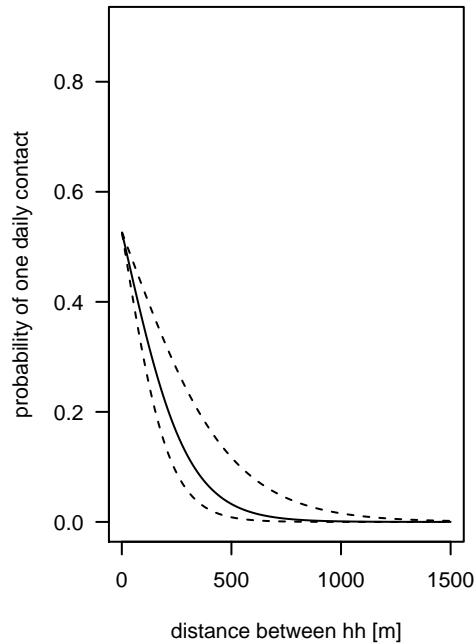

Supplement: S2 Fig — The kernel with increased probabilities of short distance contacts is the default kernel (middle, a contact being defined as being within 20 meters within 1 minute), the minimal kernel (left) was calculated using a contact definition of being within 10 meters within 1 minute and for the kernel with increased probabilities of long distance contacts (right) using a contact definition of being within 28 meters within 1.5 minutes. hh = household, prob = probability (PDF) [file pntd.0003876.s002.pdf]

A

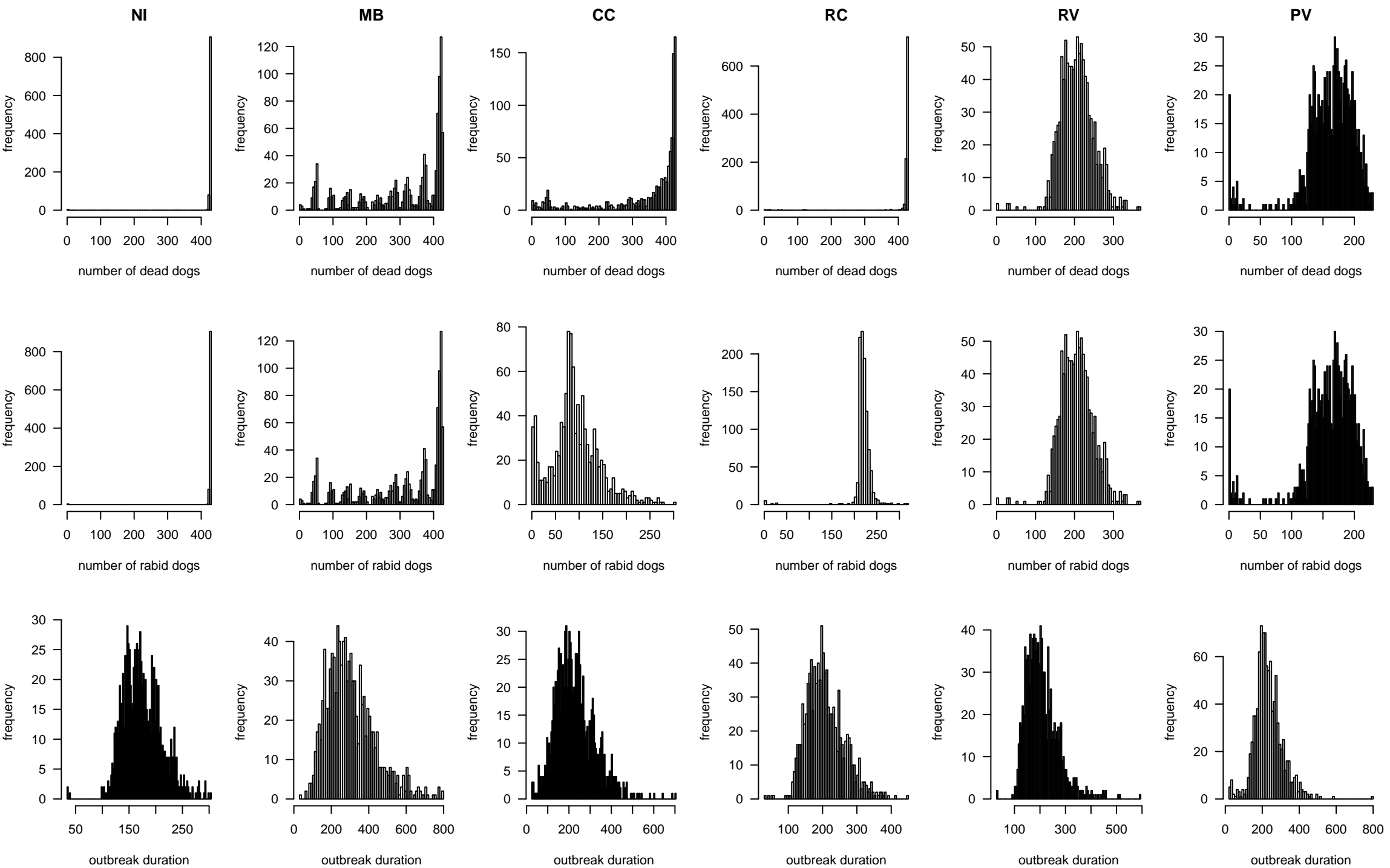

B

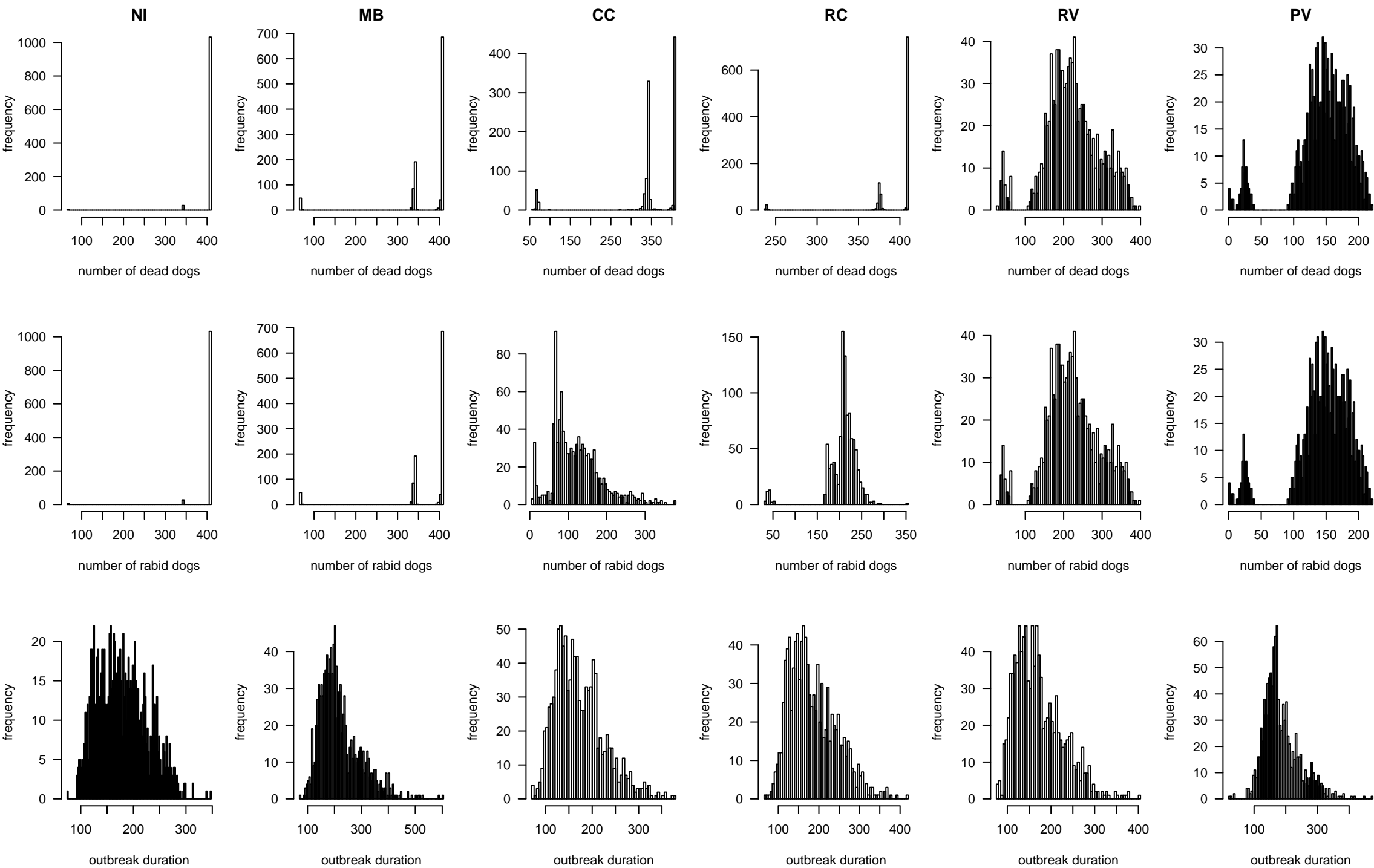

Supplement: S3 Fig — Particularly for the number of dead and rabid dogs the assumption of normally distributed outcomes is rejected. (A) Results for NPA; (B) results for Elcho Island. (PDF) [file pntd.0003876.s003.pdf]

A

## large intercept

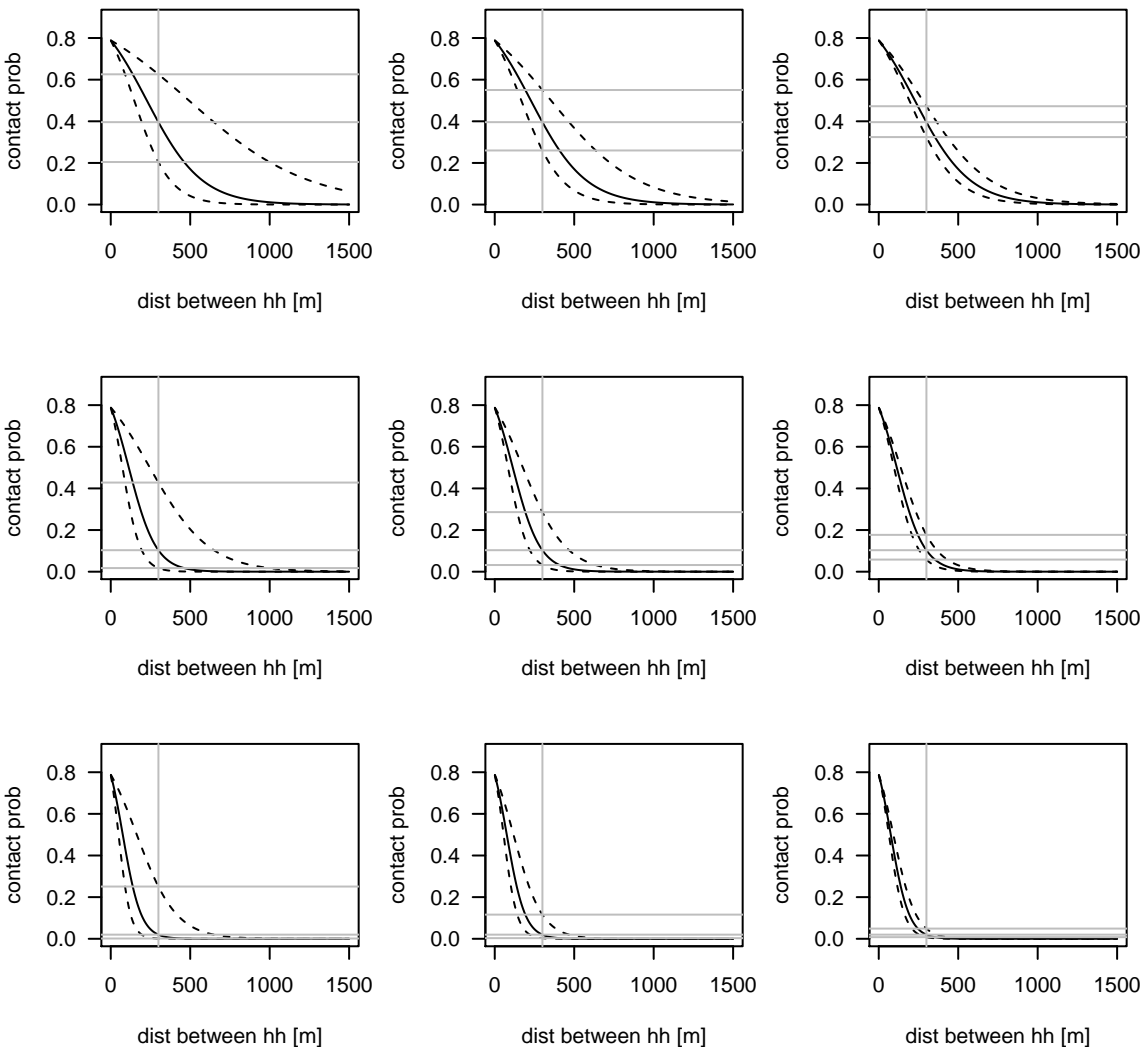

B

## medium intercept

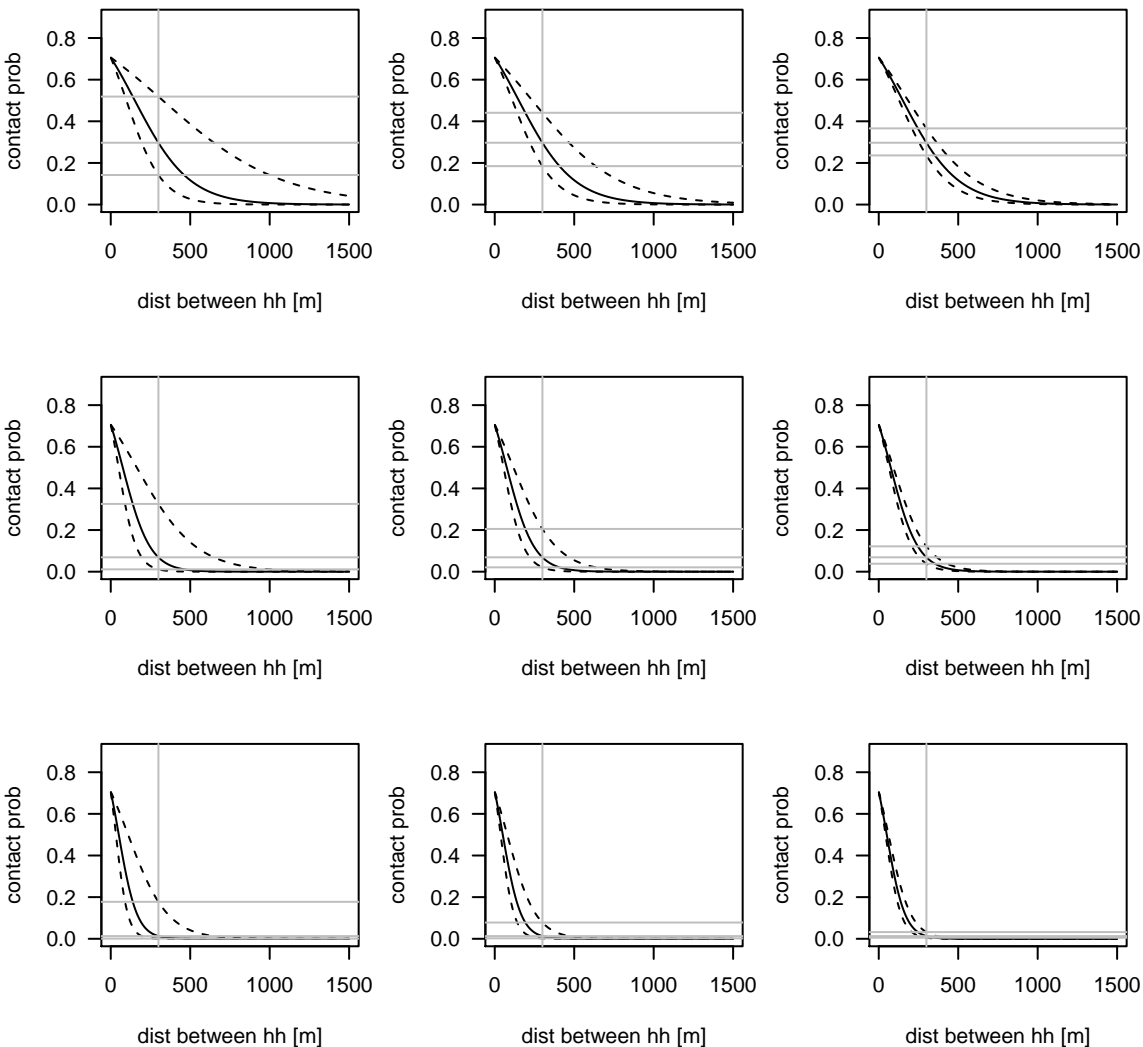

C

small intercept

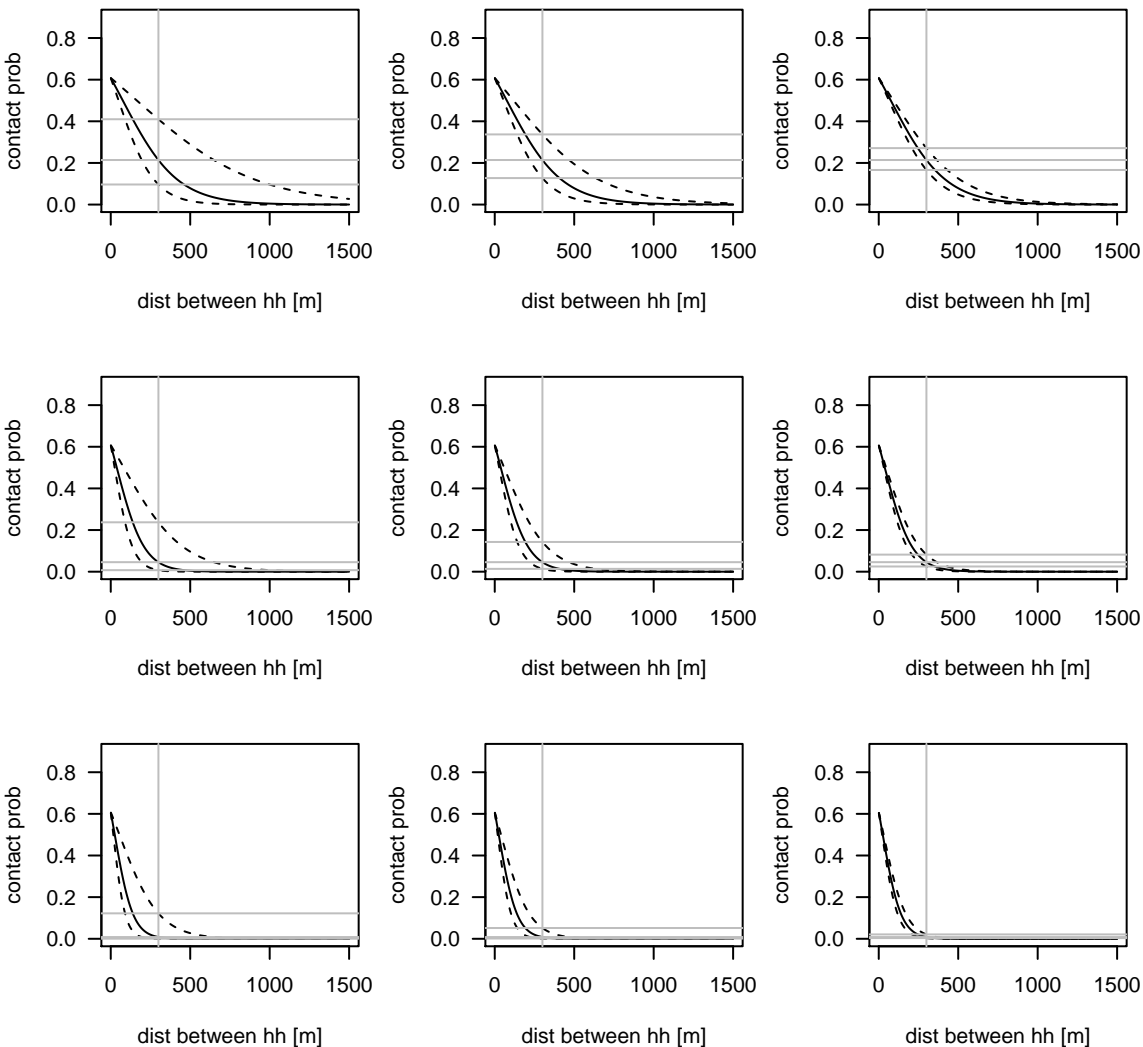

Supplement: S5 Fig — All three variables (α, β, βse; see main text for further details) defining the distance kernel were varied ±50% around a medium value, resulting in a total of 27 distinct distance kernels. The medium value of α was defined as the mean of the large (default kernel) and low (minimal kernel) intercept values of the kernels used in step 1 of the SA and the medium value of β was set at the mean coefficient value of all kernels used in step 1. The three values of βse were defined as a relationship to β, with βse/β varying ±50% around the medium, which is calculated from the three kernels used in step 1 of the SA. The coefficient (β) is decreasing from the top (-50% of default value) to the bottom (+50% of default value). The standard error βse of β is decreasing from the left (+50% of default value) to the right (-50% of default value). The variation in the intercept α is presented in (A) large intercept (+50% of medium values), (B) medium intercept (medium value) and (C) small intercept (-50% of medium values). To facilitate the comparison between the different kernels the probabilities for a daily contact between two dogs living 300 meters apart from each other are highlighted by the grey lines. (PDF) [file pntd.0003876.s005.pdf]

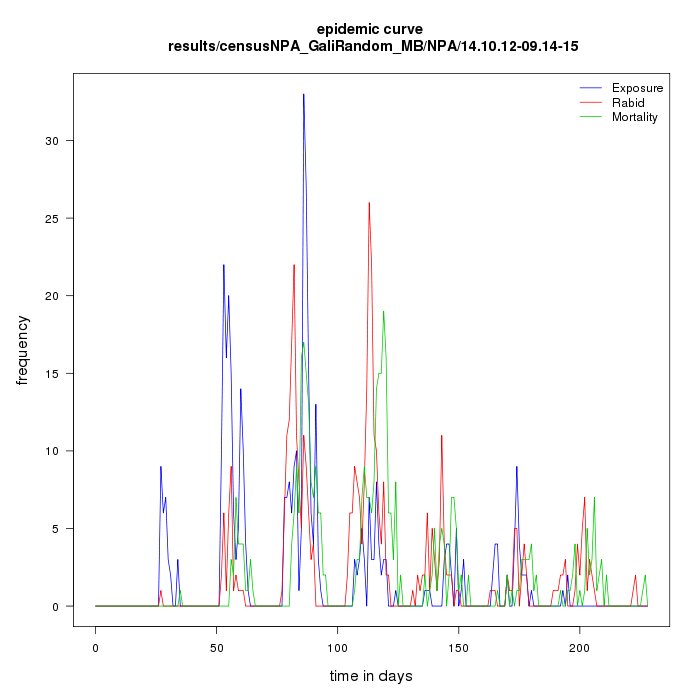

Supplement: S6 Fig — Number of newly exposed (latently infected, blue line), new rabid (red line) and new dead (green line) dogs per day during the outbreak under the MB strategy. The wave pattern can be observed. (PNG) [file pntd.0003876.s006.png]

# total number of moved dogs between communities in NPA

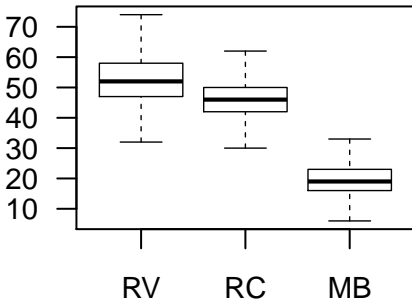

Supplement: S7 Fig — The boxes in the boxplots represent the interquartile range (IQR), the horizontal line in the box the median and the whiskers extend to the most extreme data point which is no more than 1.5 times IQR from the box. Outliers are not presented. (PDF) [file pntd.0003876.s007.pdf]

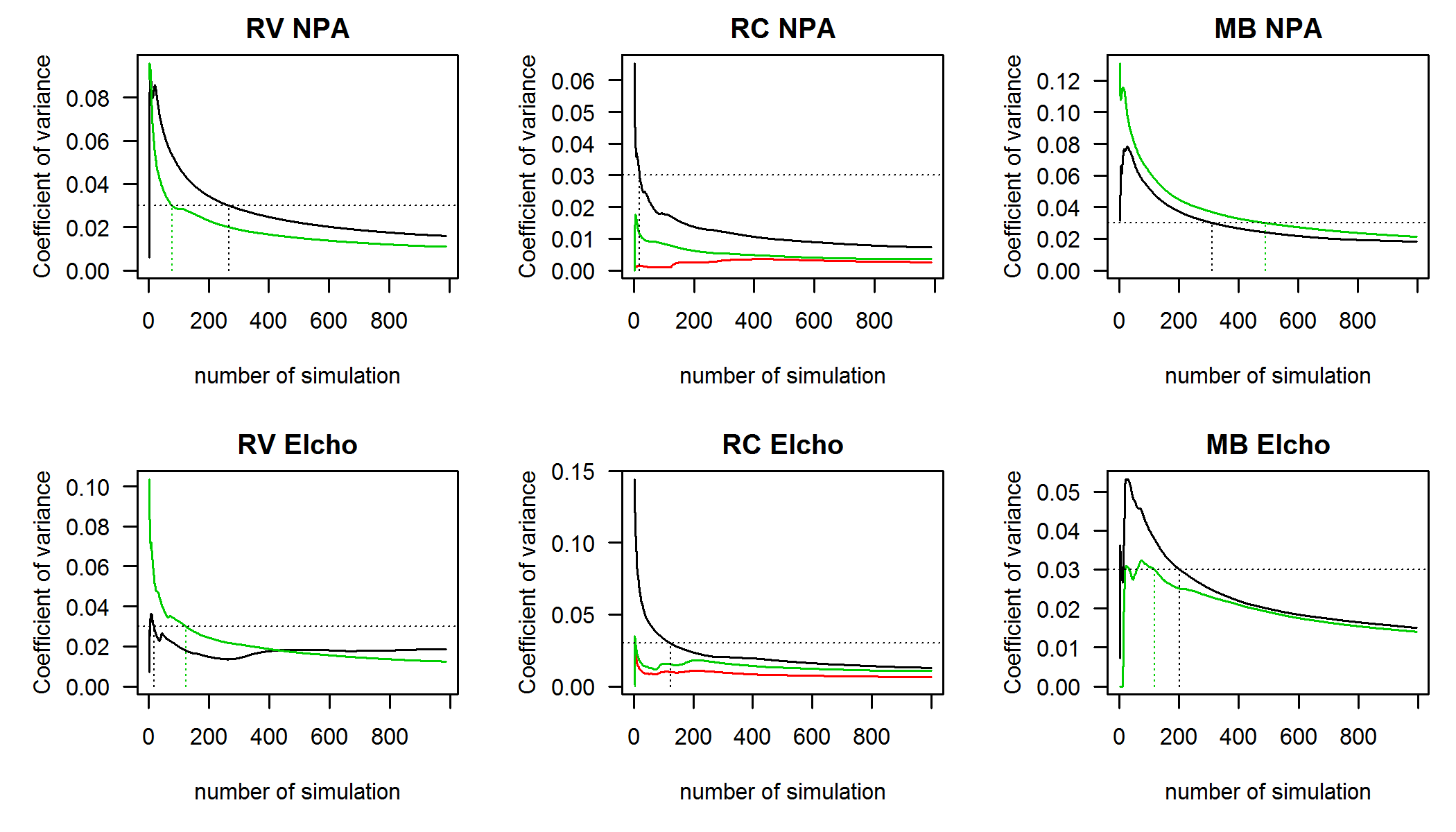

Supplement: S8 Fig — The horizontal line depicts the CV threshold of 3% and the vertical lines indicate after how many repetitions this threshold has been reached for the three measures rabid and dead dogs and outbreak duration. (TIFF) [file pntd.0003876.s008.tiff]

**MB Elcho**

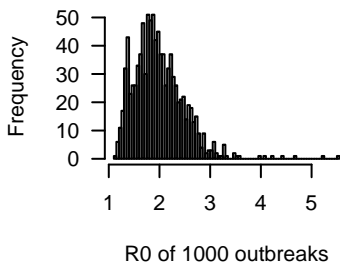

**MB NPA**

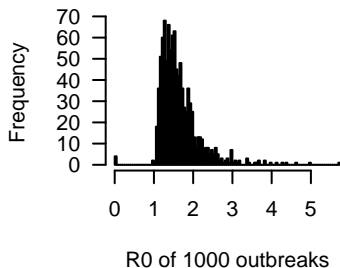

**RV Elcho**

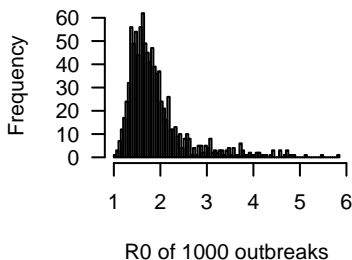

**RV NPA**

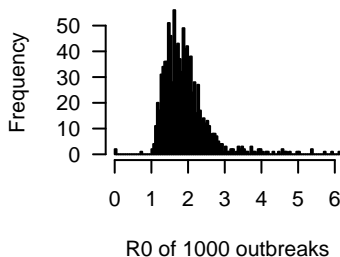

**RC Elcho**

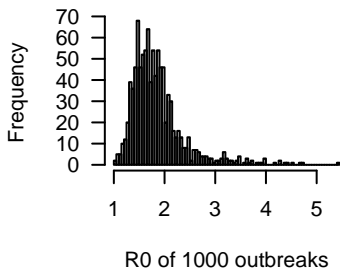

**RC NPA**

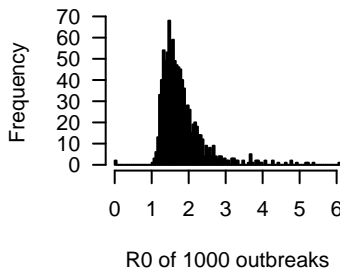

Supplement: S9 Fig — Demonstrating the independency of R0 on these control strategies which is expected for the early phase of the outbreaks. (PDF) [file pntd.0003876.s009.pdf]

RV\_NPA

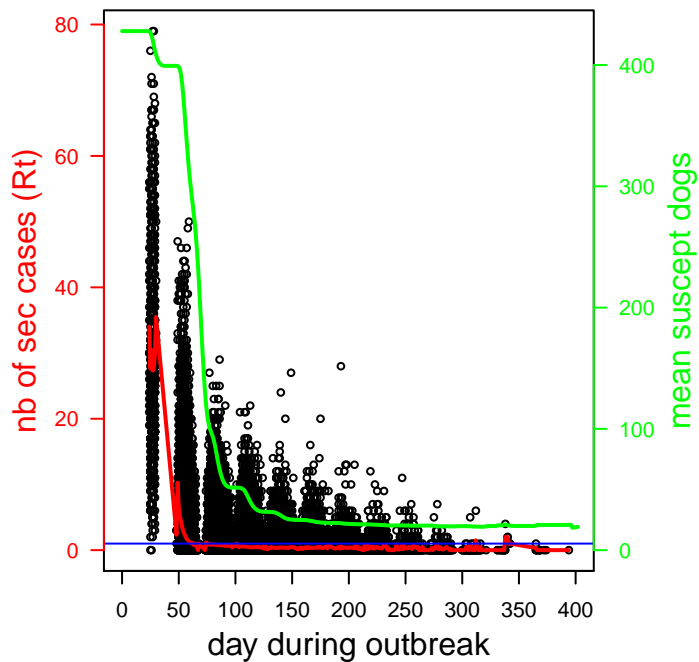

RC\_NPA

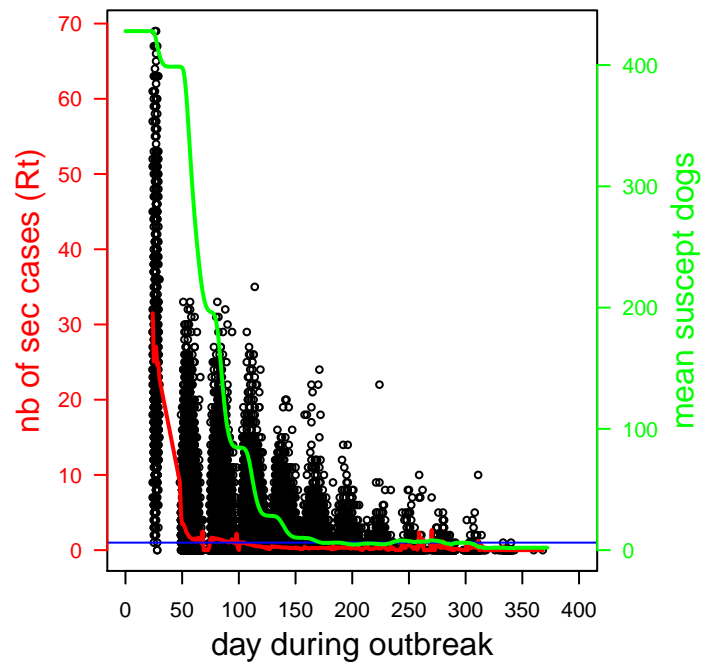

MB\_NPA

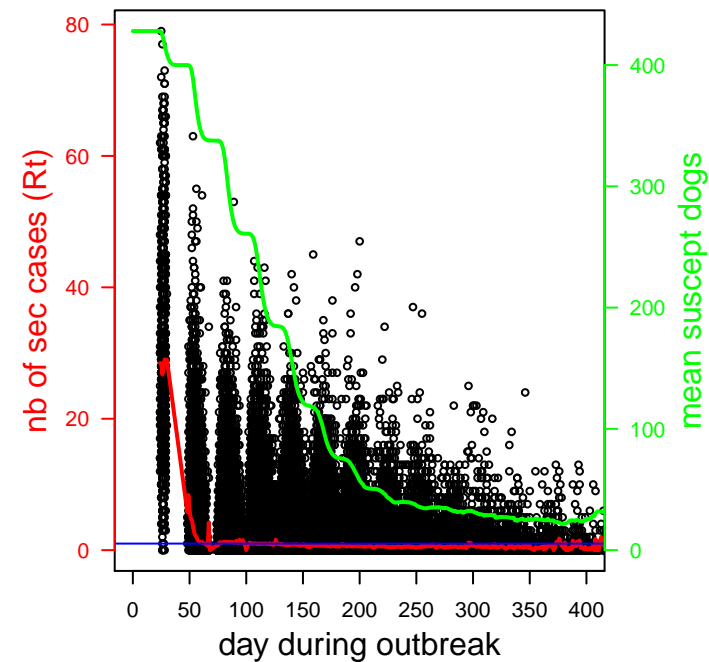

RV\_Elcho

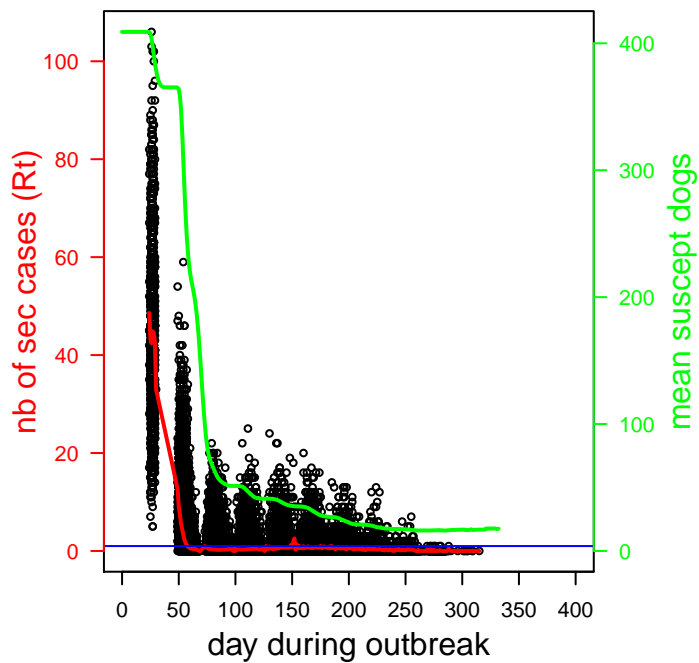

RC\_Elcho

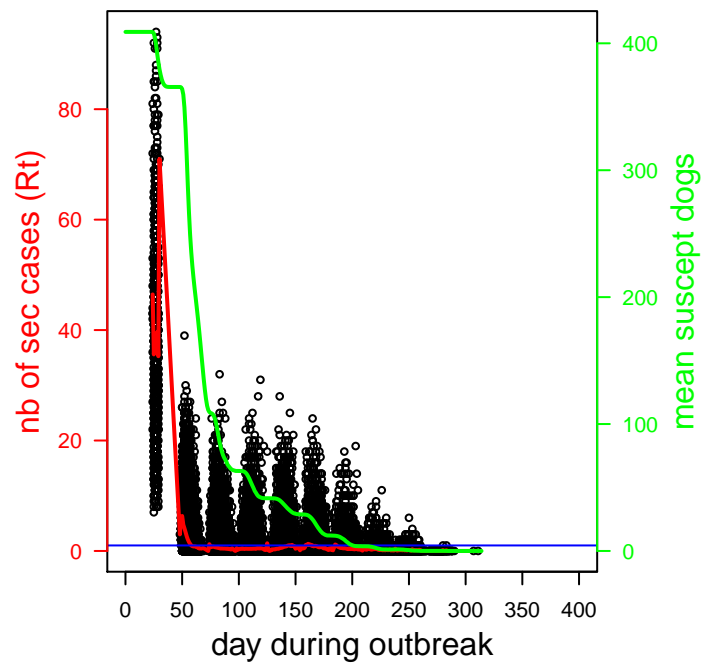

MB\_Elcho

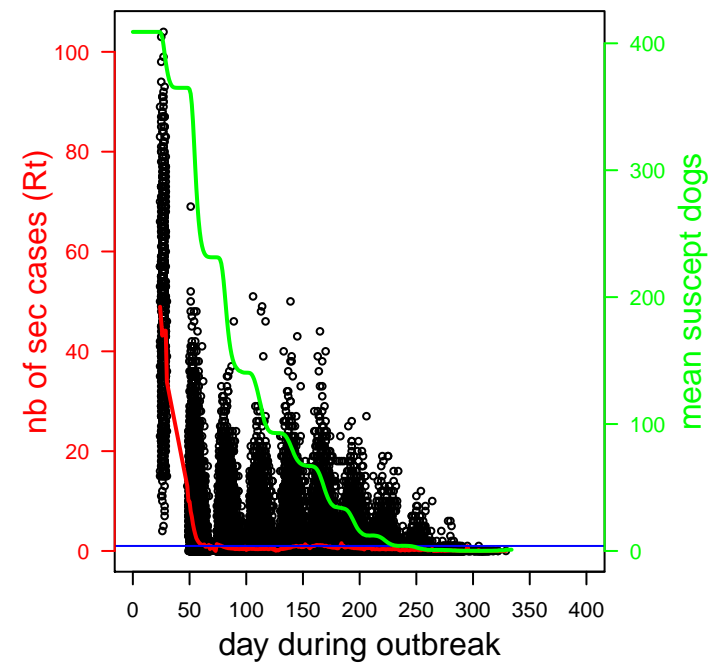

Supplement: S10 Fig — The time value (x-axis) for each dog was set at the day of the start of the infectious period. The green line represents the mean number of susceptible dogs in the population during the epidemic for 1000 repetitions per scenario. The blue horizontal line is drawn at 1, the critical value of R0. Over the duration of the outbreak, the effective reproductive ratio Rt is decreasing, which is caused by control measures applied but also by the drop of the number of dogs in the susceptible population. (PDF) [file pntd.0003876.s010.pdf]

# NPA NI

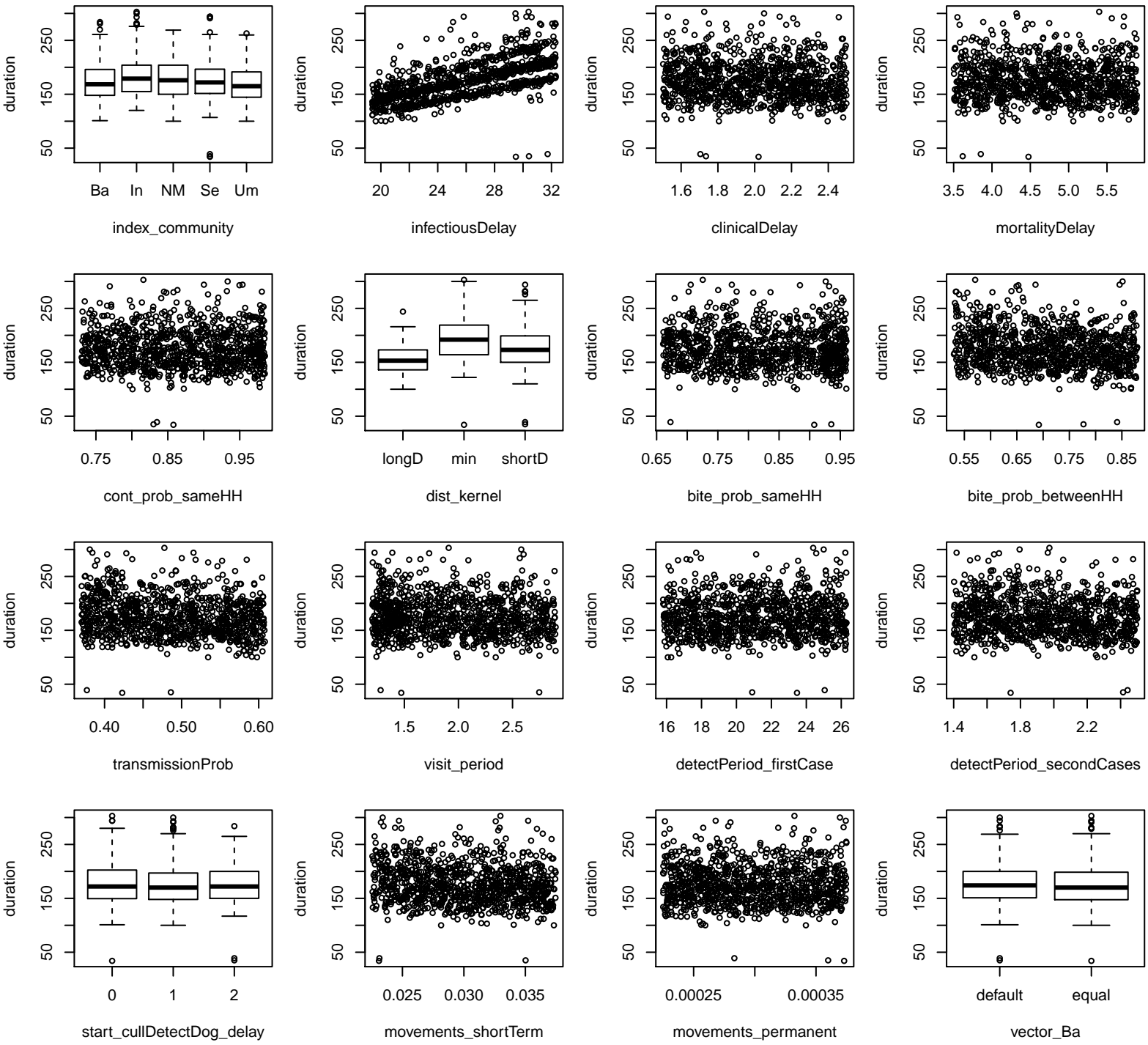

# NPA MB

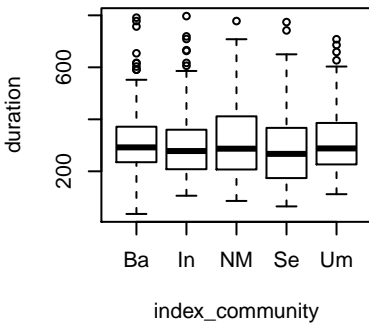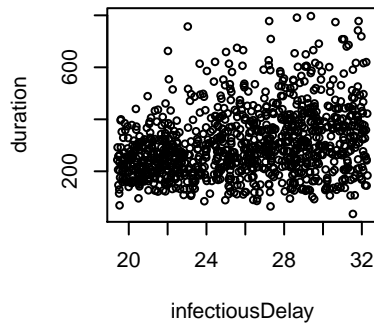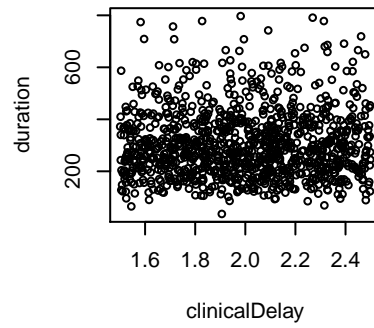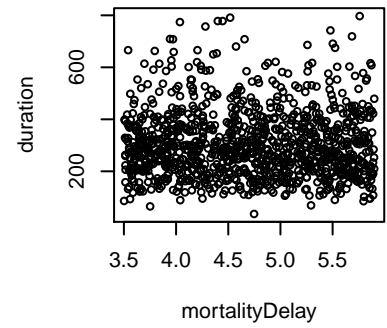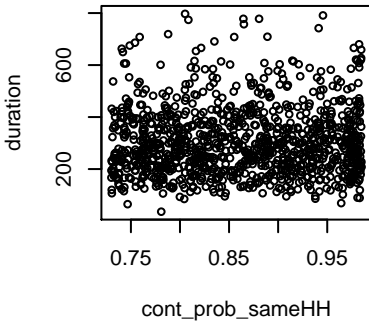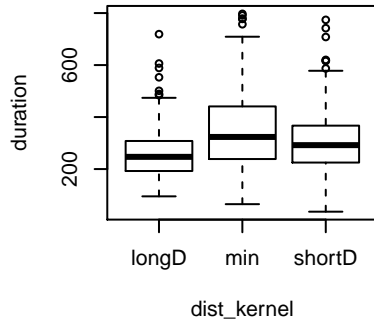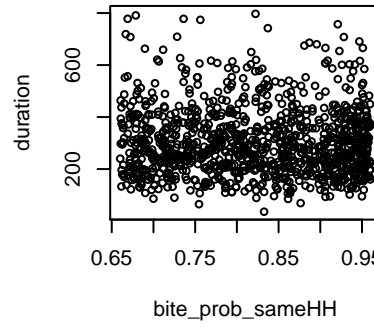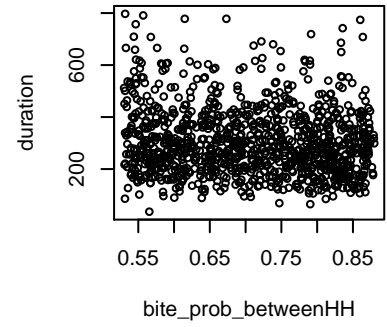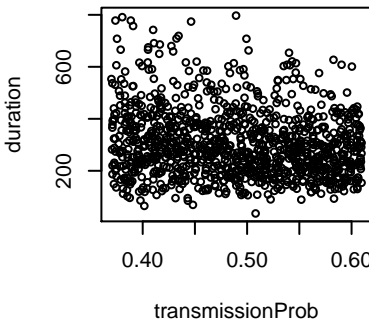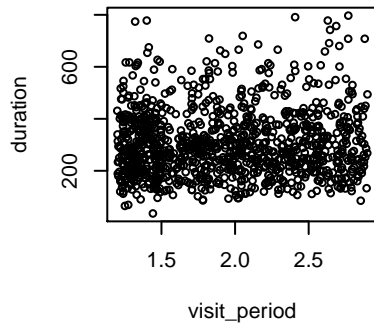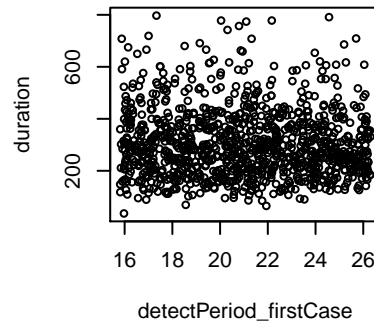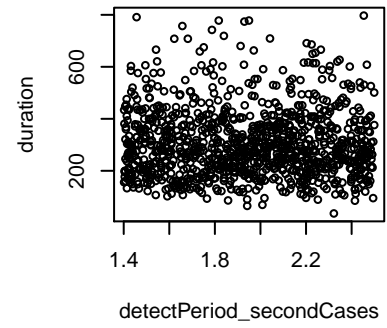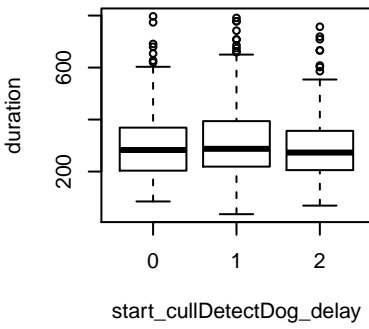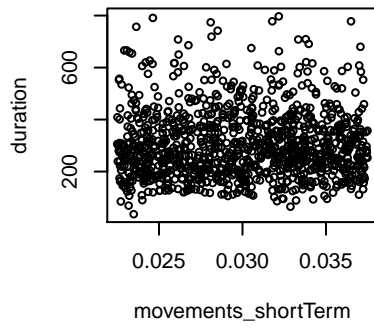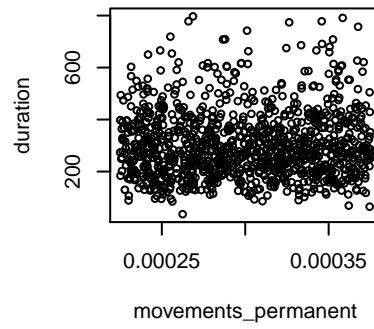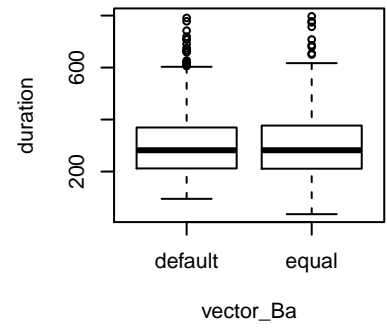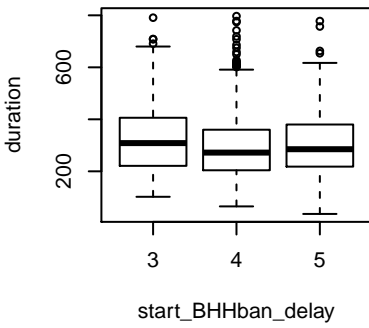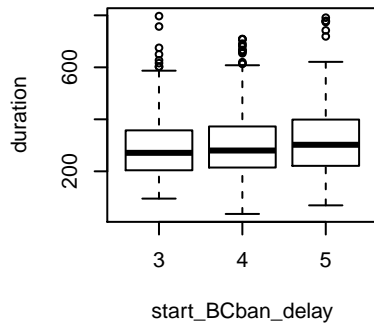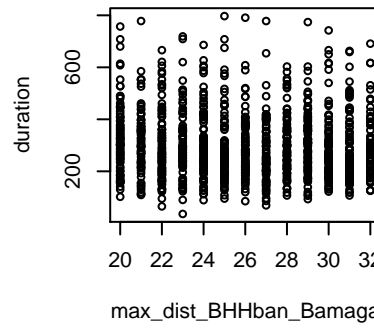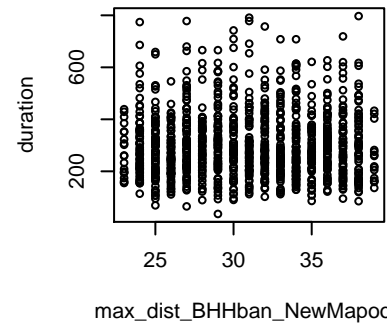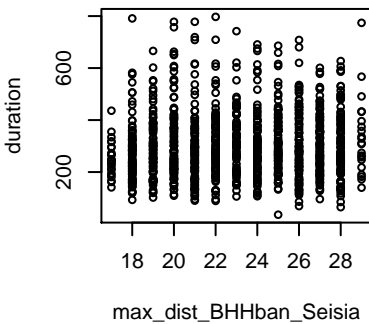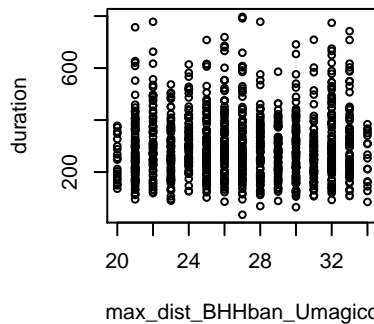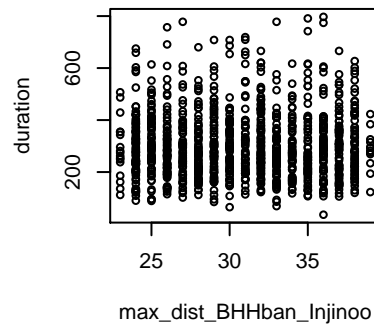

# NPA CC

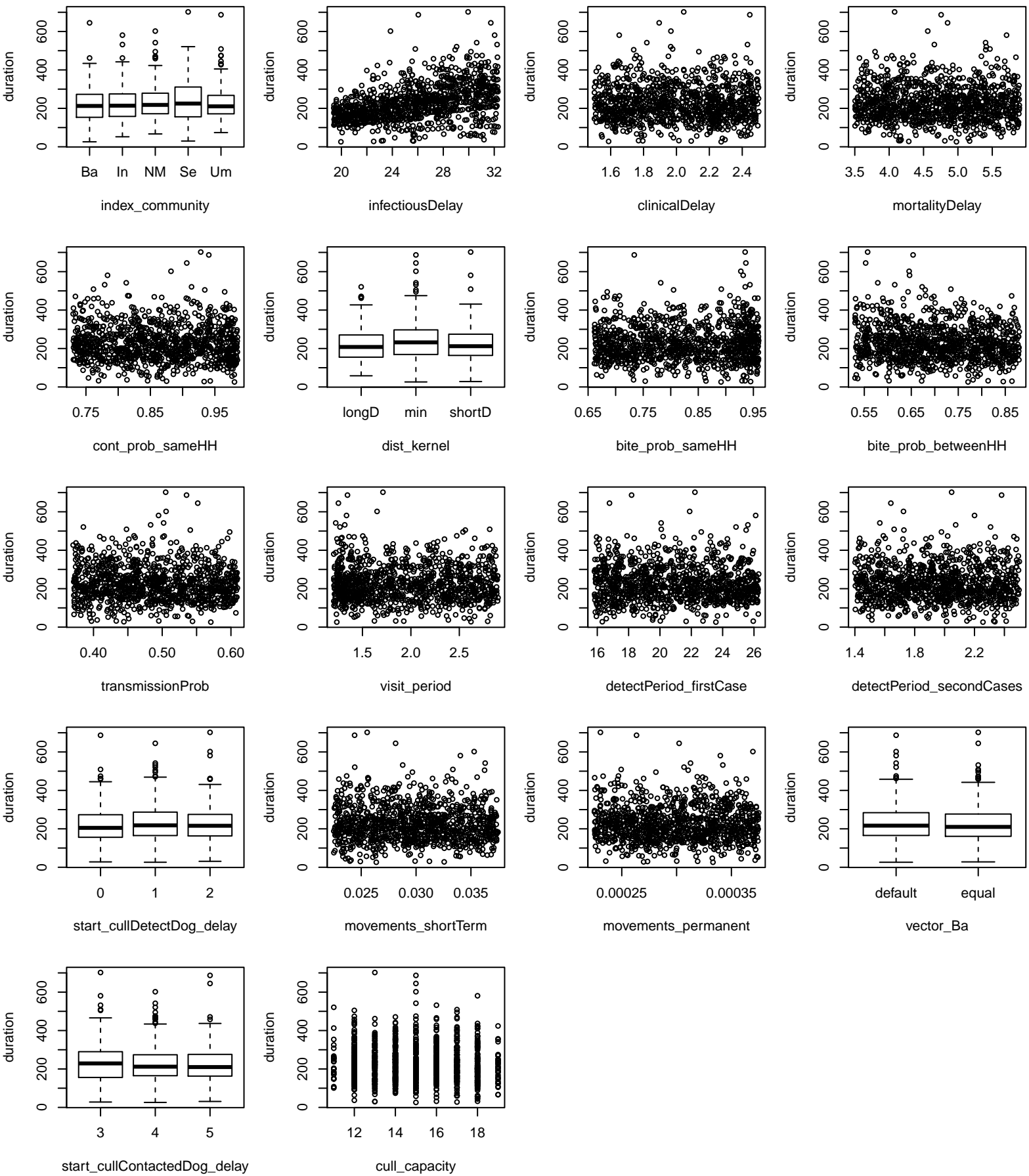

# NPA RC

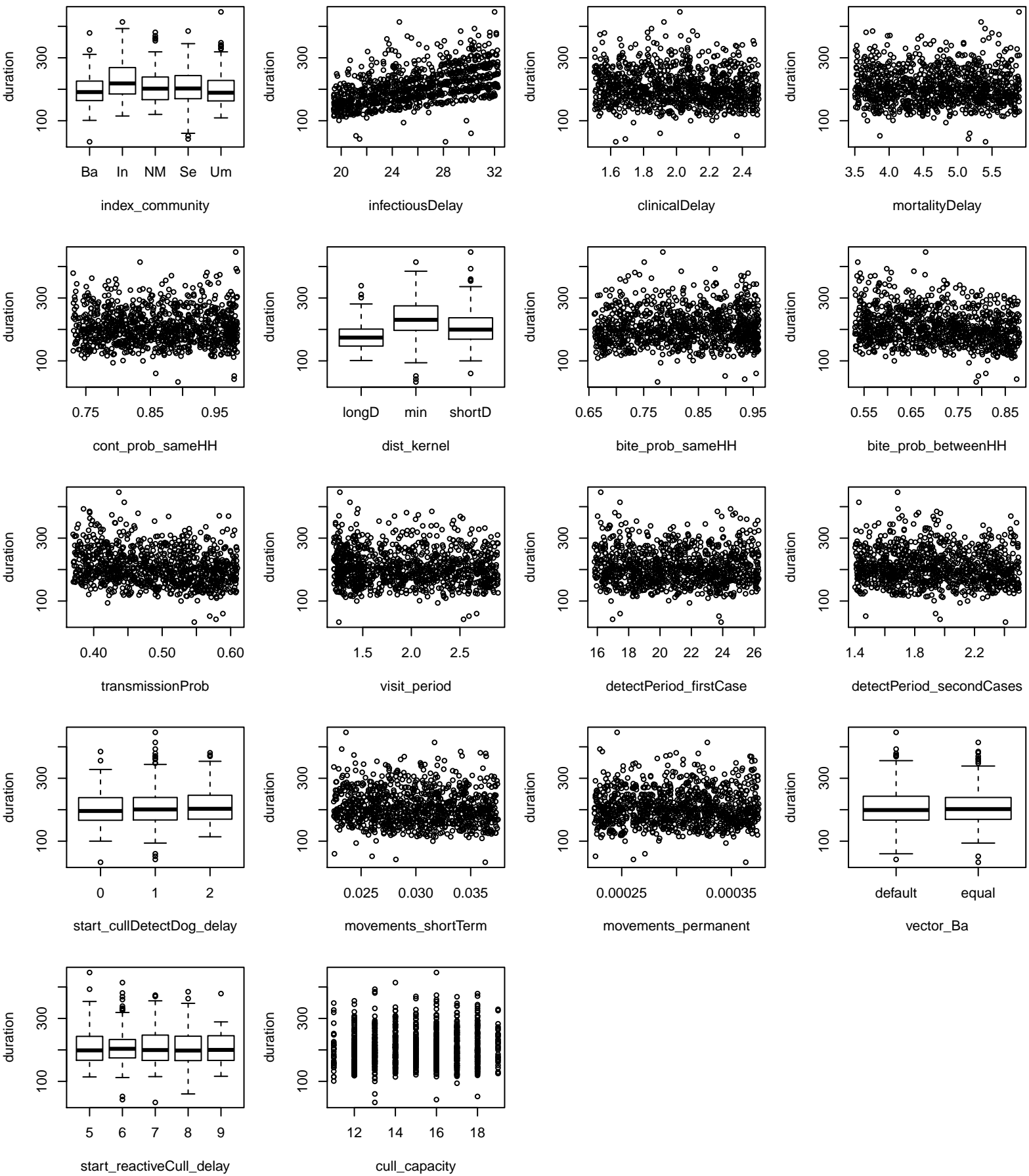

# NPA PV

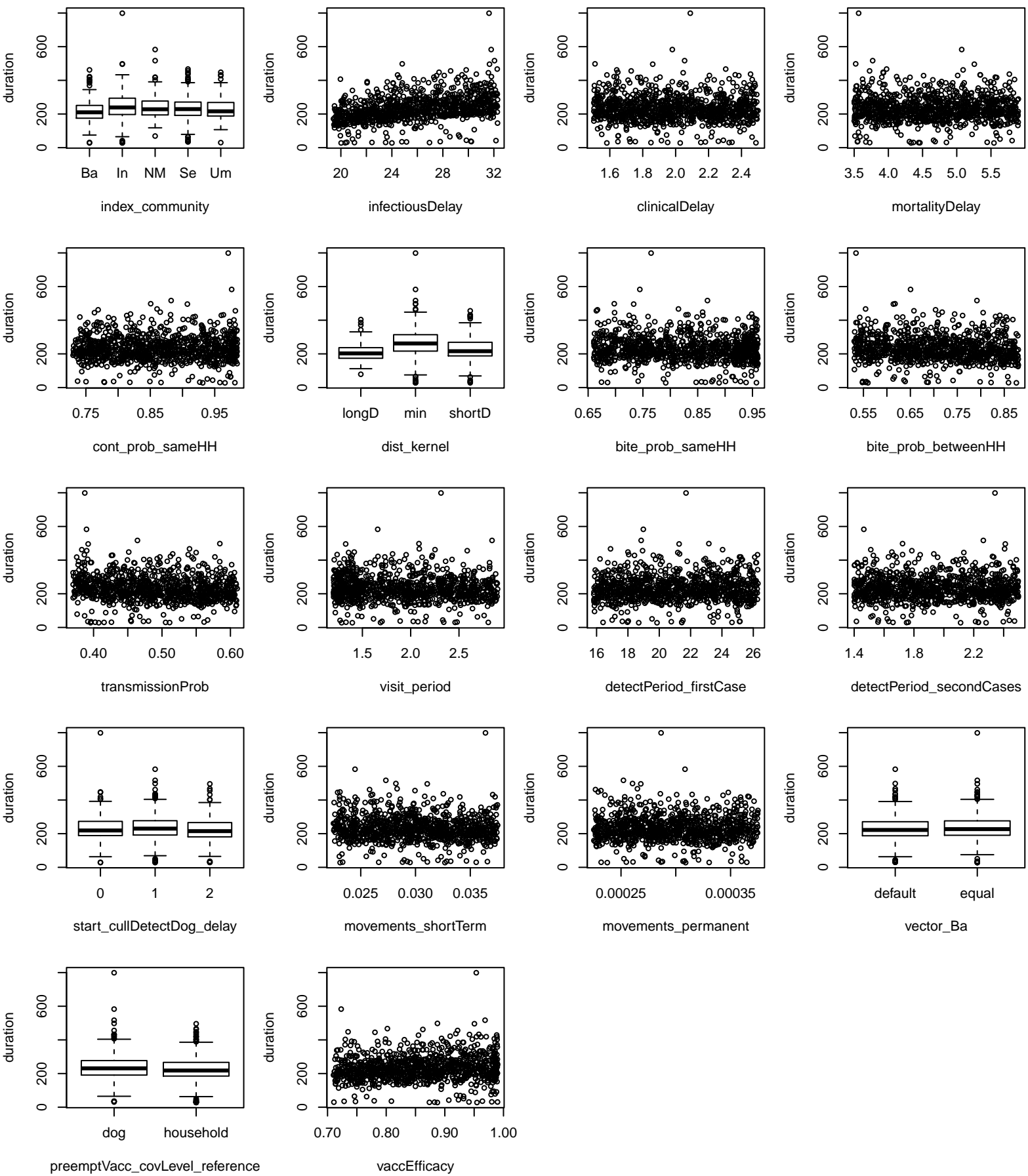

# NPA RV

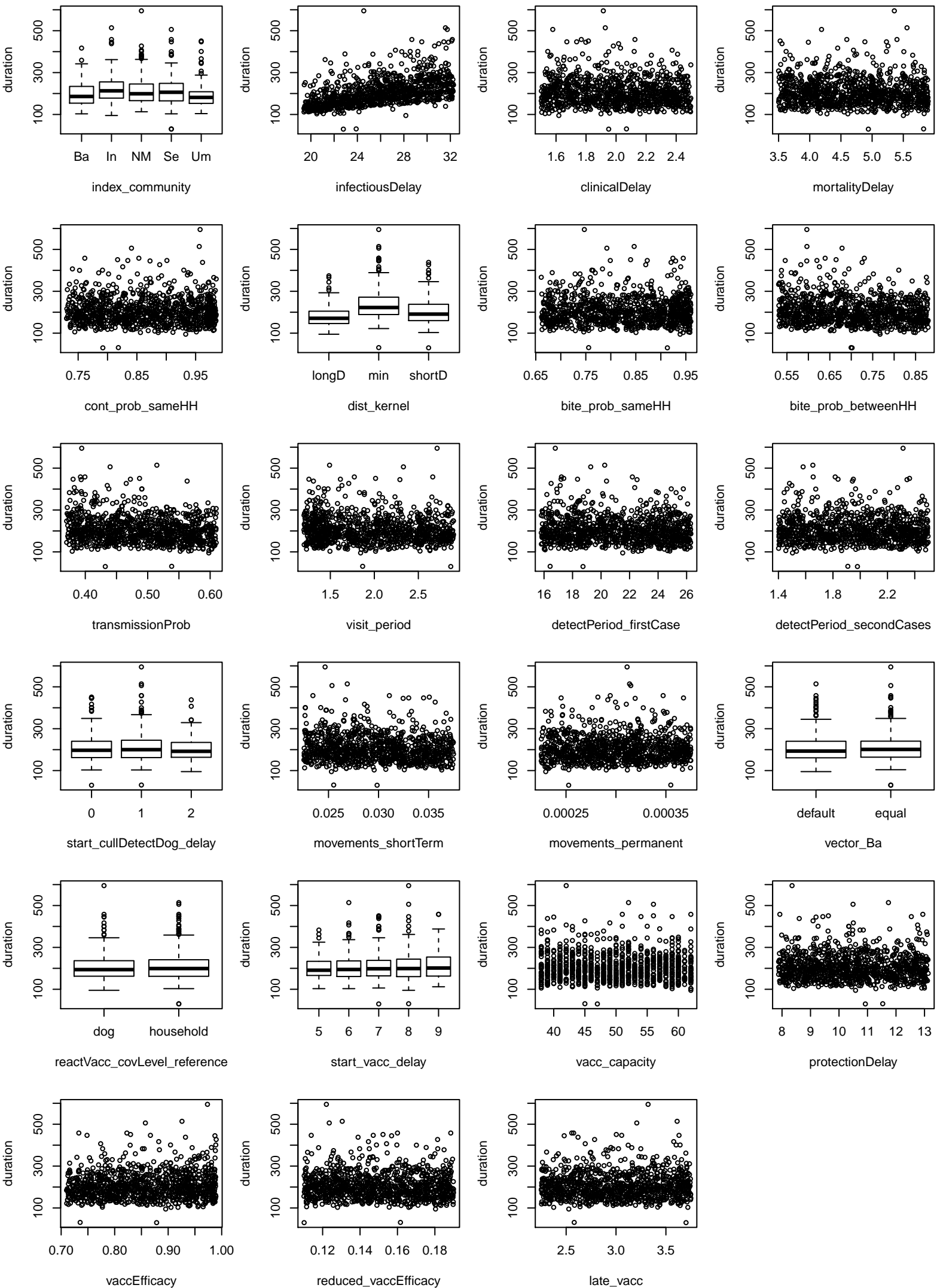

# Elcho NI

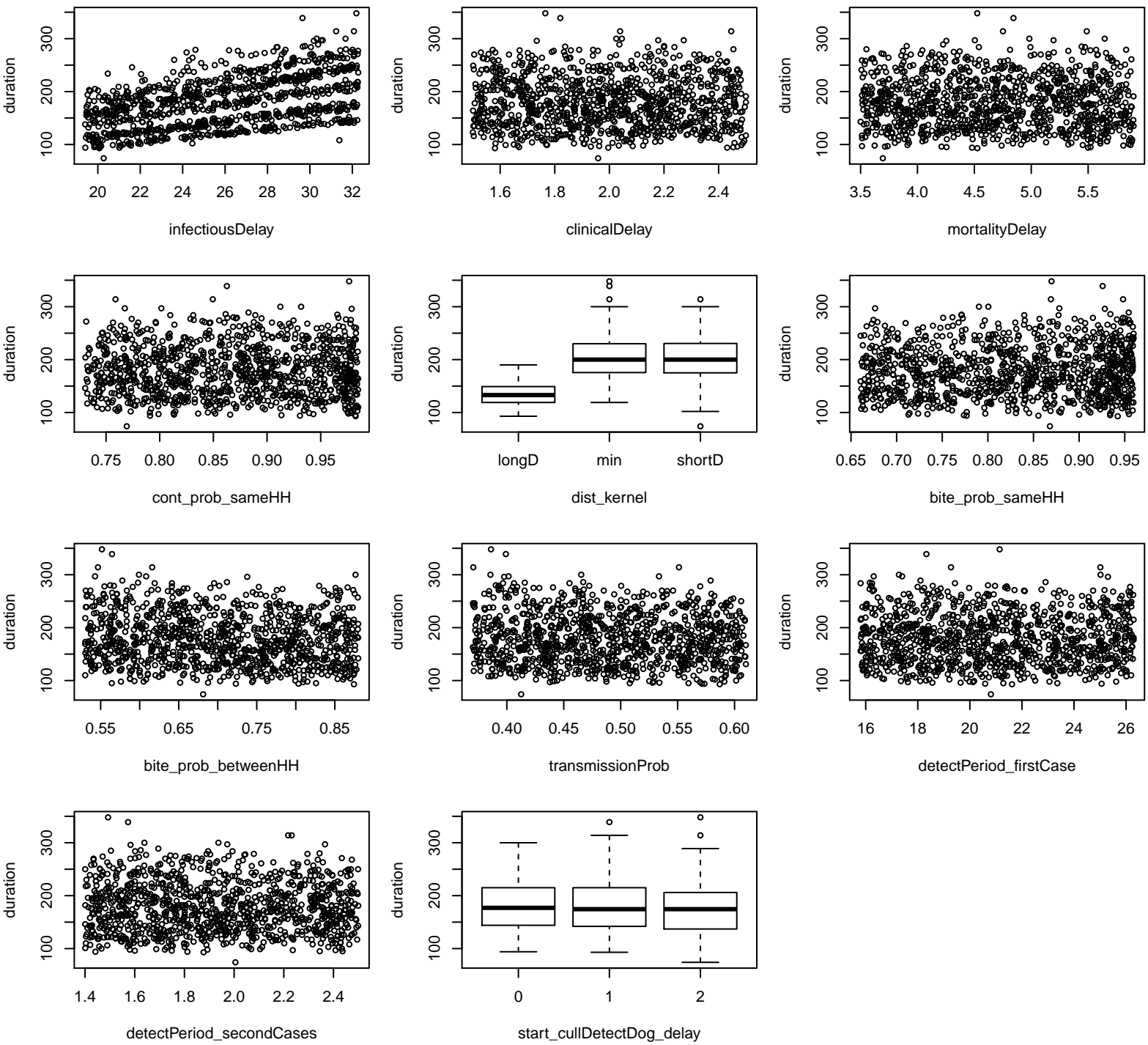

# Elcho MB

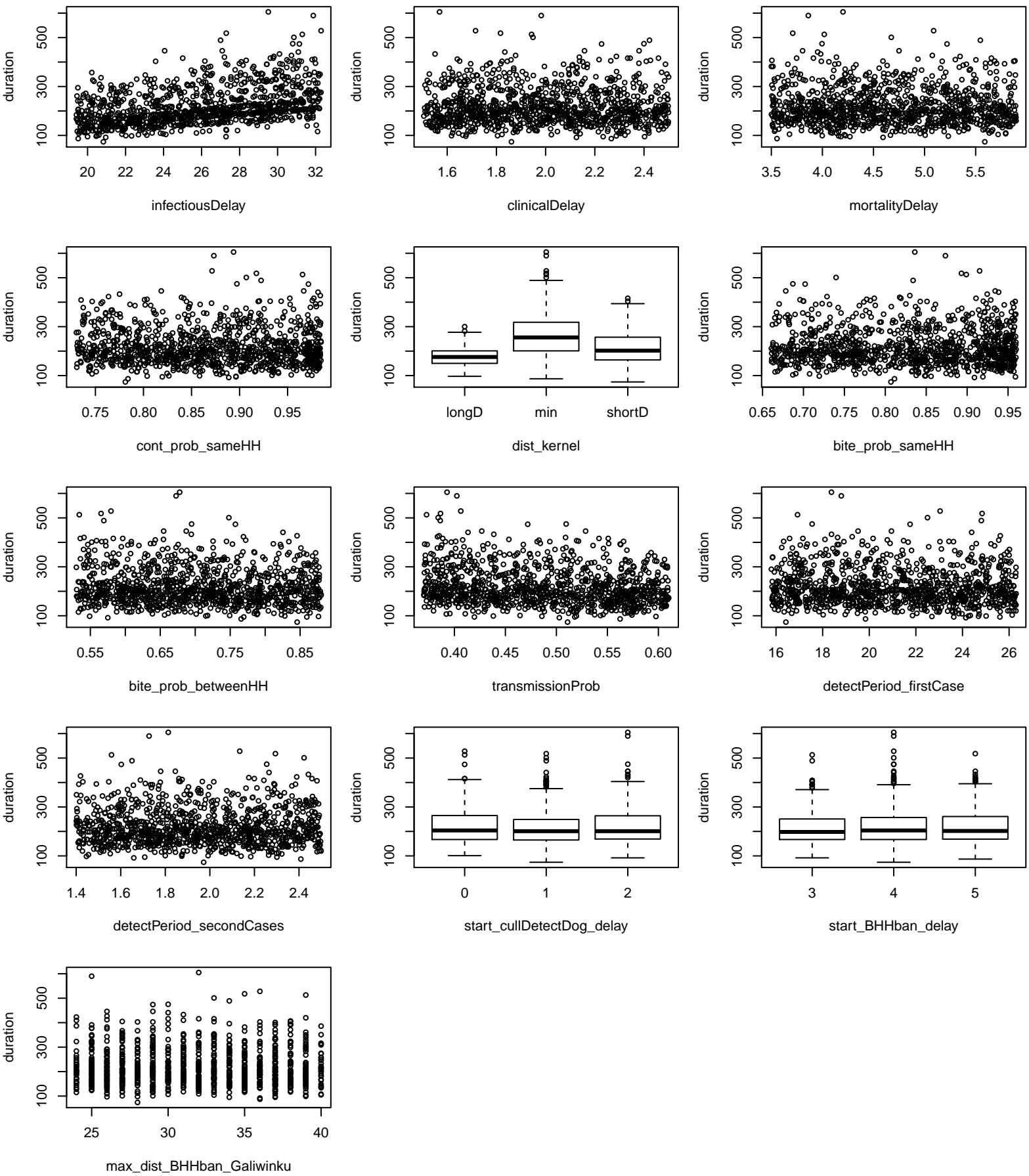

# Elcho CC

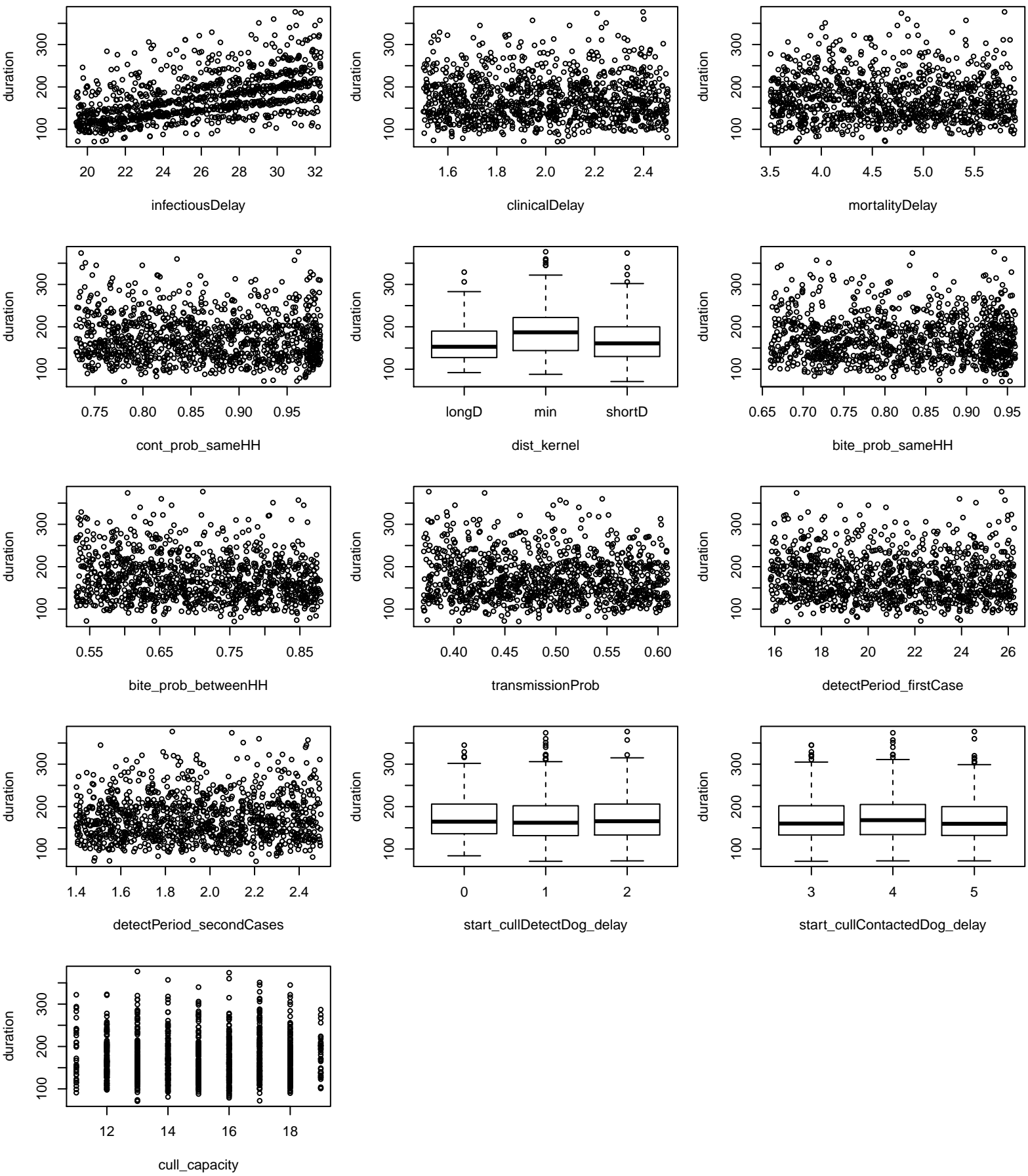

# Elcho RC

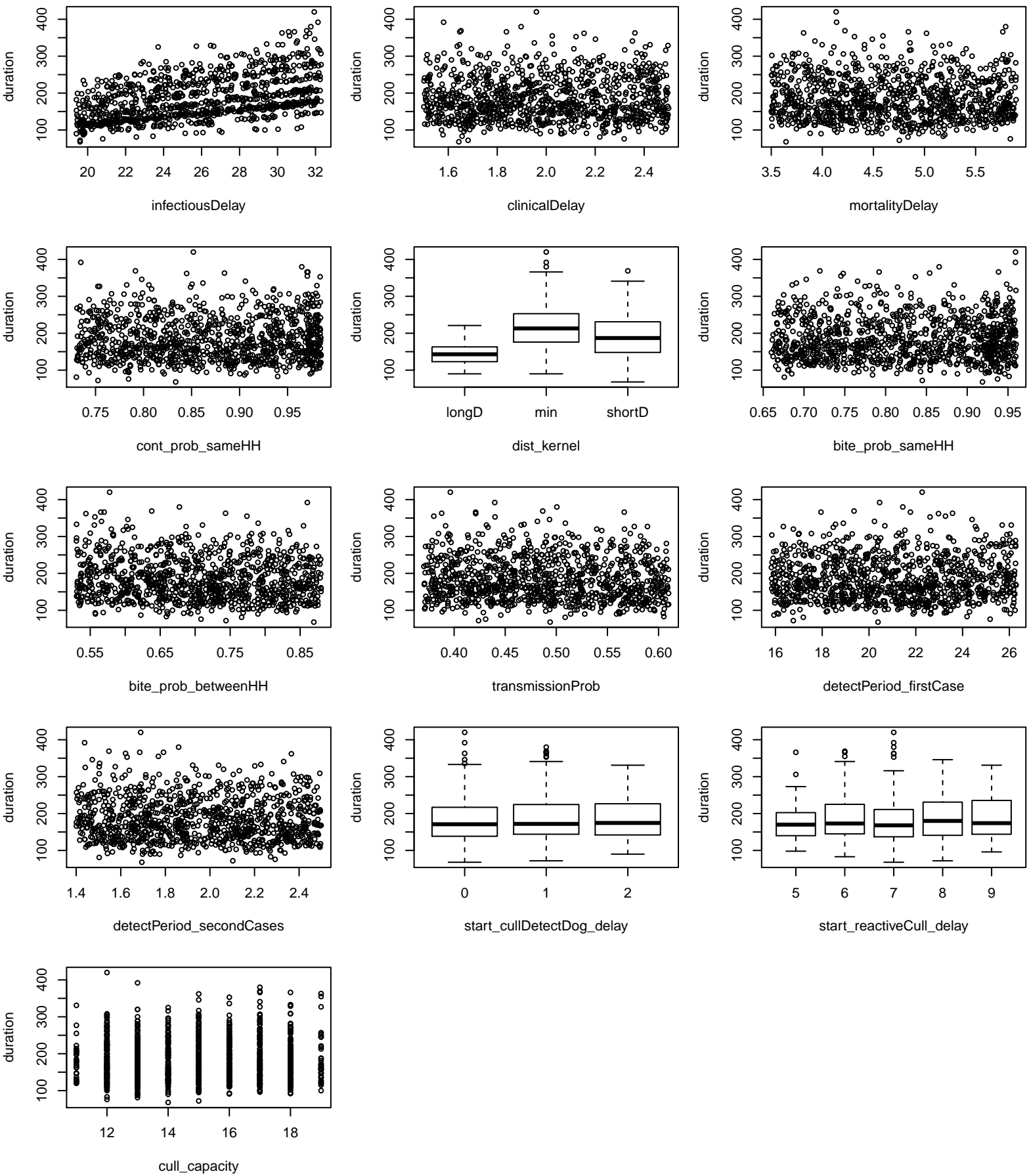

# Elcho PV

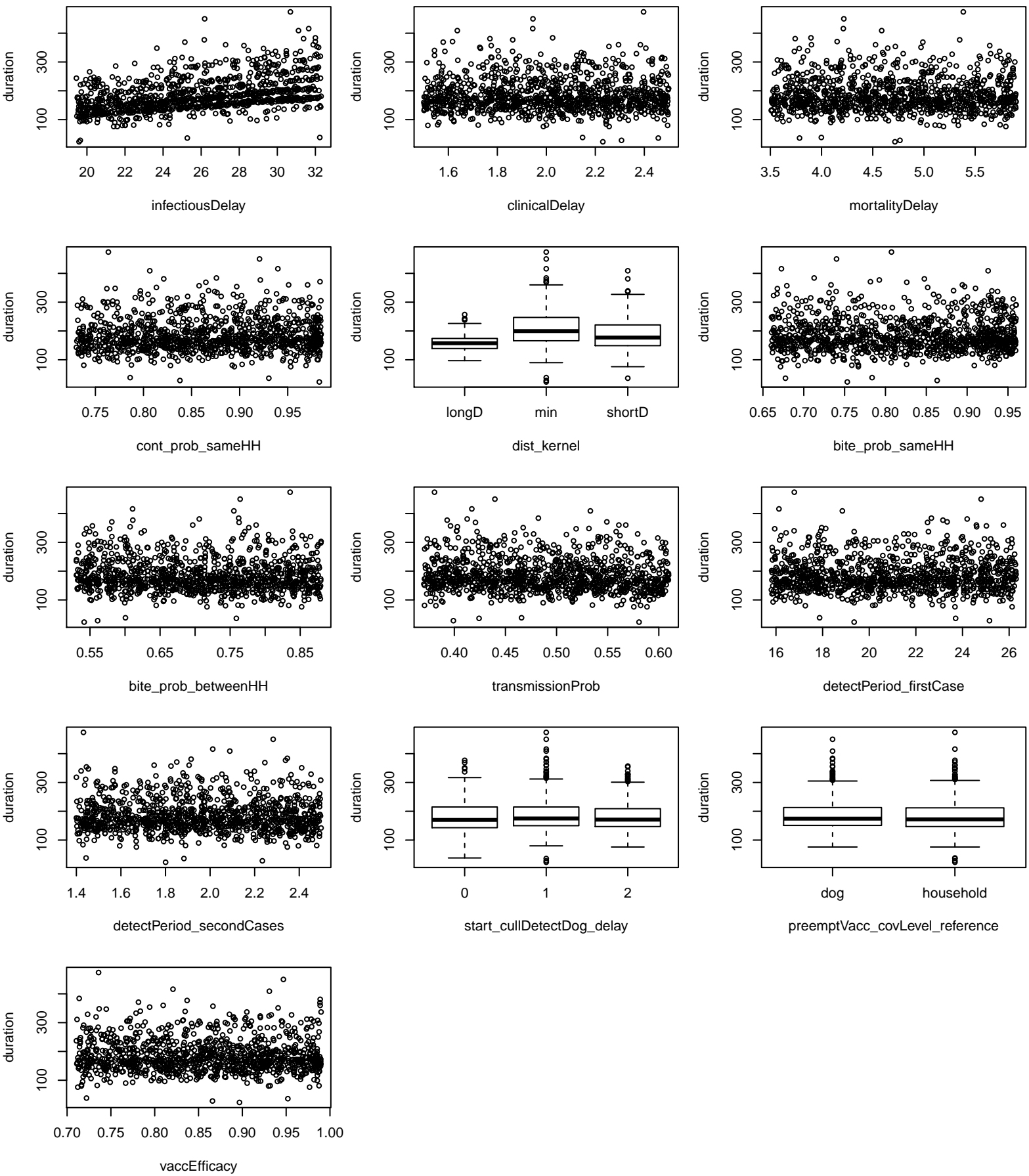

# Elcho RV

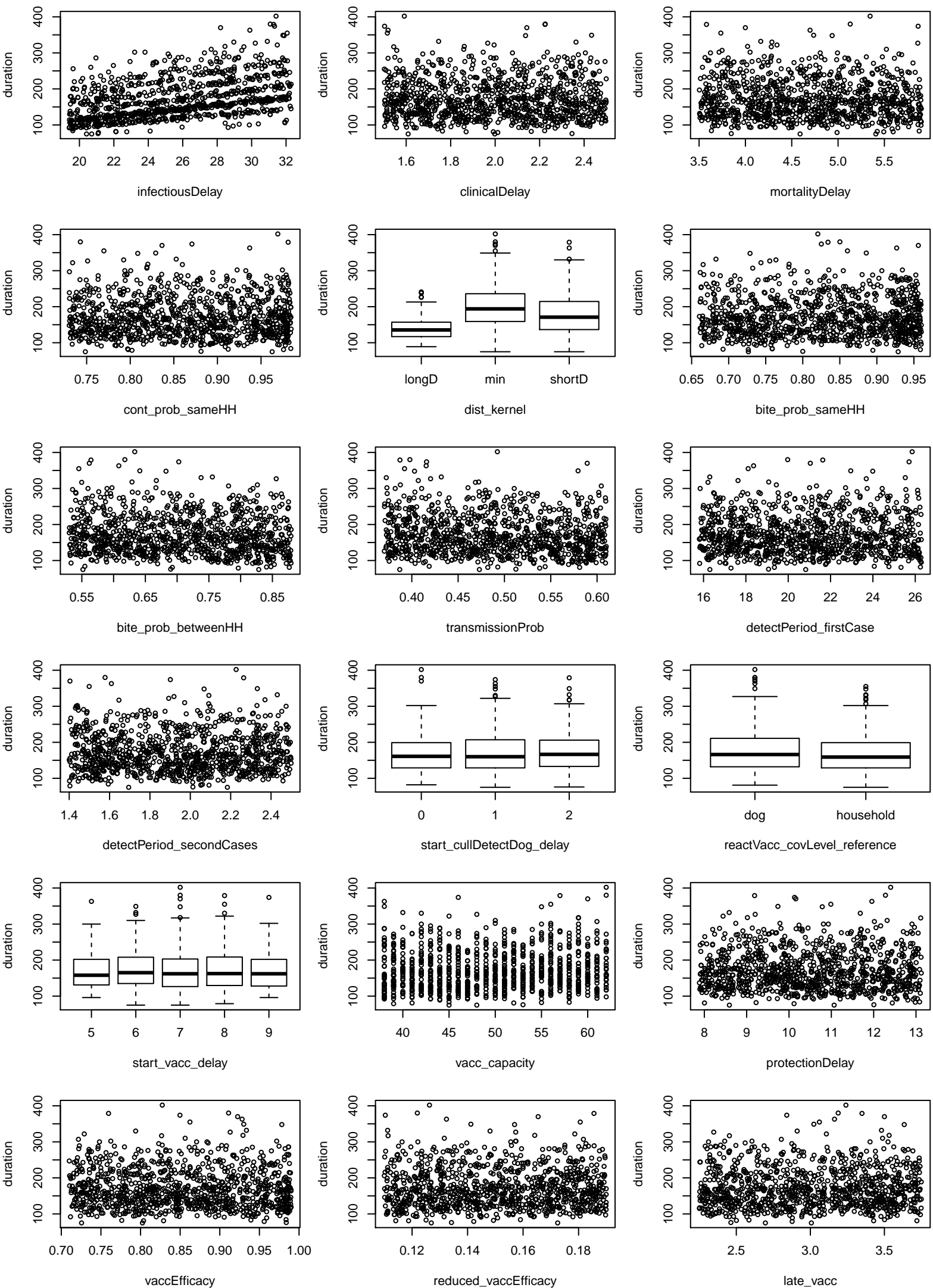

Supplement: S11 Fig — Boxplots are used to present the dependency from categorical or continuous parameters with 5 or less distinct values. The boxes in the boxplots represent the interquartile range (IQR), the horizontal line in the box the median and the whiskers extend to the most extreme data point which is no more than 1.5 times IQR from the box. The three values of the distance kernel (dist_kernel) refer to the minimal distance kernel (min), increased short distance (shortD) and long distance probability (longD) of S2 Fig. (PDF) [file pntd.0003876.s011.pdf]

A

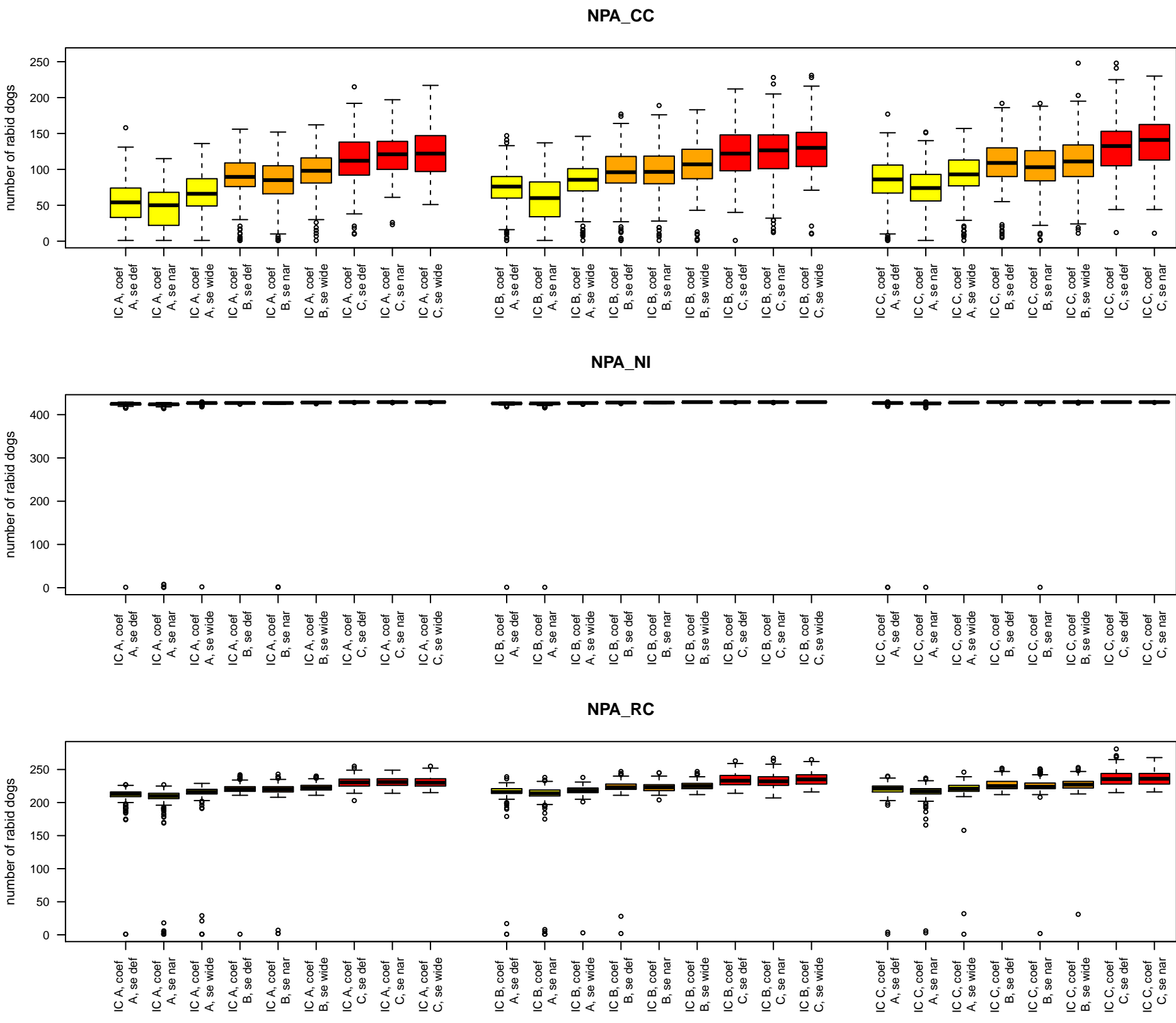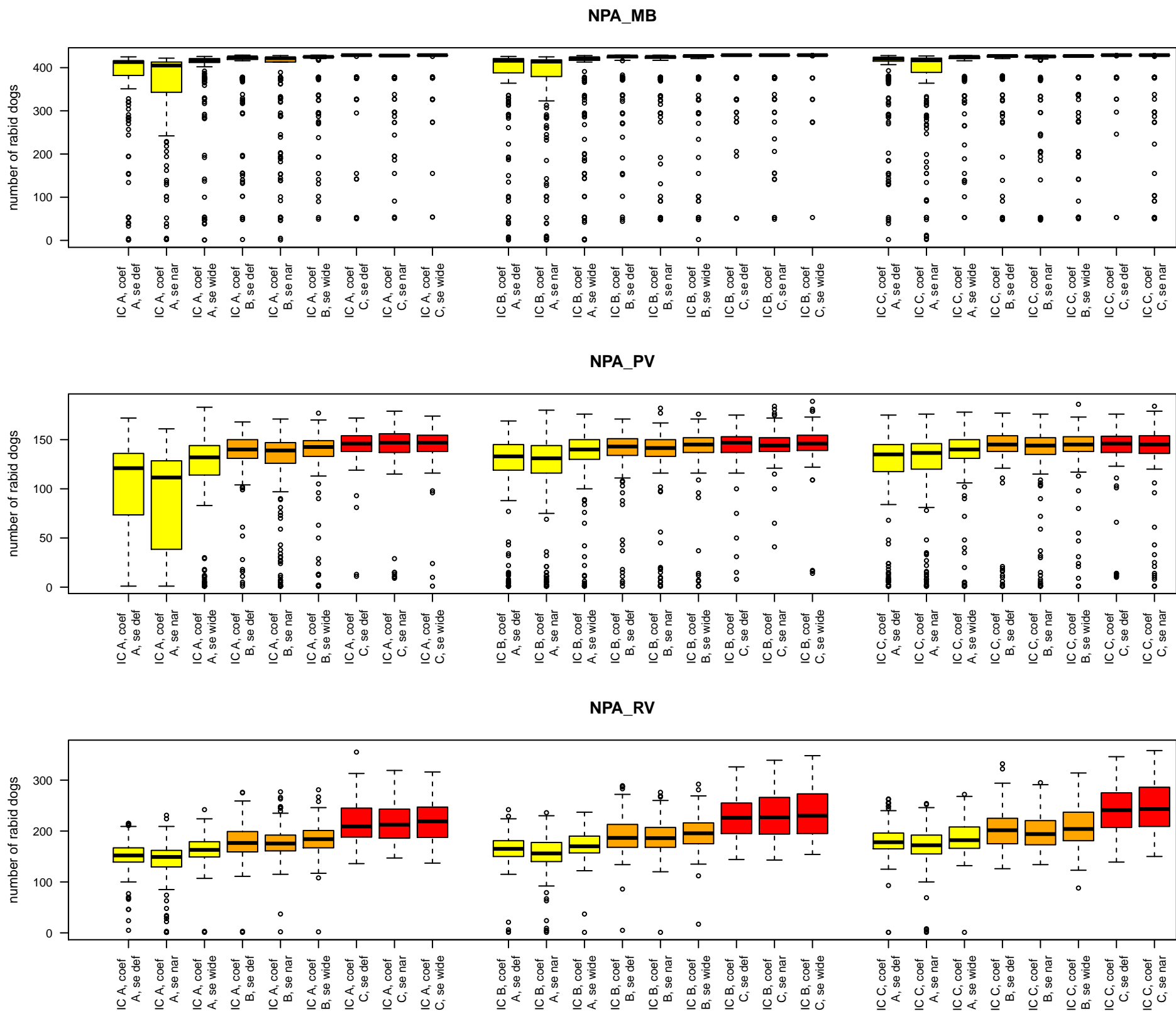

B

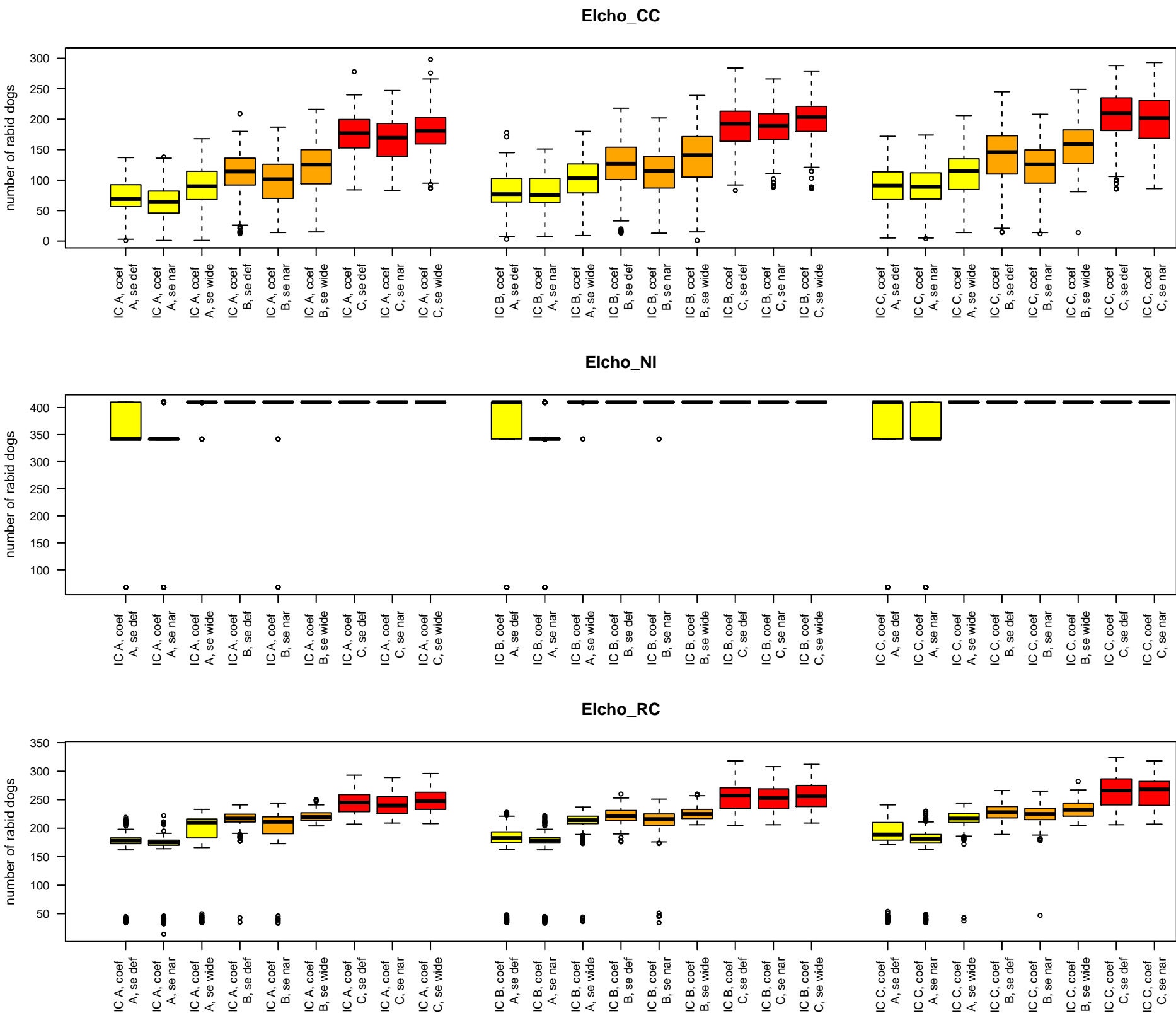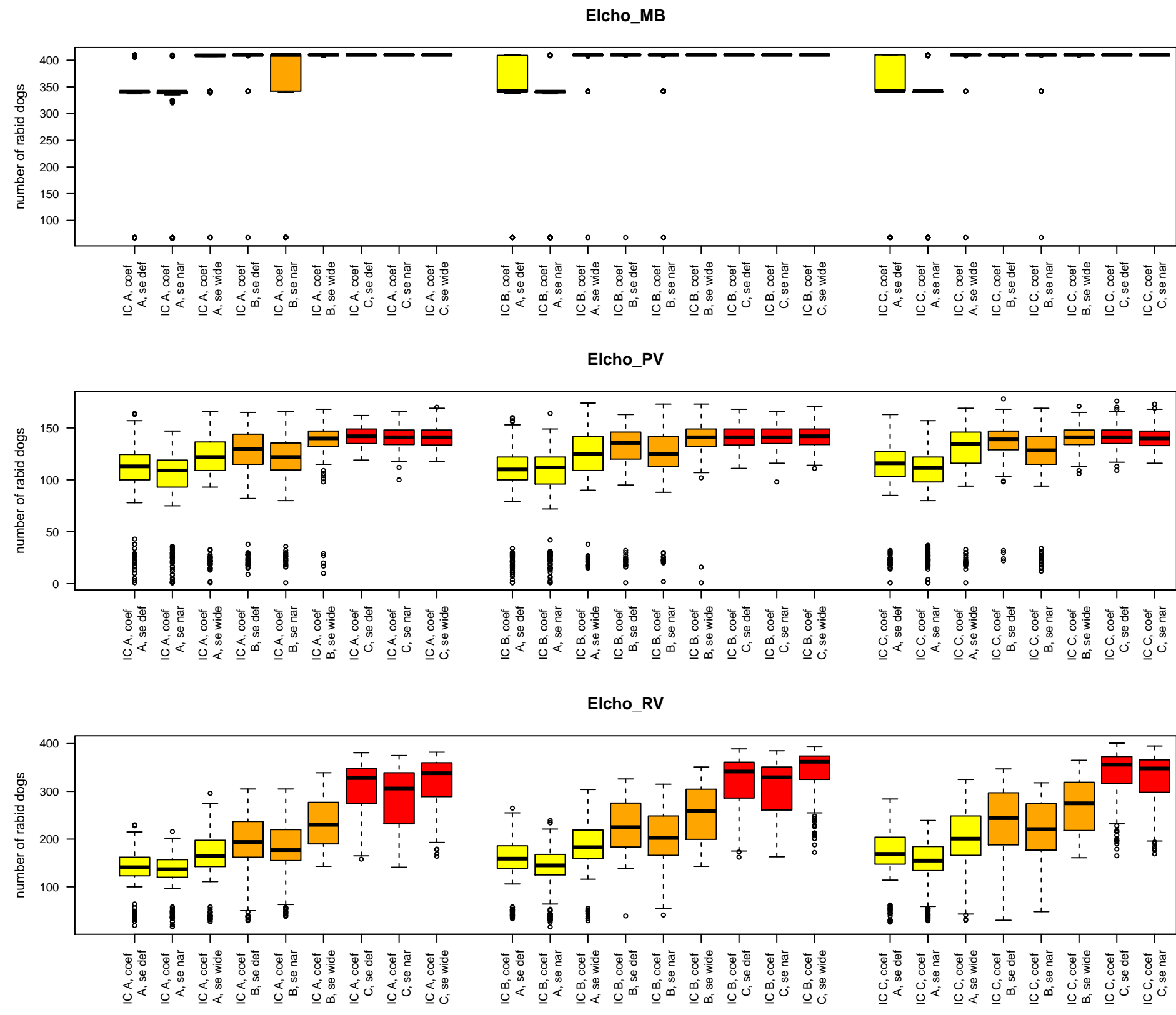

C

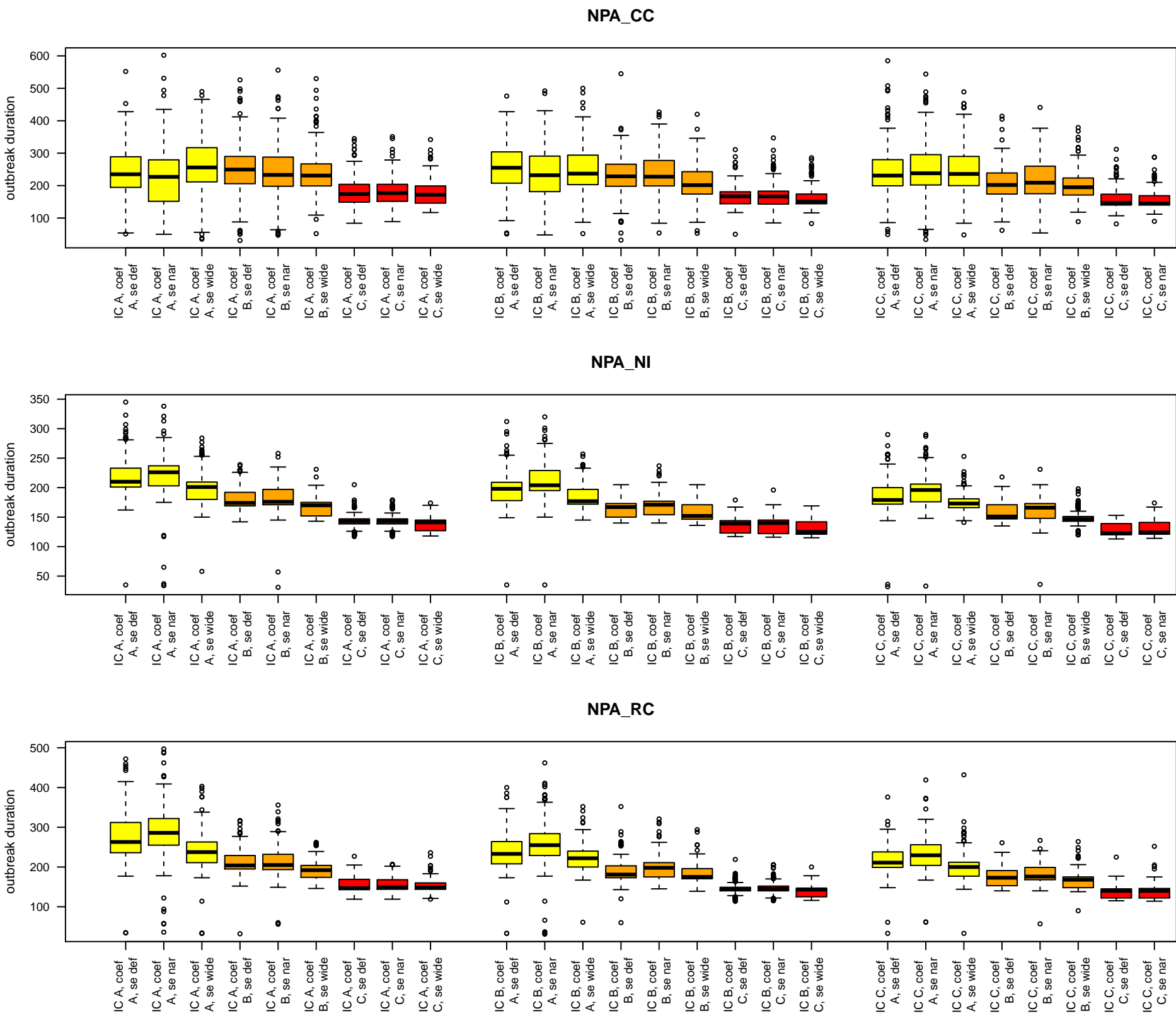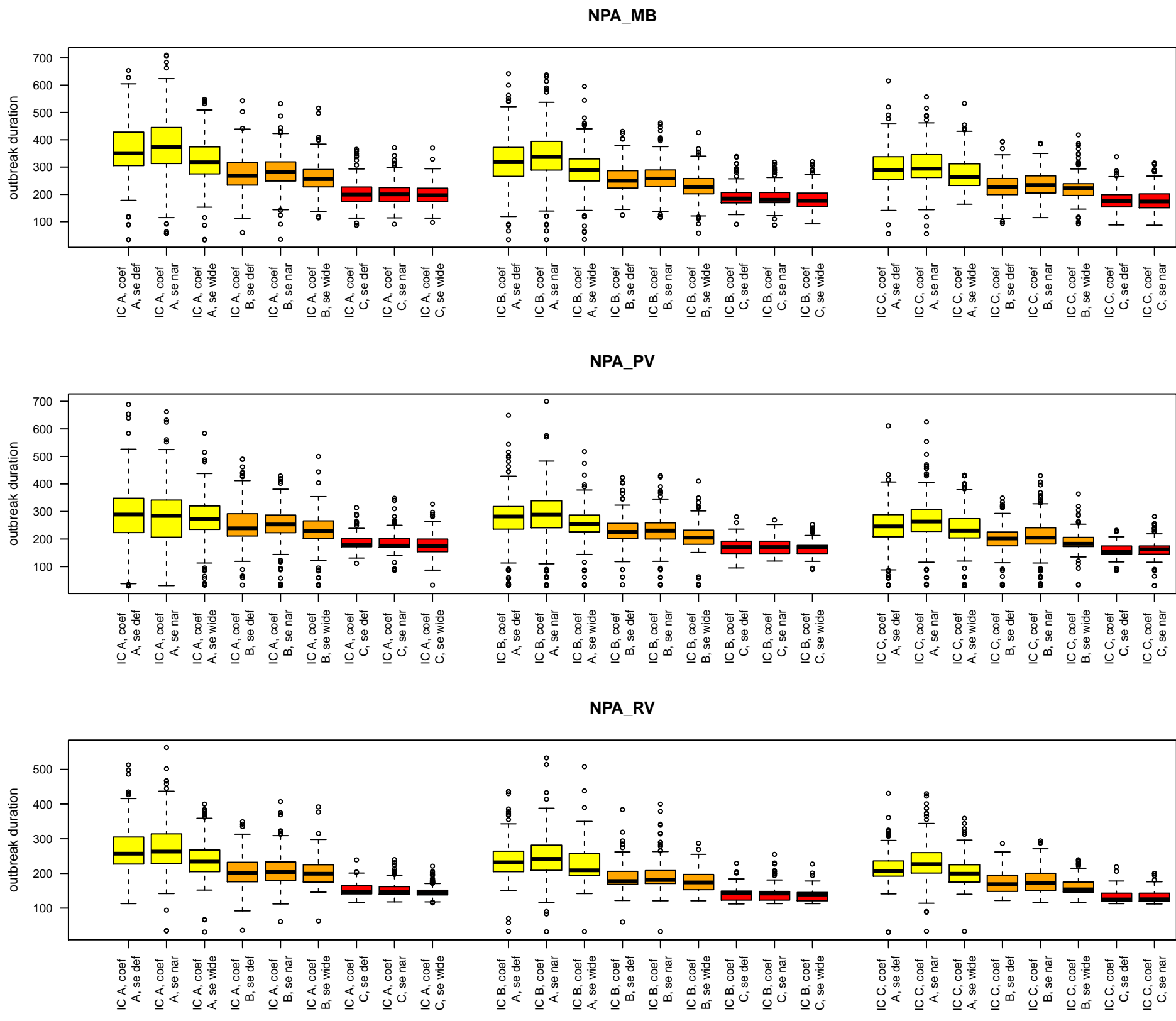

D

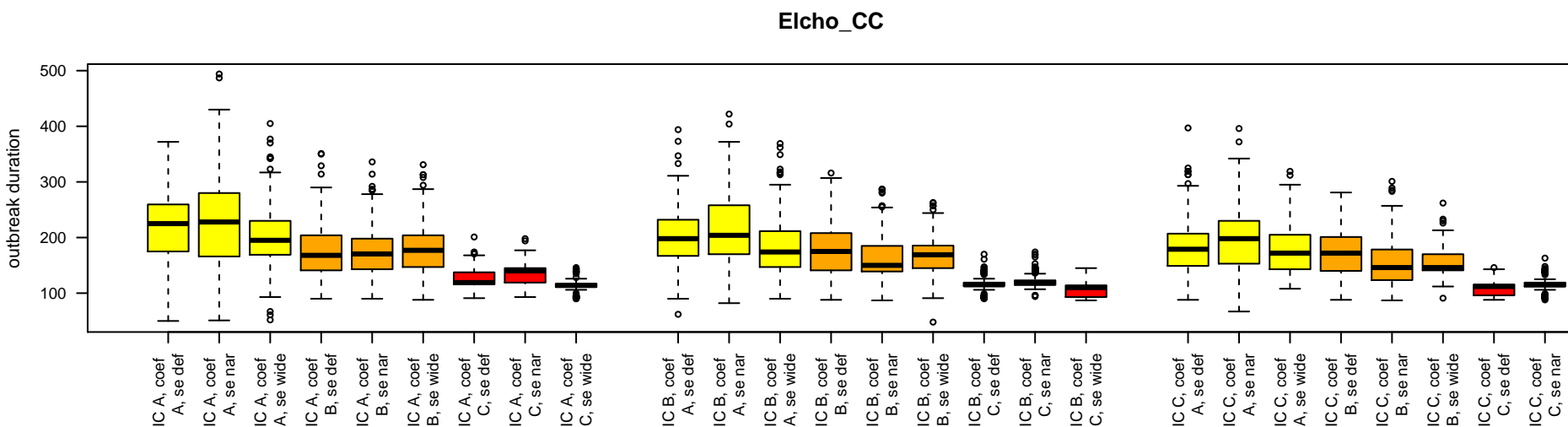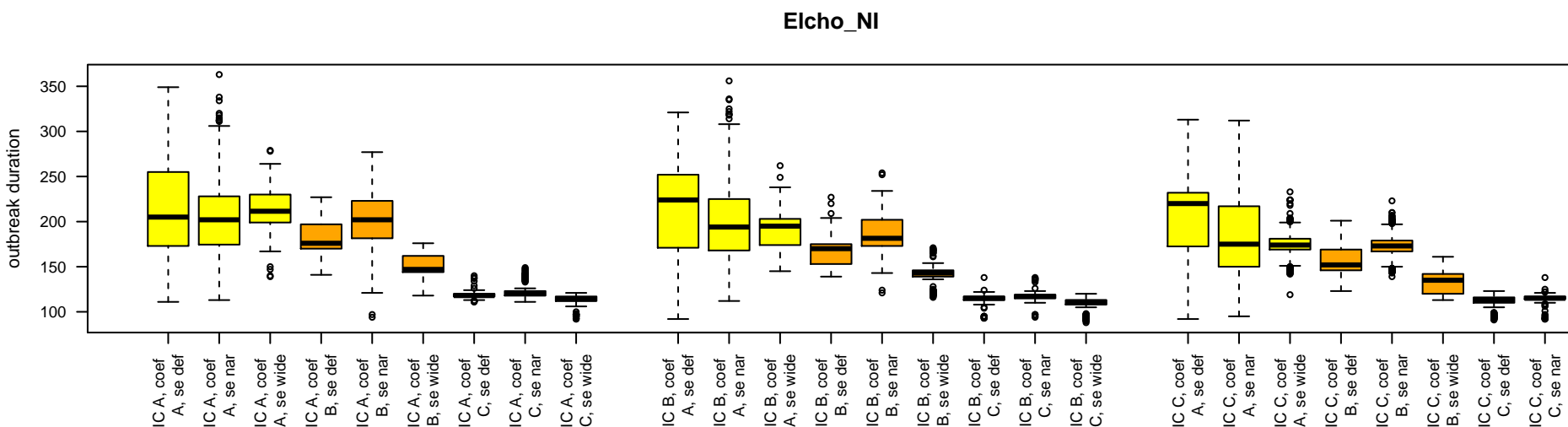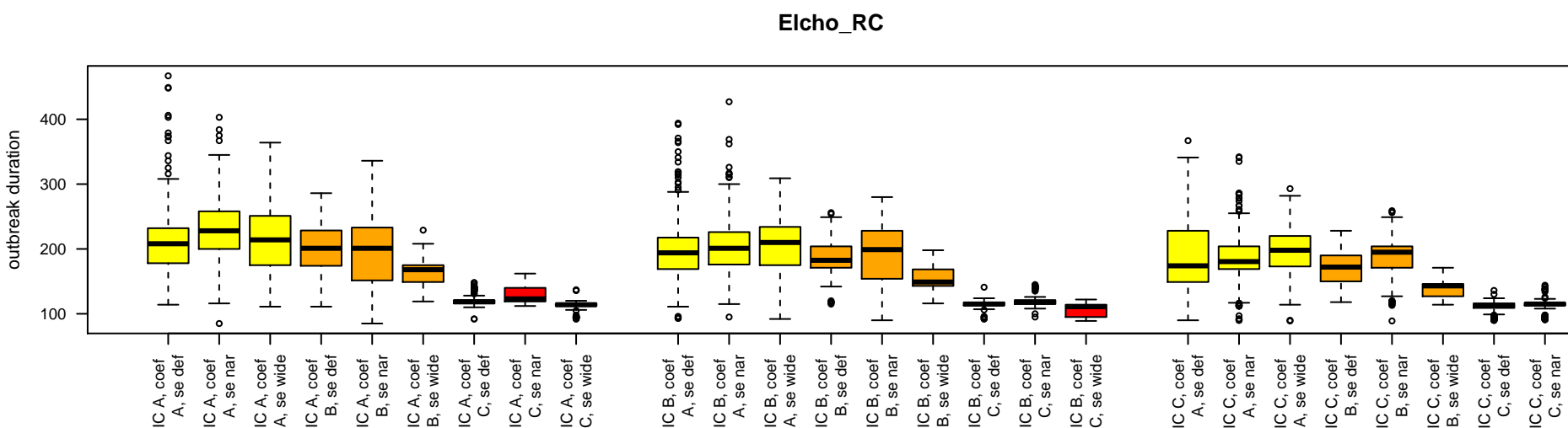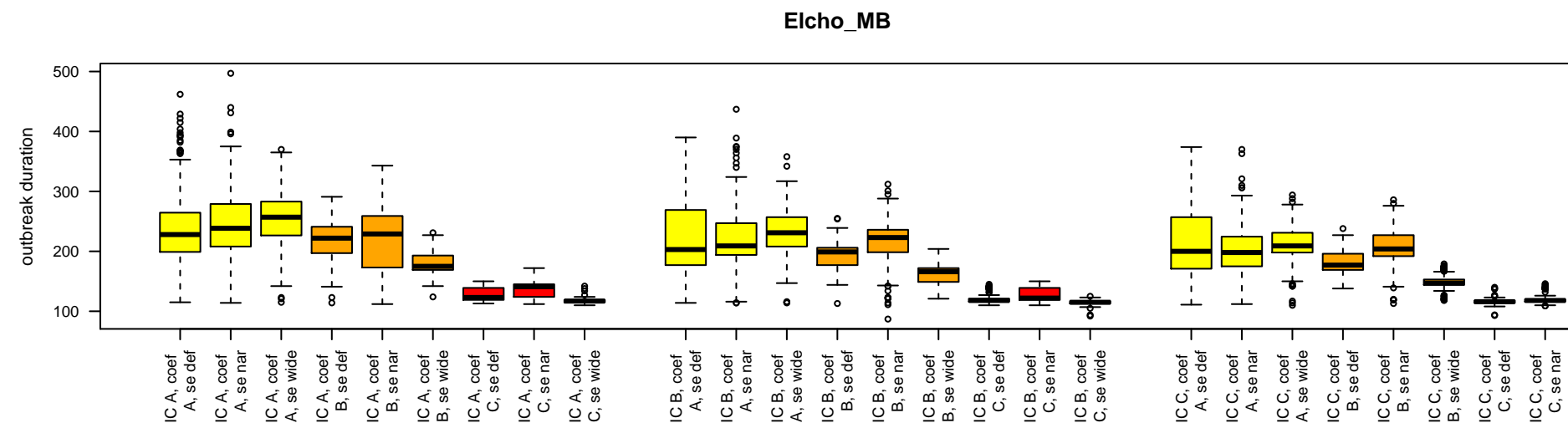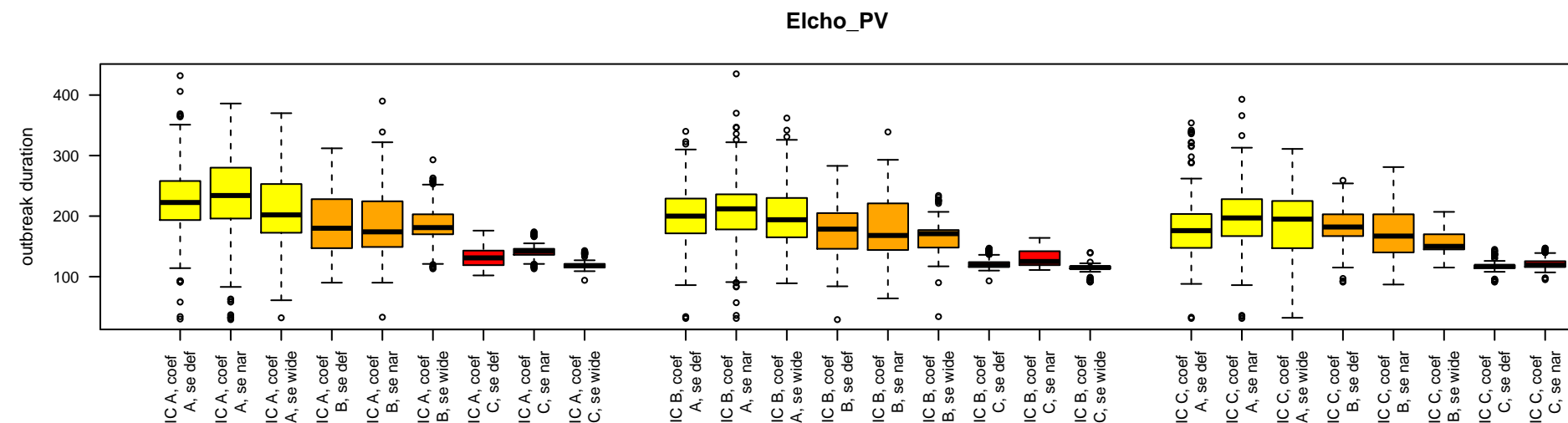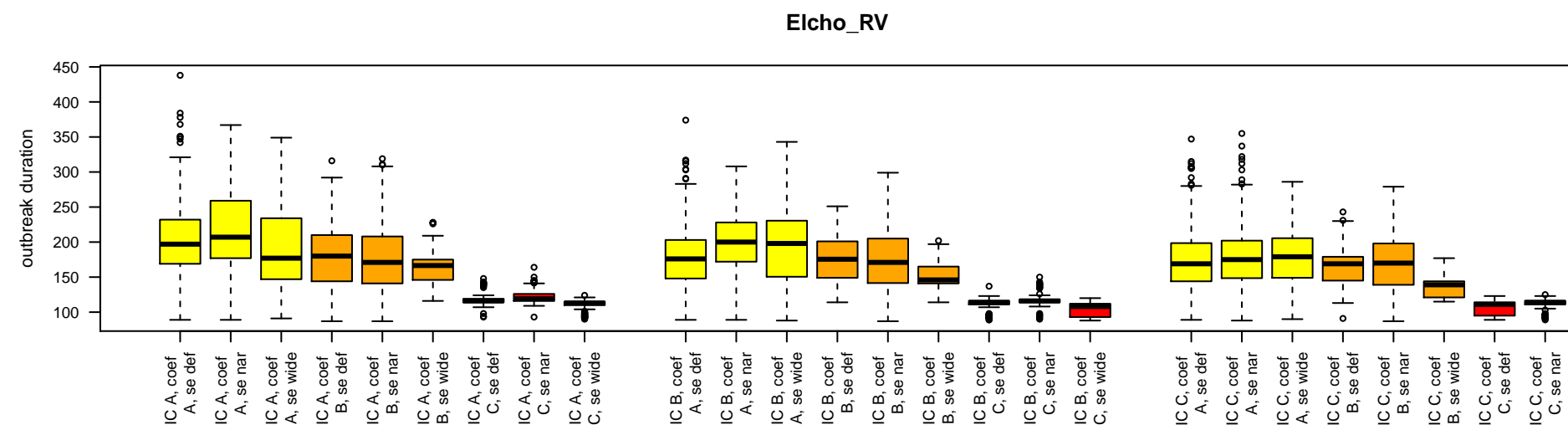

Supplement: S13 Fig — The three parameters are the intercept (IC), coefficient (coef) and standard error of the coefficient (se) and their distinct values on the x-axis were: IC 0.439 (A), 0.878 (B) and 1.318 (C); coef: -0.0174 (A), -0.0116 (B), -0.0058 (C); and se as “default” (def), “narrow” (nar) or “wide” (wide). See main text for further details. The boxes in the boxplots represent the interquartile range (IQR), the horizontal line in the box the median and the whiskers extend to the most extreme data point which is no more than 1.5 times IQR from the box. (A) Dependency of the number of rabid dogs on the three parameters defining the distance kernel for NPA; (B) dependency of the number of rabid dogs on the three parameters defining the distance kernel for Elcho Island; (C) dependency of the outbreak duration on the three parameters defining the distance kernel for the NPA; and (D) dependency of the outbreak duration on the three parameters defining the distance kernel for Elcho Island. (PDF) [file pntd.0003876.s013.pdf]
