# Supplementary material for: Development of a Novel Rabies Simulation Model for Application in a Non-endemic Environment
Source: PLoS Negl Trop Dis. 2015 Jun 26;9(6):e0003876. doi: 10.1371/journal.pntd.0003876 (PMC4482682; doi:10.1371/journal.pntd.0003876)
Supplement: S4 Fig — Both the mode or mean and the shape of the parameters were varied ±10% around their default values (for the vaccine efficacy ±4%). The variation in the shape is defined as varying the difference between the mode or mean to the upper and lower limit of the beta-pert or uniform distribution, respectively. The continuous line represents the default value of the shape, the dashed line the narrow shape and the dotted line the wide shape. (A) incubation period, (B) rabies transmission probability given a bite, (C) probability of being bitten given a contact between two dogs from different households (hh) and (D) vaccine efficacy. (PDF) [file pntd.0003876.s004.pdf]

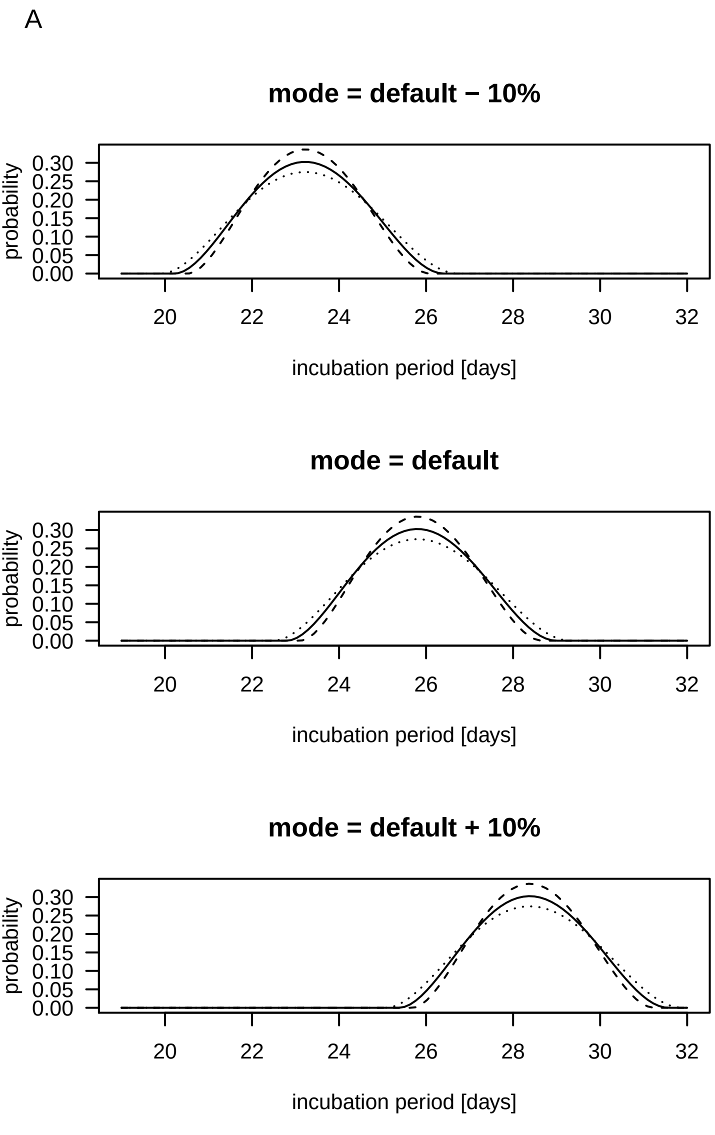
A
mode = default − 10%
probability
0.30
0.25
0.20
0.15
0.10
0.05
0.00
20
22
24
26
28
30
32
incubation period [days]
mode = default
probability
0.30
0.25
0.20
0.15
0.10
0.05
0.00
20
22
24
26
28
30
32
incubation period [days]
mode = default + 10%
probability
0.30
0.25
0.20
0.15
0.10
0.05
0.00
20
22
24
26
28
30
32
incubation period [days]

B

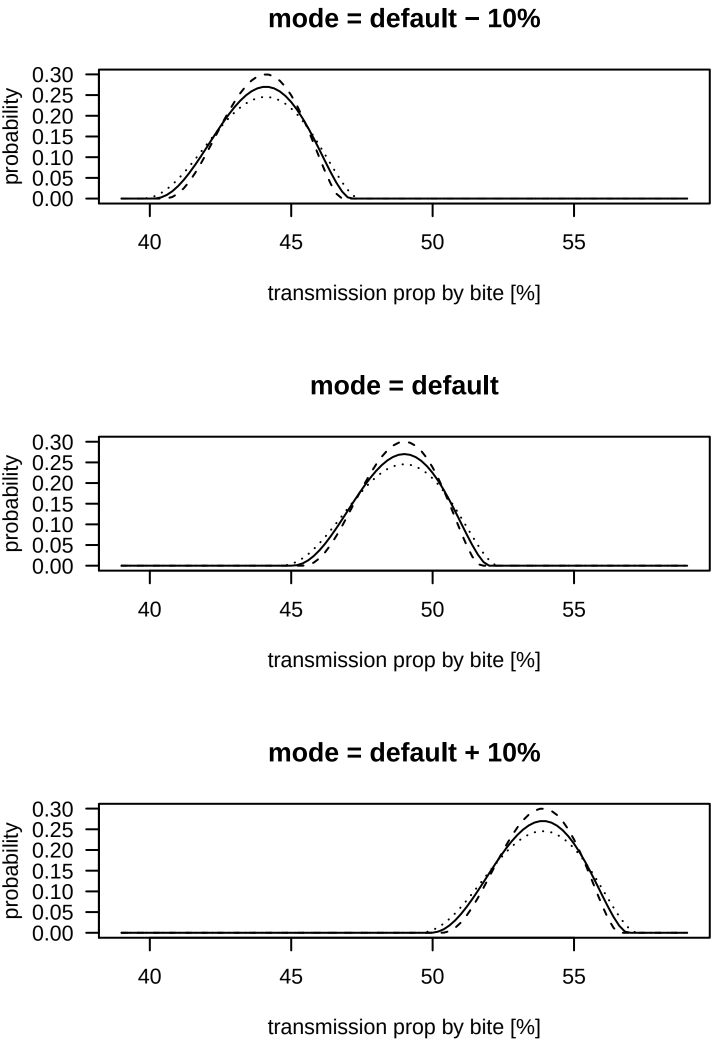
mode = default − 10%
probability
0.30
0.25
0.20
0.15
0.10
0.05
0.00
40
45
50
55
transmission prop by bite [%]
mode = default
probability
0.30
0.25
0.20
0.15
0.10
0.05
0.00
40
45
50
55
transmission prop by bite [%]
mode = default + 10%
probability
0.30
0.25
0.20
0.15
0.10
0.05
0.00
40
45
50
55
transmission prop by bite [%]

C

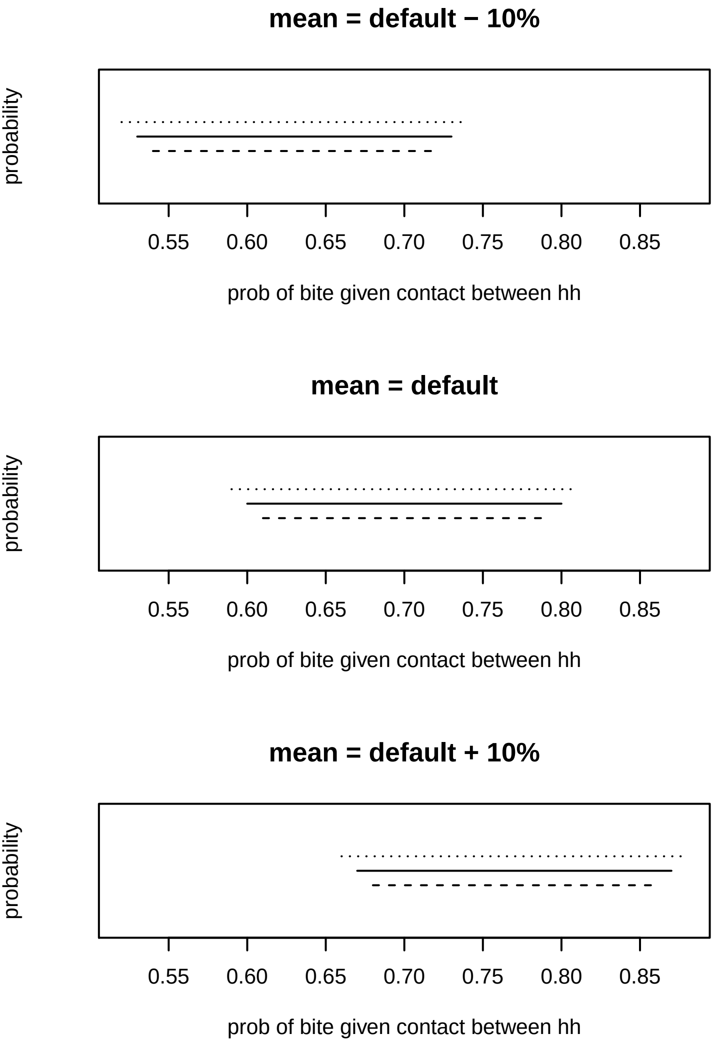
mean = default − 10%
probability
0.55
0.60
0.65
0.70
0.75
0.80
0.85
prob of bite given contact between hh
mean = default
probability
0.55
0.60
0.65
0.70
0.75
0.80
0.85
prob of bite given contact between hh
mean = default + 10%
probability
0.55
0.60
0.65
0.70
0.75
0.80
0.85
prob of bite given contact between hh

D

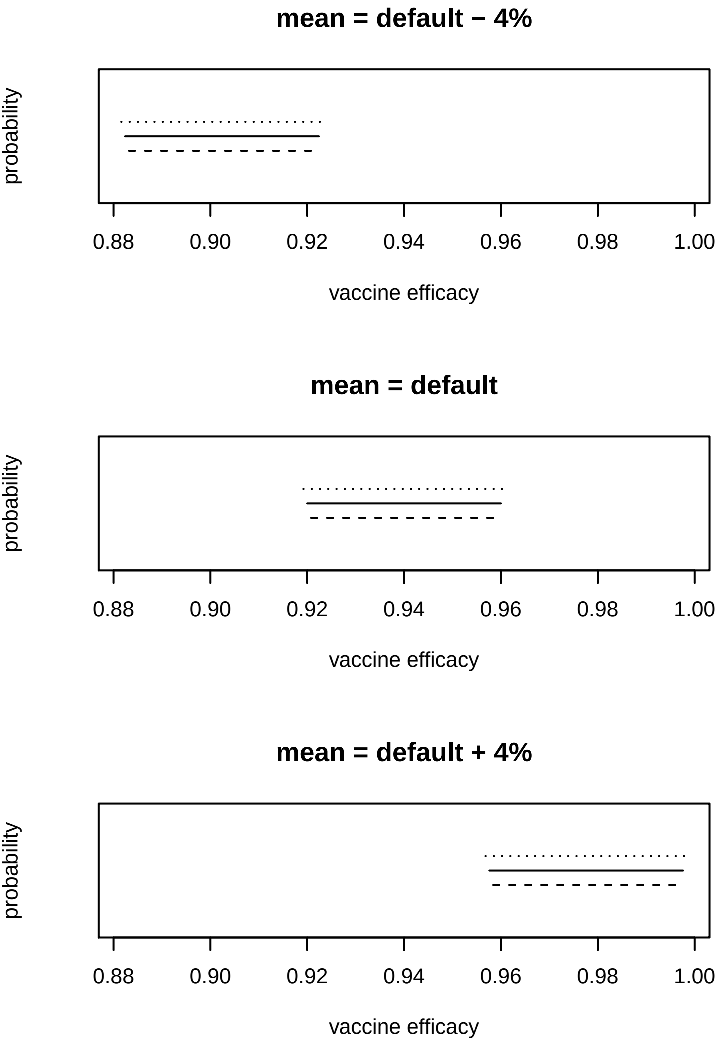
mean = default − 4%
probability
0.88
0.90
0.92
0.94
0.96
0.98
1.00
vaccine efficacy
mean = default
probability
0.88
0.90
0.92
0.94
0.96
0.98
1.00
vaccine efficacy
mean = default + 4%
probability
0.88
0.90
0.92
0.94
0.96
0.98
1.00
vaccine efficacy
