# Supplementary material for: Development of a Novel Rabies Simulation Model for Application in a Non-endemic Environment
Source: PLoS Negl Trop Dis. 2015 Jun 26;9(6):e0003876. doi: 10.1371/journal.pntd.0003876 (PMC4482682; doi:10.1371/journal.pntd.0003876)
Supplement: S12 Fig — The x-axis represents the value of the mode (A/B, E/F) or mean (C/D, G) of the parameter and the terms “default”, “narrow” and “wide” describe the shape; see main text for further details. The boxes in the boxplots represent the interquartile range (IQR), the horizontal line in the box the median and the whiskers extend to the most extreme data point which is no more than 1.5 times IQR from the box. (A) Dependency of the number of rabid dogs on the incubation period; (B) dependency of the outbreak duration on the incubation period; (C) dependency of the number of rabid dogs on the probability of being bitten given a contact between dogs of different households; (D) dependency of the outbreak duration on the probability of being bitten given a contact between dogs of different households; (E) dependency of the number of rabid dogs on the probability of rabies transmission given a bite; (F) dependency of the outbreak duration on the probability of rabies transmission given a bite; and (G) dependency of the number of rabid dogs (upper half) and outbreak duration (lower half) on the vaccine efficacy. (PDF) [file pntd.0003876.s012.pdf]

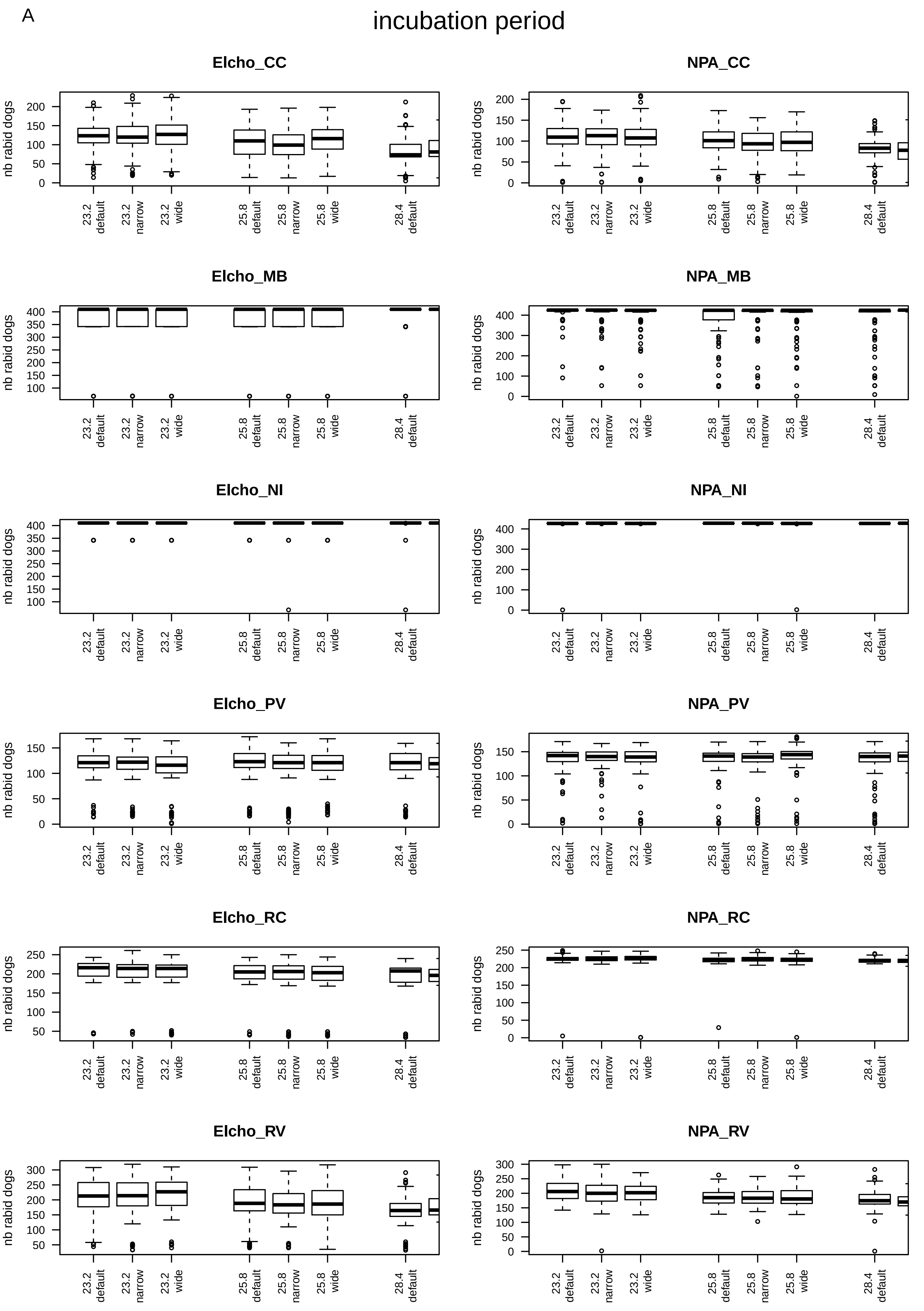
A
incubation period
Elcho_CC
NPA_CC
Elcho_MB
NPA_MB
Elcho_NI
NPA_NI
Elcho_PV
NPA_PV
Elcho_RC
NPA_RC
Elcho_RV
NPA_RV
nb rabid dogs
23.2 default
23.2 narrow
23.2 wide
25.8 default
25.8 narrow
25.8 wide
28.4 default

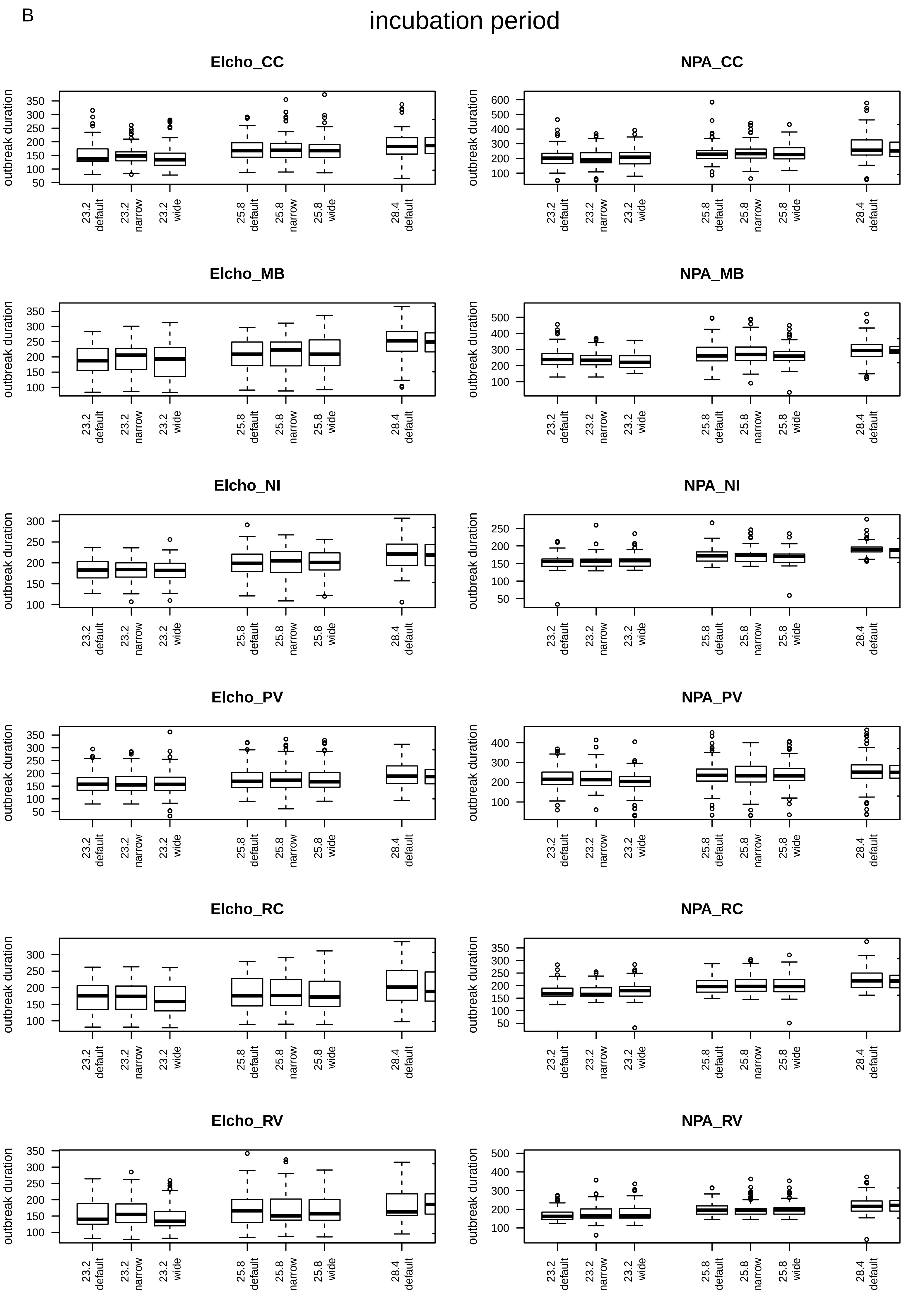
B
incubation period
Elcho_CC
NPA_CC
Elcho_MB
NPA_MB
Elcho_NI
NPA_NI
Elcho_PV
NPA_PV
Elcho_RC
NPA_RC
Elcho_RV
NPA_RV
outbreak duration
23.2 default
23.2 narrow
23.2 wide
25.8 default
25.8 narrow
25.8 wide
28.4 default

C

bite prob of contacts between dogs of different households

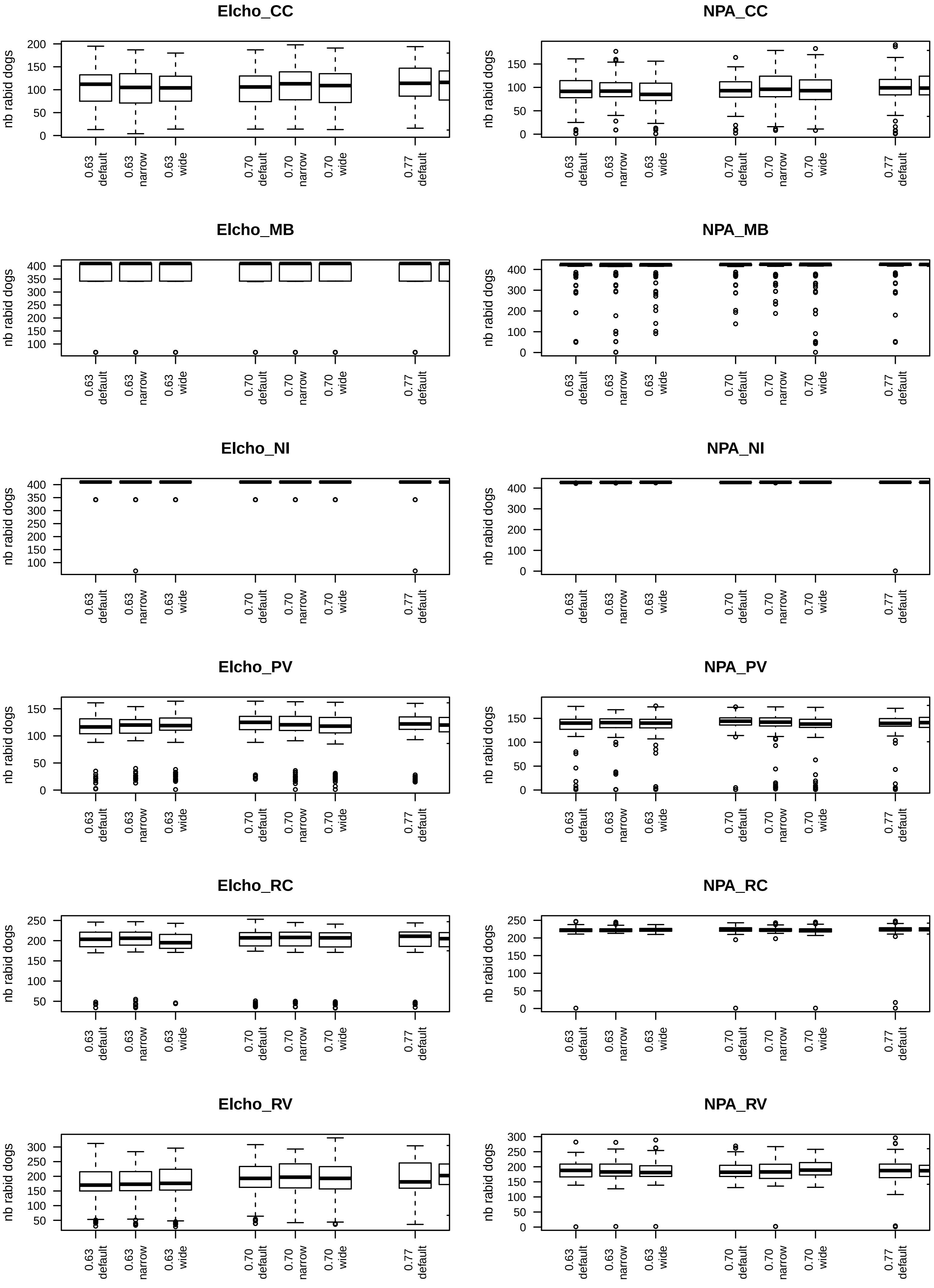

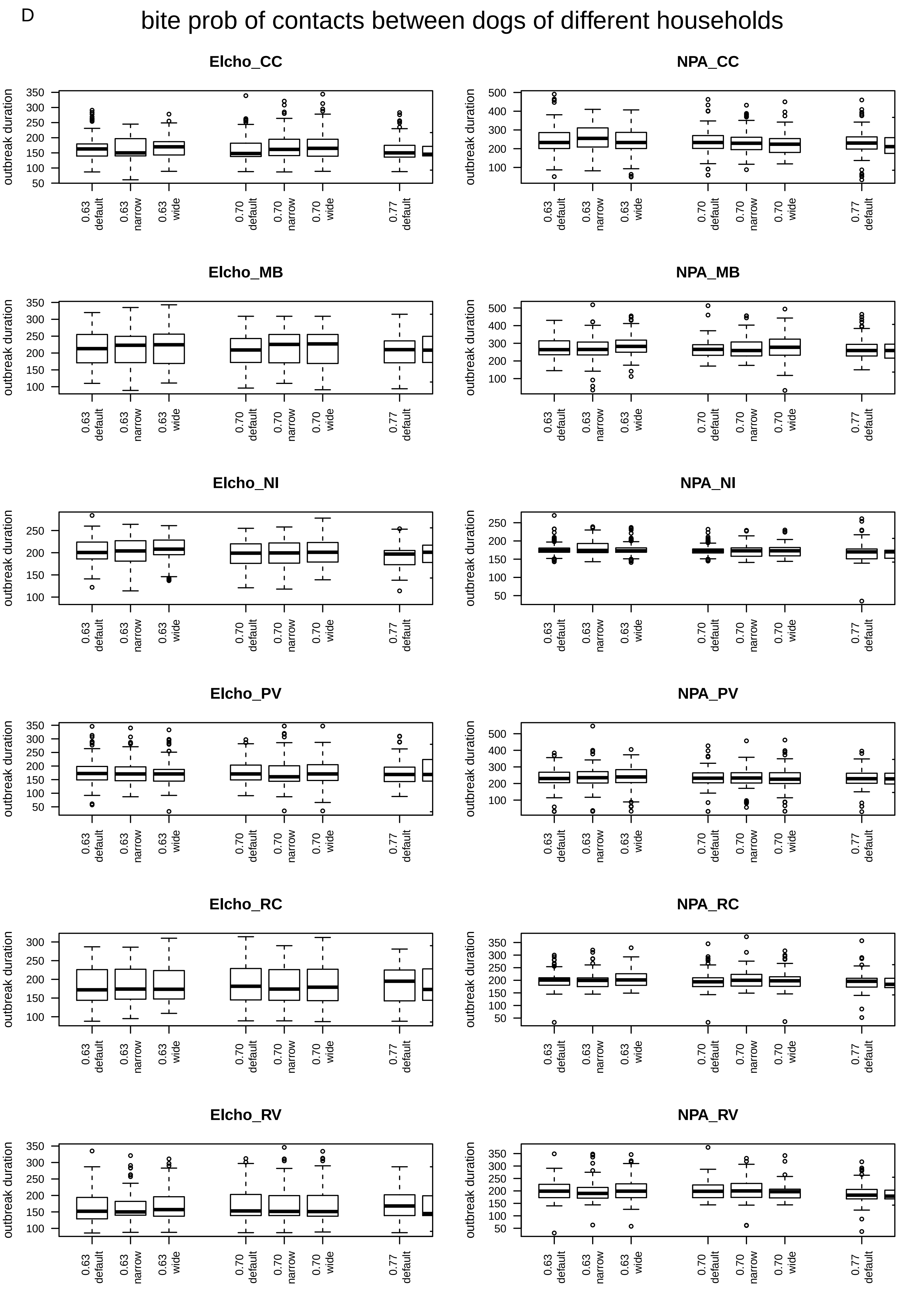
D
bite prob of contacts between dogs of different households
Elcho_CC
NPA_CC
Elcho_MB
NPA_MB
Elcho_NI
NPA_NI
Elcho_PV
NPA_PV
Elcho_RC
NPA_RC
Elcho_RV
NPA_RV
outbreak duration
0.63 default
0.63 narrow
0.63 wide
0.70 default
0.70 narrow
0.70 wide
0.77 default

E

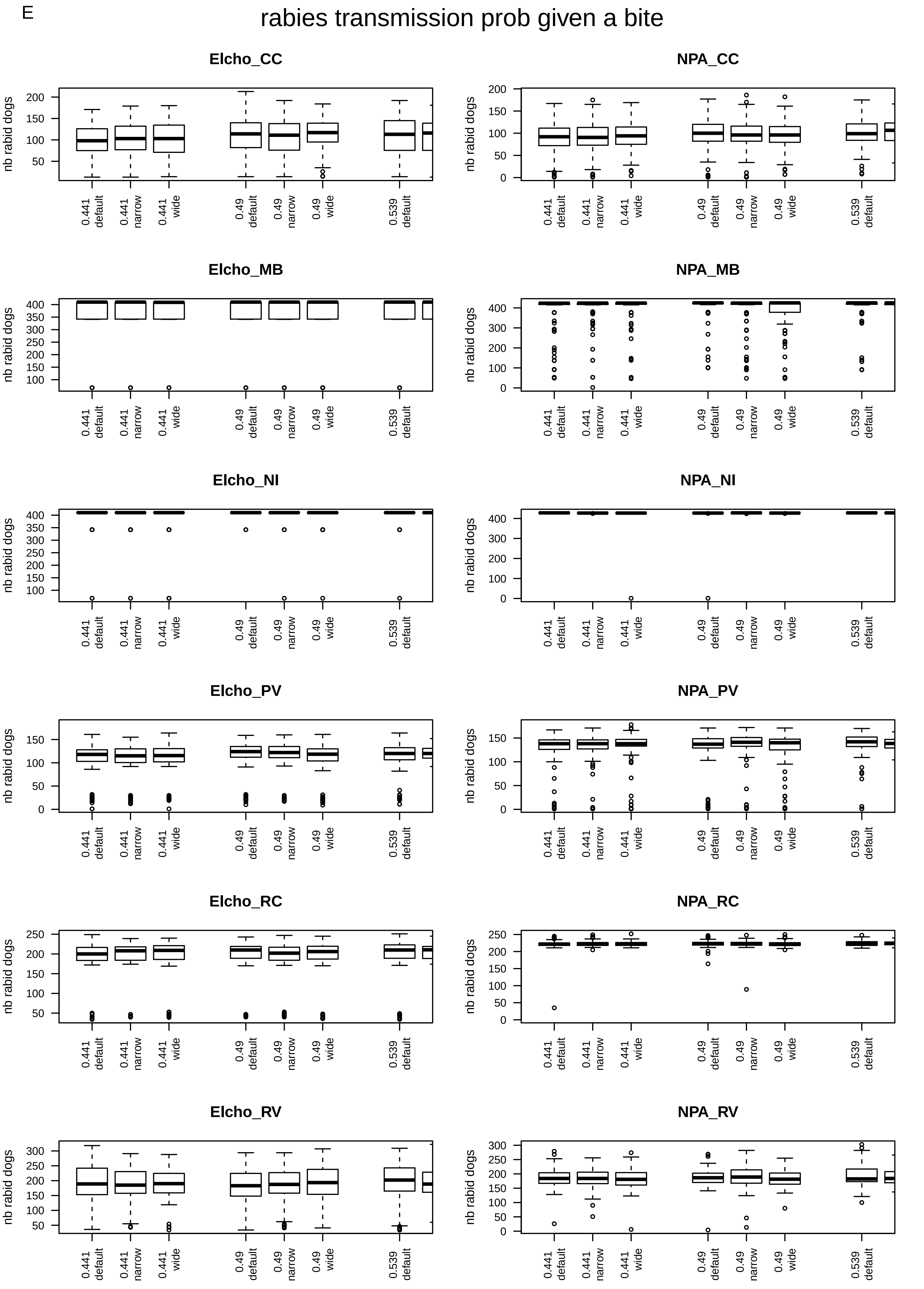
rabies transmission prob given a bite
Elcho_CC
NPA_CC
Elcho_MB
NPA_MB
Elcho_NI
NPA_NI
Elcho_PV
NPA_PV
Elcho_RC
NPA_RC
Elcho_RV
NPA_RV
nb rabid dogs
0.441 default
0.441 narrow
0.441 wide
0.49 default
0.49 narrow
0.49 wide
0.539 default

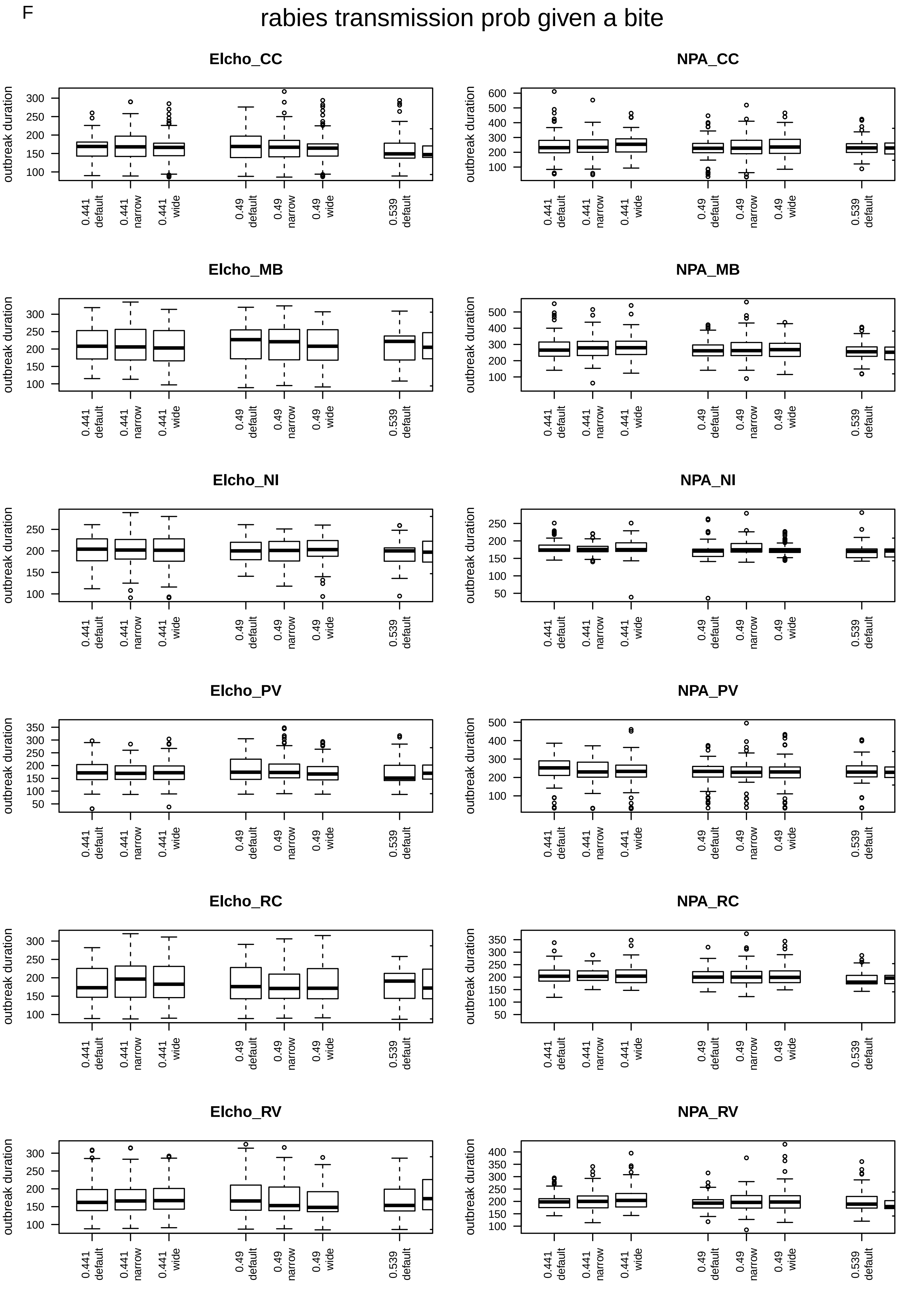
F
rabies transmission prob given a bite
Elcho_CC
NPA_CC
Elcho_MB
NPA_MB
Elcho_NI
NPA_NI
Elcho_PV
NPA_PV
Elcho_RC
NPA_RC
Elcho_RV
NPA_RV
outbreak duration
0.441 default
0.441 narrow
0.441 wide
0.49 default
0.49 narrow
0.49 wide
0.539 default

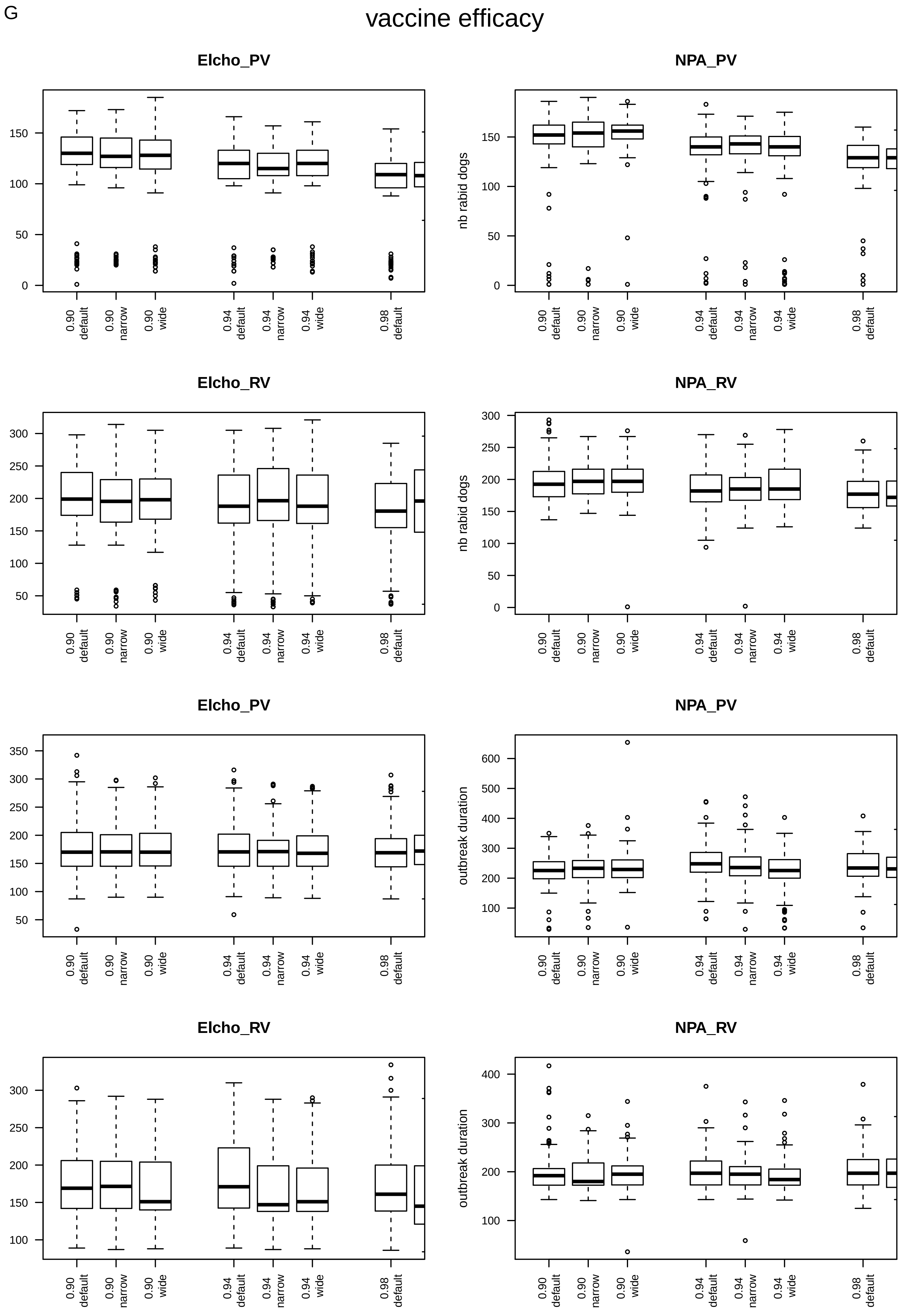
G
vaccine efficacy
Elcho_PV
NPA_PV
Elcho_RV
NPA_RV
nb rabid dogs
outbreak duration
0.90 default
0.90 narrow
0.90 wide
0.94 default
0.94 narrow
0.94 wide
0.98 default
