# Supplementary material for: Development of a Novel Rabies Simulation Model for Application in a Non-endemic Environment
Source: PLoS Negl Trop Dis. 2015 Jun 26;9(6):e0003876. doi: 10.1371/journal.pntd.0003876 (PMC4482682; doi:10.1371/journal.pntd.0003876)
Supplement: S1 Table — Name, description, default values and source of all parameters used in the rabies simulation model; stochastic values either follow a beta-pert distribution (Pert(Min,Mode,Max)) or a uniform distribution (Unif(Min,Max)). (DOCX) [file pntd.0003876.s014.docx]

**Table S1. Model parameters.** Name, description, default values and source of all parameters used in the rabies simulation model; stochastic values either follow a pert distribution (Pert(Min,Mode,Max)) or a uniform distribution (Unif(Min,Max)).

| Parameter | description | Default value | Source |
| --- | --- | --- | --- |
| Max_time | how long (simulated days) the model should run | 365 | ― |
| index dog(s) related parameters | | | |
| Index_region | definition of the case(es) is hierarchical: if index_dog is defined, all other are not used in the model; else if index_community is defined, index_region is not used in the model | NA | ― |
| index_community |  | NA | ― |
| index_dog |  | NA | ― |
| nb_index_dogs |  | 1 | ― |
| disease states related parameters | | | |
| infectiousDelay | Incubation period: time period between exposure (bite) and infectiousness | Pert(22.8,25.8,29) | [1–3] |
| clinicalDelay | Subclinical period (time period between infectiousness and occurrence of typical rabies clinical signs; this parameter mainly influences the detection of rabies as the detection delay starts with the beginning of the clinical phase) | Unif(1,3) | [4,5] |
| mortalityDelay | Clinical period (time period between start of clinical signs and death) | Pert(2,4.7,12) | [1–3] |
| Within and between household contact/transmission related arguments | | | |
| cont_prob_sameHH | Daily contact probability of dogs within the same household | Unif(0.94,1) | Own data |
| DK_intercept, DK_coef, DK_se | Distance kernel parameters defining the probability of a daily contact for households located a given distance apart is pert-distributed with:  mode:$1/\left( {1+e}^{-\left( \alpha+ \beta\times distance \right)} \right)$  min, max: $1/\left( {1+e}^{-\left( \alpha+ \left( \beta\pm1.96\times\beta_{se} \right)\times distance \right)} \right)$  can be defined separately for each community | Intercept α: 1.6567  Coefficient β: -0.0159  Standard error of β β_se_: 0.0026 | Own data |
| bite_prob_sameHH | probability of bite given a contact within the same household | Unif(0.8,0.95) | assumption, high chance of bite assumed as dogs from same household are living close together |
| bite_prob_betweenHH | probability of bite given a contact between dogs from different households | Unif(0.6,0.8) | assumption, less than within household contacts |
| transmissionProb | probability of rabies transmission given a bite (regardless of type of contact) | Pert(0.45,0.49,0.52) | [1] |
| between community movement parameters | | | |
| movements_permanent | daily frequency of permanent movements per dog, can be defined for each community separately | Pert(0.000165, 0.0003,0.00066)  for all communities | Own data |
| movements_shortTerm | daily frequency of short term movements per dog, can be defined for each community separately | Pert(0.015,0.03,0.06) for all communities | Own data |
| visit_period | period (days) the dog remains in the visited community after a short term movement before moving back to its original community | Unif(1,2) | Own data |
| probs | matrix defining the relative probability of between community dog movements from one community to another in the same region | 1 to neighbouring communities; 0.5 to other communities | Own data |
| Rabies detection parameters | | | |
| detectPeriod_firstCase | time (days) for detection of rabies after the start of clinical signs for the first case | Pert(14,21,28) | assumption; relatively short compared to [6] due to increased disease awareness |
| detectPeriod_secondCases | time (days) for detection of rabies after the start of clinical signs for all other cases in the region | Pert(1,2,4) | assumption |
| control strategy vaccination | | | |
| preemptVacc_covLevel_  reference | definition of the vaccination coverage can be based on the dog population (i.e. a given percentage of dogs will be vaccinated irrespectively of whether they are living in the same or different household; "dog") or on the households level (i.e. a given percentage of households will be vaccinated and all dogs in that household are vaccinated; "household") | “household” | Strategy related parameter (adjustable) |
| preemptive_vacc_cov | immunization coverage reached in the region for the pre-emptive vaccination | 0.7 | Strategy related parameter (adjustable) |
| reactVacc_covLevel_  reference | definition of the vaccination coverage can be based on the dog population (i.e. a given percentage of dogs will be vaccinated irrespective of whether they are living in the same or different household; "dog") or on the household level (i.e. a given percentage of households will be vaccinated and all dogs in that household are vaccinated; "household") | “household” | Strategy related parameter (adjustable) |
| Vaccination_goal | vaccination goal is either "community" or "region", indicating whether the goal of the vaccination strategy is to vaccinate all dogs in the community or region where rabies is detected | “region” | Strategy related parameter (adjustable) |
| start_vacc_delay | time (days) between the detection of the first rabid dog and the start of the vaccination campaign | 7 | assumption |
| vacc_capacity | maximum number of dogs vaccinated per day | 50 | assumption |
| reactive_vacc_cov | immunization coverage reached in the community or region by reactive vaccination | 0.7 | Strategy related parameter (adjustable) |
| protectionDelay | delay (days) between vaccination of the dog and the protection of the dog via vaccination. There is no protection from vaccination prior to when protection is reached | Unif(7,14) | [7–9] |
| vaccEfficacy | efficacy of the vaccination if the dog is vaccinated before last_vacc day after the exposure of the dog to rabies | Unif(0.92,0.96) | [7,8,10,11] |
| reduced_vaccEfficacy | efficacy of the vaccination if the dog is vaccinated before last_vacc day after the exposure of the dog to rabies | Unif(0.05,0.25) | assumption |
| late_vacc | number of days determines the change of the vaccination efficacy (from vaccEfficacy to reduced_vaccEfficacy) | Unif(2,4) | [12,13] |
| control strategy culling | | | |
| start_cullDetectDog_delay | time (days) between the detection of the first rabid dog and the start of the immediate culling of rabid dogs | 1 | assumption |
| start_cullContactedDog_delay | time (days) between the detection of the first rabid dog and the start of culling of contacted dogs | 4 | assumption |
| cullProp_contactedDogs | proportion of the dogs contacted by detected rabid dogs that will be culled | 0.8 | assumption |
| start_reactiveCull_delay | time (days) between the detection of the first rabid dog and the start of reactive culling of dogs | 7 | assumption |
| culling_goal | culling goal is either "community" or "region", indicating whether the goal of the culling strategy is to cull all dogs in the community or in the region where rabies is detected | “community” | Strategy related parameter (adjustable) |
| reactiveCull_prop | proportion of the population that will be culled in the reactive culling control option | 0.5 | Strategy related parameter (adjustable) |
| cull_capacity | maximum number of dogs culled per day | 15 | assumption |
| control strategy movement restrictions | | | |
| compliance_BCMovs | dog owners compliance with the movement ban between communities | 0.8 | assumption |
| start_BCban_delay | time (days) between the detection of the first rabies case and the start of the movement bans between communities | 4 | assumption |
| max_dist_BHHban | maximal distance (meters) a dog is allowed to move, i.e. truncation value of the distance kernel; defined per community via communityDistanceKernelsMovements.csv | Bamaga: 26  Injinoo: 31  New Mapoon: 31  Seisia: 23  Umagico: 27  Galiwin’ku: 32 | Own data; extracted from GoogleTM earth Version 7.1.2.2041 |
| compliance_BHHMovs | dog owners compliance with the movement ban between households | 0.6 | assumption |
| start_BHHban_delay | time (days) between the detection of the first rabies case and the start of the movement ban between households | 4 | assumption |

**References**

1. Hampson K, Dushoff J, Cleaveland S, Haydon DT, Kaare M, et al. (2009) Transmission Dynamics and Prospects for the Elimination of Canine Rabies. Plos Biol 7: 462–471.

2. Coleman PG, Dye C (1996) Immunization coverage required to prevent outbreaks of dog rabies. Vaccine 14: 185–186.

3. Foggin CM (1988) Rabies and Rabies-related Viruses in Zimbabwe: historical, virological and ecological aspects University of Zimbabwe, Harare.

4. Beran GW (1994) Rabies and Infection by Rabies-Related Viruses. In: Beran GW, editor. Handbook of Zoonoses, Section B. Viral. Boca Raton, Florida, USA: CRC Press LLC. pp. 307–357.

5. Fekadu M, Shaddock JH (1984) Peripheral distribution of virus in dogs inoculated with two strains of rabies virus. Am J Vet Res 45: 724–729.

6. Townsend SE, Sumantra IP, Pudjiatmoko, Bagus GN, Brum E, et al. (2013) Designing programs for eliminating canine rabies from islands: Bali, Indonesia as a case study. PLoS Negl Trop Dis 7: e2372.

7. Sage G, Khawplod P, Wilde H, Lobaugh C, Menachudha T, et al. (1993) Immune response to rabies vaccine in Alaskan dogs: failure to achieve a consistently protective antibody response. Trans R Soc Trop Med Hyg 87: 593–595.

8. Minke JM, Bouvet J, Cliquet F, Wasniewski M, Guiot AL, et al. (2009) Comparison of antibody responses after vaccination with two inactivated rabies vaccines. Vet Microbiol 133: 283–286.

9. Kallel H, Diouani MF, Loukil H, Trabelsi K, Snoussi MA, et al. (2006) Immunogenicity and efficacy of an in-house developed cell-culture derived veterinarian rabies vaccine. Vaccine 24: 4856–4862.

10. Cliquet F, Verdier Y, Sagné L, Aubert M, Schereffer JL, et al. (2003) Neutralising antibody titration in 25,000 sera of dogs and cats vaccinated against rabies in France, in the framework of the new regulations that offer an alternative to quarantine. Rev Sci Tech 22: 857–866.

11. Sihvonen L, Kulonen K, Neuvonen E, Pekkanen K (1995) Rabies antibodies in vaccinated dogs. Acta Vet Scand 36: 87–91.

12. Clark KA, Wilson PJ (1996) Postexposure rabies prophylaxis and preexposure rabies vaccination failure in domestic animals. J Am Vet Med Assoc 208: 1827–1830.

13. Haupt W (1999) Rabies--risk of exposure and current trends in prevention of human cases. Vaccine 17: 1742–1749.
