# Supplementary material for: Development of a Novel Rabies Simulation Model for Application in a Non-endemic Environment
Source: PLoS Negl Trop Dis. 2015 Jun 26;9(6):e0003876. doi: 10.1371/journal.pntd.0003876 (PMC4482682; doi:10.1371/journal.pntd.0003876)
Supplement: S3 Table — Parameters were tested in all possible scenarios, which were mostly all of the 12 (six control strategies in two regions) but not always (see column Tested scenarios). (DOCX) [file pntd.0003876.s016.docx]

**Table S2. Parameters explored during the first step of the sensitivity analysis (SA) with their default value, and the lower and upper limits of variation during the SA.** Parameters were tested in all possible scenarios, which were mostly all of the 12 (six control strategies in two regions) but not always (see column Tested scenarios).

| Parameter | Default value | Lower limit | Upper limit | Tested scenario^a^ |
| --- | --- | --- | --- | --- |
| infectiousDelay | Pert(22.8, 25.8,29) | Pert(16.4, 19.4,22.6) | Pert(29.3, 32.3,35.5) | all 12 |
| clinicalDelay | Unif(1,3) | Unif(0.5,2.5) | Unif(1.5,3.5) | all 12 |
| mortalityDelay | Pert(2,4.7,12) | Pert(0.8, 3.5,10.8) | Pert(3.2, 5.9,13.2) | all 12 |
| cont_prob_sameHH | Unif(0.94,1) | Unif(0.70,0.76) | Unif(1,1) | all 12 |
| Distance kernel | α: 1.6567  β: -0.0159  β_se_: 0.0026 | α: 0.0902  β: -0.0119  β_se_: 0.0022 | α: 0.1088  β: -0.007  β_se_: 0.0014 | all 12 |
| bite_prob_sameHH | Unif(0.8,0.95) | Unif(0.58,0.73) | Unif(1,1) | all 12 |
| bite_prob_betweenHH | Unif(0.6,0.8) | Unif(0.43,0.63) | Unif(0.78,0.98) | all 12 |
| transmissionProb | Pert(0.45, 0.49,0.52) | Pert(0.33, 0.37,0.40) | Pert(0.57, 0.61,0.64) | all 12 |
| visit_period | Unif(1,2) | Unif(1,1) | Unif(2,3) | all 12 , only NPA^b^ |
| movements_shortTerm | Pert(0.015,0.03,0.06) | Pert(0.0075, 0.0225,0.0525) | Pert(0.0225, 0.0375,0.0675) | all 12 , only NPA |
| movements_permanent | Pert(0.000165, 0.0003,0.00066) | Pert(0.00009, 0.000225, 0.000585) | Pert(0.00024, 0.000375, 0.000735) | all 12 , only NPA |
| probs | 1 to neighbouring communities; 0.5 to other communities | Unique probability to any community | | all 12, only NPA |
| detectPeriod_firstCase | Pert(14,21,28) | Pert(9,16,23) | Pert(19,26,33) | all 12 |
| detectPeriod_secondCases | Pert(1,2,4) | Pert(0,1,3) | Pert(2,3,5) | all 12 |
| start_cullDetectDog_delay | 1 | Unif (0.2) | | all 12 |
| Index community | Randomly chosen among the 5 NPA communities | | | all six , only NPA |
| reactVacc_covLevel_ reference | household | dog | | RV |
| preemptVacc_covLevel_ reference | household | dog | | PV |
| start_vacc_delay | 7 | Unif(5,9) | | RV |
| vacc_capacity | 50 | Unif(38,62) | | RV |
| protectionDelay | Unif(7,14) | Unif(4,11) | Unif(10.17) | RV |
| vaccEfficacy | Unif(0.92,0.96) | Unif(0.69,0.73) | Unif(1,1) | RV, PV |
| reduced_vaccEfficacy | Unif(0.05,0.25) | Unif(0.01,0.21) | Unif(0.09,0.29) | RV |
| late_vacc | Unif(2,4) | Unif (1,3) | Unif(3,5) | RV |
| start_cullContactedDog_delay | 4 | Unif (3,5) | | CC |
| cull_capacity | 15 | Unif(11,19) | | CC, RC |
| start_reactiveCull_delay | 7 | Unif(5,9) | | RC |
| start_BCban_delay | 4 | Unif (3,5) | | MB, only NPA |
| start_BHHban_delay | 4 | Unif (3,5) | | MB |
| max_dist_BHHban | Bamaga: 26  Injinoo: 31  New Mapoon: 31  Seisia: 23  Umagico:27  Galiwin’ku: 32 | Ba: Unif(20,32)  In: Unif(23,39)  NM: Unif(23,39)  Se: Unif(17,29)  Um: Unif(20,34)  Ga: Unif(24,40) | | MB |

^a^NI: non-intervention, RV: reactive vaccination; PV: pre-emptive vaccination; CC: culling of contacted dogs; RC: reactive culling; MB: movement bans (refer to the text for further description)

^b^NPA: Northern Peninsula Area, one of the two study regions
